# Alkyl chain elongation engineering: Unlocking high‐brightness NIR‐II O‐xanthene dyes for in vivo bioimaging

**DOI:** 10.1002/smo2.70117

**Published:** 2026-10-29

**Authors:** Yu Zhao, Hong Zhang, Ruirui Zhang, Wenli Xia, Zhouyu Wang, Jie Chen, Xiaoqi Yu, Kun Li

**Affiliations:** ^1^ Key Laboratory of Green Chemistry and Technology Ministry of Education College of Chemistry Sichuan University Chengdu China; ^2^ Department of Chemistry Sichuan Engineering Research Center of Molecular Targeted Diagnostic & Therapeutic Drugs Xihua University Chengdu China

**Keywords:** anti‐quenching, high brightness, NIR‐II fluorescence imaging, O‐xanthene

## Abstract

NIR‐II fluorescent probes are powerful tools for advanced imaging, with performance governed by molar extinction coefficient (ε), quantum yield (Φ), and brightness (ε × Φ). However, achieving high brightness remains a major challenge. Here we report SCU‐OX‐Hex, a D–A–D probe with an O‐xanthene acceptor and an N‐alkylated aniline donor. Elongating the N‐alkyl chain enhances Φ and brightness while suppressing aggregation‐caused quenching. SCU‐OX‐Hex exhibits ε = 1.71 × 10^5^ M^−1^ cm^−1^, Φ = 0.57%, and a brightness of 974.7 M^−1^ cm^−1^—among the highest reported for organic NIR‐II probes. In vivo, it enables high‐resolution imaging of the abdominal vasculature, the gastrointestinal tract, and tumor angiogenesis. In a lymph node metastasis model, it provides superior contrast and resolution compared with ICG, enabling precise intraoperative identification. This work establishes SCU‐OX‐Hex as a high‐performance NIR‐II agent and offers a broadly applicable design strategy for next‐generation probes.

## Graphical abstract

O‐xanthene–based fluorophore enhanced fluorescence quantum yield and brightness were achieved by extending the N‐alkyl chain on the aniline donor.

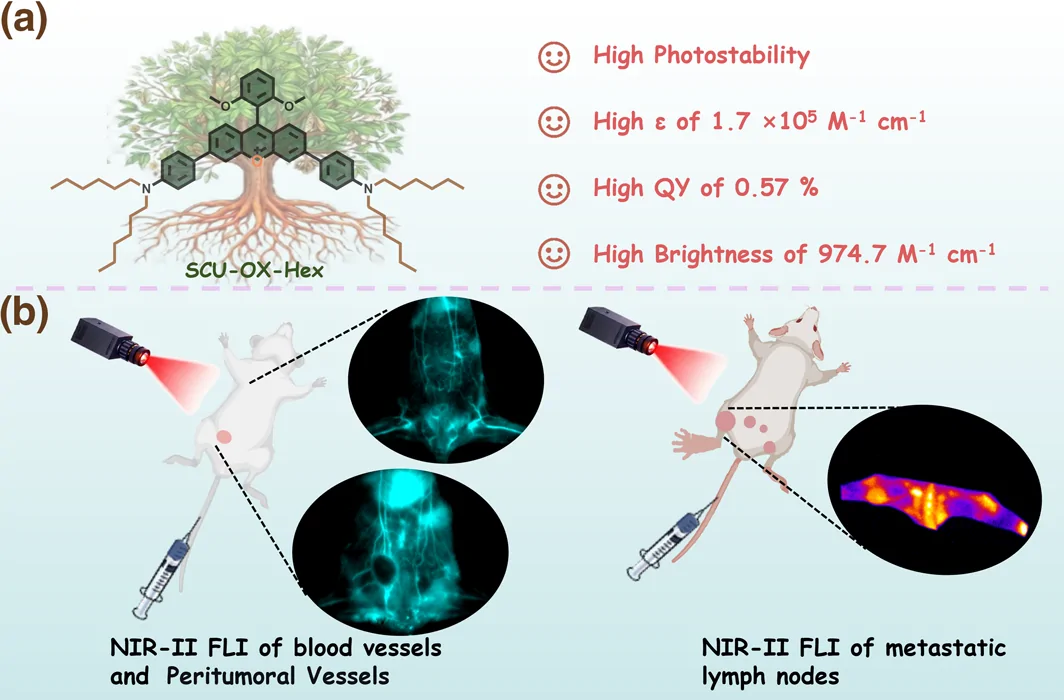

## 1 INTRODUCTION

Continuous advances in biological imaging have substantially improved the precision of disease diagnosis and provided essential support for the design and refinement of personalized therapeutic regimens.^[1]^ Among various emerging imaging modalities, fluorescence imaging (FLI) has attracted particular attention due to its high sensitivity, high specificity, non‐invasiveness, and capacity for real‐time dynamic visualization.[^2^, ^3^] Near‐infrared II (NIR‐II, 1000–1700 nm) imaging, in particular, is recognized as a promising direction for next‐generation optical imaging, benefiting from deeper tissue penetration, superior spatial resolution, and minimized autofluorescence interference.[^4^, ^5^, ^6^, ^7^, ^8^, ^9^, ^10^, ^11^, ^12^, ^13^, ^14^] Achieving high‐performance NIR‐II imaging critically depends on the development of fluorescent probes with high brightness, which is governed by both the ε and fluorescence QY (Φ).[^15^, ^16^, ^17^] High‐brightness probes can effectively suppress background noise and compensate for photon attenuation caused by tissue scattering and absorption. Nevertheless, most currently reported NIR‐II fluorophores suffer from intrinsically low ε (typically below 10^5^ M^−1^ cm^−1^) and low Φ (frequently below 0.05%, comparable to that of the commercial dye IR26).[^18^, ^19^, ^20^] These deficiencies severely limit their translational potential in clinical diagnostics. Consequently, the design of NIR‐II probes simultaneously exhibiting high ε, high Φ, and exceptional brightness remains a critical and unmet challenge in the field.

To overcome these inherent photophysical limitations, various molecular engineering strategies have been explored to synergistically enhance ε, Φ, and overall brightness (Supporting Information S1: Table S1). For instance, the yang group constructed IR‐FCT8CP, an amphiphilic molecule incorporating branched alkyl‐substituted TQT units as an alternative to BBTD. In aqueous media, this probe showed an ε of 1.67 × 10^4^ M^−1^ cm^−1^, a Φ of 0.05%, and a brightness of 8.4 M^−1^ cm^−1^.^[21]^ In another example, the Lee group developed.

DETTD by employing thieno[3,4‐c][1,2,5]thiadiazole (TTD) as an acceptor unit in combination with conformationally locked donors and flexible alkyl side chains DETTD exhibited an ε of 3.11 × 10^4^ M^−1^ cm^−1^, a Φ of 2.52%, and a brightness of 783.7 M^−1^ cm^−1^.^[22]^ Despite these advances, the concurrent achievement of ε exceeding 10^5^ M^−1^ cm^−1^, high Φ, and high brightness—particularly in organic small‐molecule systems—remains a formidable obstacle.

Xanthene‐based dyes have been extensively employed in biomedical research owing to their high molar extinction coefficients, structural tunability, and excellent photostability.[^23^, ^24^, ^25^, ^26^, ^27^, ^28^, ^29^, ^30^, ^31^] However, conventional xanthene derivatives primarily emit within the visible or NIR‐I region. When extended to the NIR‐II window, they typically exhibit reduced ε and severely quenched fluorescence, resulting in insufficient brightness for deep‐tissue imaging applications.[^32^, ^33^, ^34^, ^35^, ^36^, ^37^, ^38^] For example, our previous work reported SCU‐SX‐T, which utilizes S‐xanthene as an electron acceptor and diphenylamine as a rotor. Although SCU‐SX‐T achieved an impressive ε of 2.0 × 10^5^ M^−1^ cm^−1^, its Φ was merely 0.04%, yielding a brightness of only 80 M^−1^ cm^−1^.^[39]^

Similarly, Yang ’s group developed EC7 through tetra‐benzannulation of the xanthene core, attaining an ε of 3.91 × 10^5^ M^−1^ cm^−1^—the highest reported to date among xanthene‐based NIR‐II fluorophores. Nevertheless, its Φ remained marginal at 0.053%, leading to a brightness of 207 M^−1^ cm^−1^.^[40]^ These cases illustrate a recurring trade‐off: strategies aimed at reinforcing molecular planarity to boost ε often exacerbate intermolecular π–π stacking and aggregation‐induced fluorescence quenching, thereby compromising Φ and overall brightness. Thus, achieving a simultaneous increase in ε, suppression of aggregation, and preservation or enhancement of Φ and brightness is essential for advancing NIR‐II xanthene probes.

To address these challenges, we rationally designed and synthesized a novel NIR‐II fluorescent probe, SCU‐OX‐Hex, through a targeted molecular engineering strategy (Scheme 1). The probe features O‐xanthene as the conjugated electron acceptor and aniline as the electron donor. Elongation of the N‐alkyl chain on the aniline donor substantially enhanced the fluorescence QY and molecular brightness while effectively suppressing aggregation‐caused quenching. The optimized SCU‐OX‐Hex exhibits a high ε (1.71 × 10^5^ M^−1^ cm^−1^), a remarkable NIR‐II fluorescence QY (Φ = 0.57%), and an exceptional brightness of 974.7 M^−1^ cm^−1^. Density functional theory calculations and molecular dynamics (MD) simulations revealed that the aniline substituent does not alter the twisted intramolecular conformation. Instead, the extended N‐alkyl chain promotes looser molecular packing in solution, mitigating deleterious π–π interactions and thereby enhancing Φ. In vivo NIR‐II imaging demonstrated that SCU‐OX‐Hex enables high‐contrast visualization of abdominal vasculature and gastrointestinal structures in mice, and permits dynamic monitoring of tumor angiogenesis and vascular invasion at distinct disease stages. In a lymph node metastasis model, SCU‐OX‐Hex afforded markedly improved imaging contrast and spatial resolution for both metastatic sciatic and popliteal lymph nodes compared with the clinically approved indocyanine green (ICG).

**SCHEME 1:**
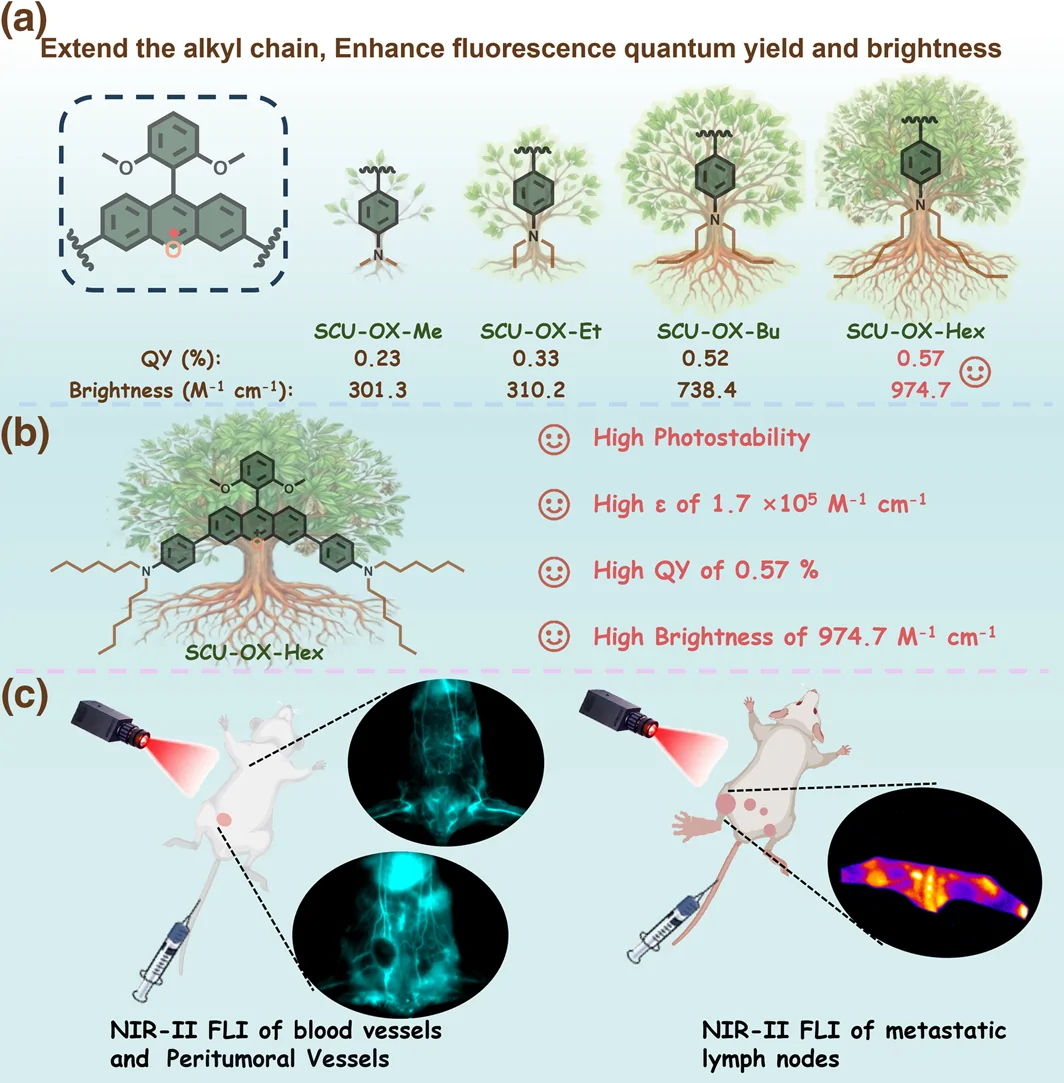
(a) Schematic illustrations of the molecule design structure for SCU‐OXs fluorescence probes. (b) The chemical structure and design concept of representative SCU‐OX‐Hex, (c) In vivo fluorescence imaging (FLI).

This facilitated precise intraoperative identification and resection of metastatic nodes. Collectively, these results establish SCU‐OX‐Hex as a highly promising NIR‐II fluorophore with exceptional in vivo imaging performance. This work presents a compelling molecular design strategy for developing high‐brightness NIR‐II xanthene probes and provides valuable insight into the rational engineering of next‐generation high‐performance NIR‐II imaging agents.

## 2 RESULT AND DISCUSSION

### 2.1 Molecular design, synthesis, and characterization

The SCU‐OX family of NIR‐II fluorophores was engineered from an O‐xanthene core by introducing strongly electron‐donating 4‐N,N‐dimethylamino groups at the 3,6‐positions, thereby establishing a donor–acceptor–donor (D–A–D) structure that effectively shifts the emission profile into the NIR‐II window. Incorporation of the aniline moiety maintains the intrinsic rigid planar conformation of the xanthene backbone, a feature that favors high molar absorptivity and efficient photon capture. Despite these advances, the initial derivative SCU‐OX‐Me suffers from pronounced intermolecular interactions, which induce severe aggregation‐caused quenching and result in a low fluorescence QY and diminished brightness in the NIR‐II region. To overcome these limitations, we implemented a rational alkyl‐chain extension at the donor termini (the 4‐N,N‐dimethylamino groups), yielding the optimized fluorophore SCU‐OX‐Hex (Figure 1a). This structural modification effectively suppresses intermolecular stacking and restricts intramolecular motion, thereby significantly enhancing the resistance to aggregation‐caused quenching. The synthesis of this series was accomplished via a key Suzuki cross‐coupling step, delivering the target compounds in high overall yields (Supporting Information S1: Scheme S1). All intermediates and final products were unambiguously characterized by ^1^H NMR, ^13^C NMR, ^19^F NMR and high‐resolution mass spectrometry (Supporting Information S1: Figures S1–S25).

**FIGURE 1:**
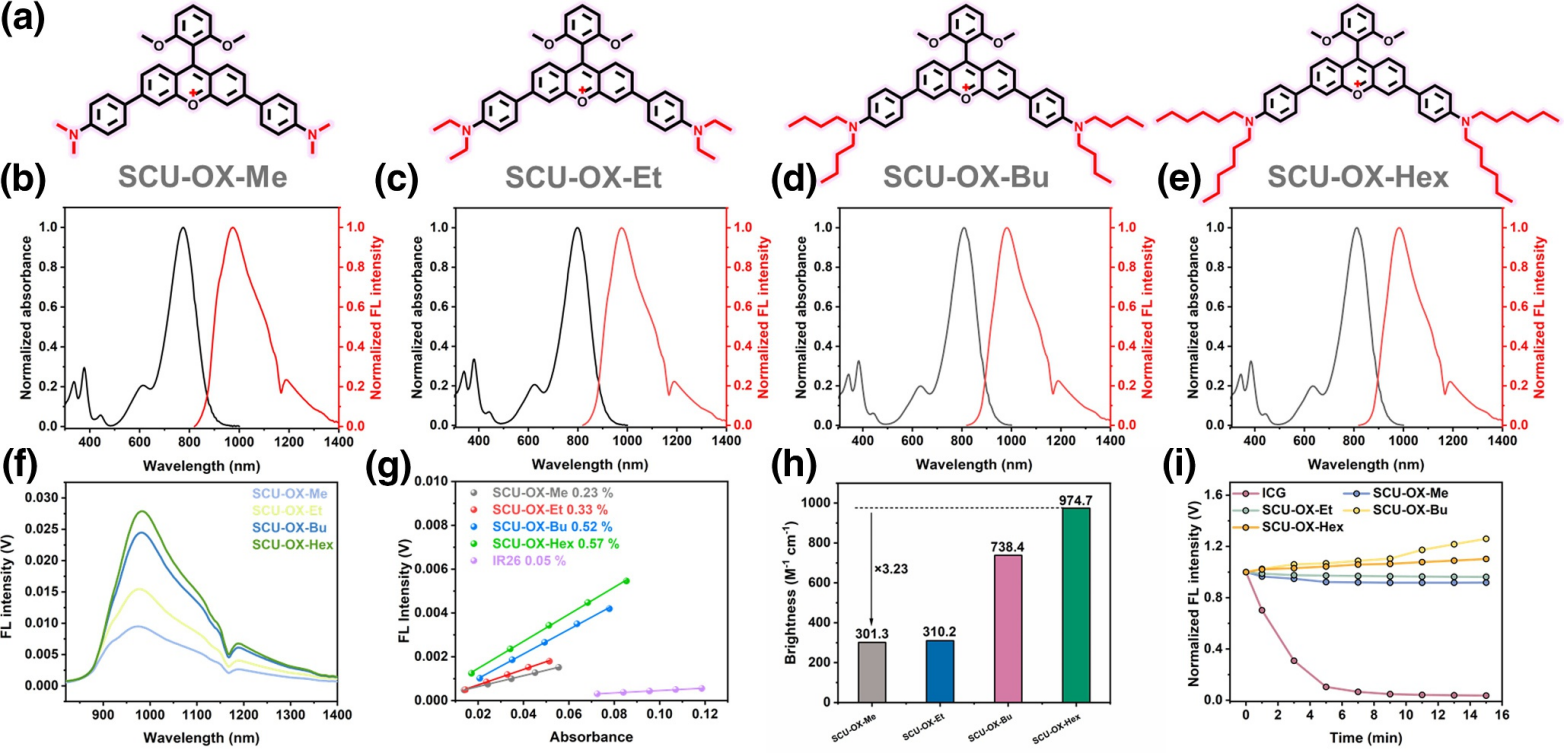
The structure of four compounds (a), and normalized absorption and emission in DCE solvent (b–e). Comparison of emission intensity (f), fluorescence QY (g), and brightness (h) of four compounds in DCE solvent. (i) Photostability of four compounds and ICG upon 808 nm laser irradiation (0.6 W cm^−2^).

### 2.2 Photophysical property

We investigated the photophysical behavior of SCU‐OX‐Me, SCU‐OX‐Et, SCU‐OX‐Bu, and SCU‐OX‐Hex in various solvents, revealing a solvent‐dependent effect of alkyl chain length on quantum yield (Φ) (Table 1, Supporting Information S1: Figures S26 and S27). In DCE, all compounds showed NIR‐I absorption and emission centered at ∼980 nm (Figure 1b–e). SCU‐OX‐Hex exhibited a high molar extinction coefficient (1.71 × 10^5^ M^−1^ · cm^−1^, Supporting Information S1: Figure S28) and the highest Φ (0.57%, Figure 1g, Supporting Information S1: Figure S29), yielding a molecular brightness of 974.7 M^−1^ · cm^−1^ (Figure 1h). At equivalent concentrations, SCU‐OX‐Hex also displayed higher fluorescence intensity (Figure 1f). Absorption and emission spectra measured in an aqueous formulation (10% DMSO and 2% Tween 80) closely matched those in DCE, confirming that this medium does not alter the intrinsic spectral properties (Supporting Information S1: Figure S30). This enhancement arises from restriction of intramolecular motion by longer alkyl chains and not from suppression of π–π stacking, as confirmed by concentration‐dependent and viscosity measurements (Supporting Information S1: Figures S31 and S32). In aqueous media, the Φ order shifted to Bu > Hex > Et > Me (Supporting Information S1: Figure S33), where hydrophobic shielding governs: butyl provides an optimal balance; ethyl offers insufficient shielding; hexyl induces loose aggregation and conformational dynamics that introduce non‐radiative pathways. The compounds showed superior photostability to ICG under 808 nm irradiation (Figure 1i). Fluorescence and dynamic light scattering measurements upon interaction with human serum albumin (HSA) suggested that these compounds do not form stable complexes with the protein, rather short‐chain molecules may enter the hydrophobic cavity of HSA, thereby improving their solubilization (Supporting Information S1: Figures S34–S36). Collectively, chain length modulates aggregation state and molecular motion, jointly determining the emission behavior across environments.

**TABLE 1:** Photophysical data of all compounds in DCE.

Dye	λ_abs_ (nm)	λ_em_ (nm)	ε (M^−1^ cm^−1^)	Φ (%)	Brightness (M^−1^ cm^−1^)
SCU‐OX‐Me	776	974	1.31 × 10^5^	0.23	301.3
SCU‐OX‐Et	800	976	0.94 × 10^5^	0.33	310.2
SCU‐OX‐Bu	810	982	1.42 × 10^5^	0.52	738.4
SCU‐OX‐Hex	812	984	1.71 × 10^5^	0.57	974.7

*Note*: Represents the spectra of four compound tests in DCE solvent.

### 2.3 Theoretical calculation and molecular dynamics simulation

To further investigate the relationship between probe structure and optical properties. Employed density functional theory calculations at the B3LYP/6‐31G(d) level to elucidate the ground‐state electronic structures of the four fluorophores. As shown in Figure 2a,c,e,g, the HOMO and LUMO are both delocalized over the entire π‐conjugated backbone; however, their electron density distributions exhibit a clear primary–secondary difference: the HOMO electron density is predominantly concentrated on the donor moiety, while the LUMO tends to be more localized on the xanthene‐based acceptor core. The HOMO–LUMO energy gaps remain virtually invariant across the series, ranging from 1.89 to 1.92 eV. This invariance arises because the electron‐donating strength is governed primarily by the nitrogen atom and its two adjacent methylene units; elongation of the N‐alkyl chains does not appreciably alter the donor capacity but instead modulates the degree of intermolecular aggregation in solution.

**FIGURE 2:**
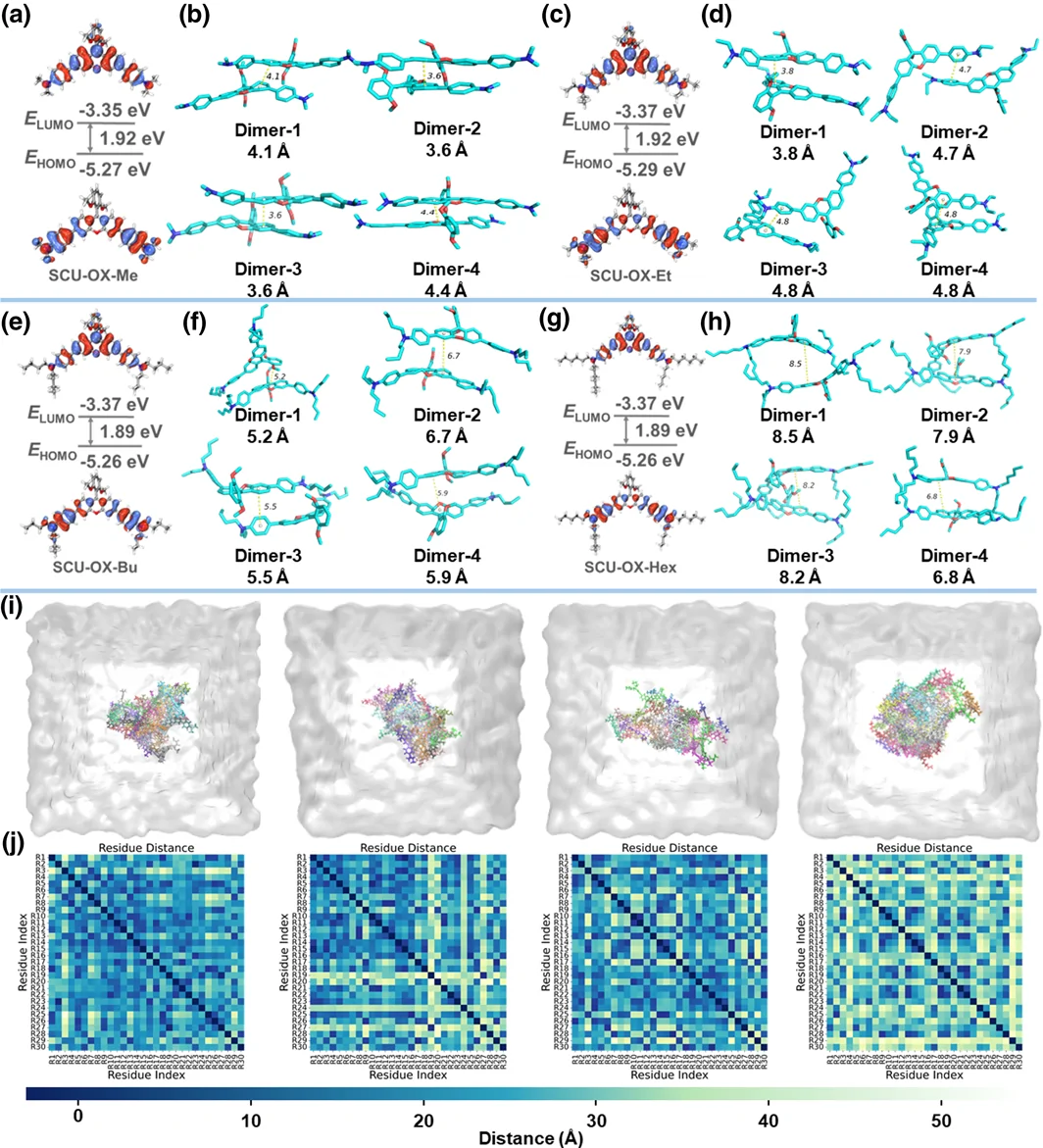
(a–h) HOMO–LUMO spatial distributions, corresponding energy levels, energy gaps, and representative dimer configurations extracted from molecular dynamics (MD) simulations in aqueous environment for SCU‐OX‐Me (a), (b), SCU‐OX‐Et (c), (d), SCU‐OX‐Bu (e), (f), and SCU‐OX‐Hex (g), (h). (i) Typical aggregated structures of SCU‐OX‐Me, SCU‐OX‐Et, SCU‐OX‐Bu, and SCU‐OX‐Hex obtained from 20 ns MD simulation trajectories in aqueous solution. (j) Residue‐specific centroid distance plots derived from 30 individual molecules of SCU‐OX‐Me, SCU‐OX‐Et, SCU‐OX‐Bu, and SCU‐OX‐Hex under aqueous conditions.

To gain quantitative insight into how alkyl‐chain length influences aggregation behavior, initial configurations were constructed using the PACKMOL program by randomly placing 30 molecules of each derivative into a cubic simulation box (8 × 8 × 8 nm), followed by MD simulations. As shown in Figure 2b–d, SCU‐OX‐Me and SCU‐OX‐Et form densely packed aggregates with typical dimer intermolecular distances below 5 Å. In striking contrast, SCU‐OX‐Bu and SCU‐OX‐Hex adopted much more dispersed arrangements, exhibiting dimer separations exceeding 5 Å (Figure 2f–h). Notably, SCU‐OX‐Hex, benefiting from its extended N‐alkyl chains, displays intermolecular distances uniformly above 7 Å. Time‐resolved snapshots extracted from the simulation trajectories further corroborate that longer alkyl chains promote looser molecular packing in aqueous environments (Figure 2i and Supporting Information S1: Figure S37). Quantitative analysis of the spatial distribution was performed by calculating the distance of each molecule from the center of mass of the simulation box using Python. As shown in Figure 2j, the average centroid distance increases progressively with the alkyl chain length, consistent with the visual trends observed in the simulation snapshots. In this work, we introduced a para‐aniline group at the 3,6‐positions of the xanthene core to achieve bathochromic emission shift. By appending four extended alkyl chains to the aniline nitrogen atom, we realized substantially enhanced steric hindrance is realized, which more effectively suppresses molecular stacking and consequently augments fluorescence performance in aqueous media. This design strategy establishes a promising and generalizable platform for the development of high‐brightness NIR‐II fluorophores based on the xanthene scaffold.

### 2.4 In vivo NIR‐II fluorescence imaging of blood vessels

To evaluate the NIR‐II bioimaging capabilities of the four SCU‐OX derivatives, abdominal vascular imaging was performed on BALB/c mice using a 1300 nm long‐pass (LP) filter (Figure 3a). Among the four probes, SCU‐OX‐Me exhibited the lowest signal‐to‐noise ratio (SNR) and poorest spatial resolution. In contrast, SCU‐OX‐Hex demonstrated substantially superior imaging performance. Gaussian fitting of cross‐sectional fluorescence intensity profiles across abdominal vessels revealed full width at half maximum (FWHM) values of 719 and 892 μm, together with an SNR as high as 3.56—all metrics outperforming those of the other three compounds (Figure 3b). To further assess its depth‐dependent imaging capability, SCU‐OX‐Hex was evaluated in Eppendorf (Ep) tubes and capillaries immersed at varying depths in 1% Intralipid solution. Imaging was performed using a commercial NIR‐II in vivo imaging system equipped with an 808 nm excitation laser and multiple long‐pass filter configurations (Supporting Information S1: Figures S38 and S39). Collectively, these results establish that SCU‐OX‐Hex exhibits remarkable NIR‐II vascular imaging performance, thereby demonstrating the effectiveness of the design strategy.

**FIGURE 3:**
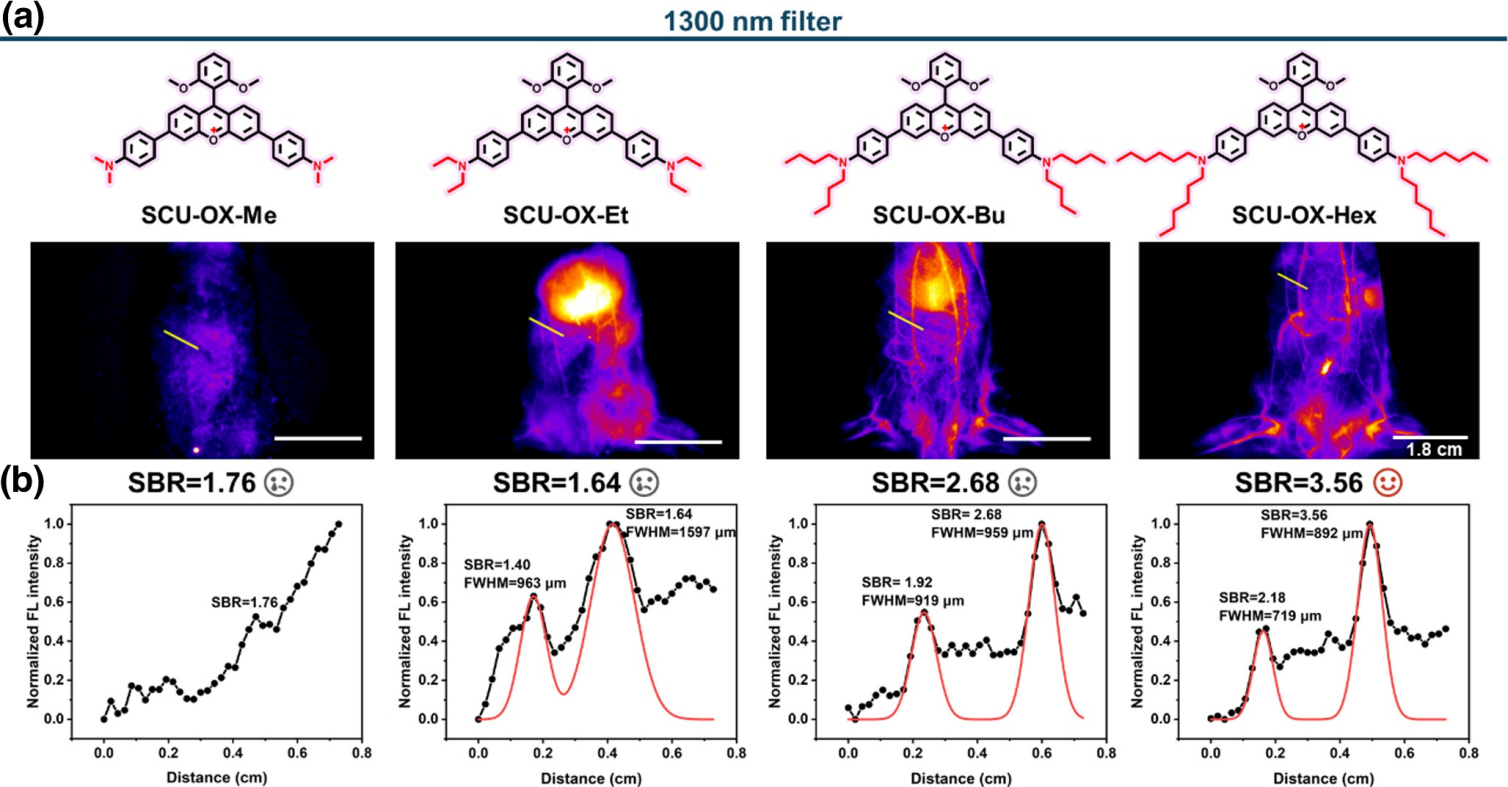
(a) Chemical structures of the four SCU‐OX derivatives and corresponding NIR‐II fluorescence imaging of abdominal vasculature in BALB/c mice. Images were acquired following intravenous injection of SCU‐OX‐Me, SCU‐OX‐Et, SCU‐OX‐Bu, and SCU‐OX‐Hex (1 mM in 10% DMSO, 2% Tween 80) using a 1300 nm long‐pass (LP) filter. (b) Cross‐sectional fluorescence intensity profiles along the yellow solid lines indicated in (a) fitted with Gaussian functions (red curves).

We next investigated the effect of emission wavelength selection on imaging quality. Following intravenous injection of SCU‐OX‐Hex, the shaved abdominal region of BALB/c mice was imaged through 1100 nm, 1200 nm, and 1300 nm long‐pass filters (Figure 4b). For larger abdominal vessels, the >1300 nm filter group yielded a signal‐to‐background ratio (SBR) of 1.94 and a measured vessel diameter of 925 μm, comparable to those obtained with the >1100 nm filter (SBR: 1.87; diameter: 1186 μm) and >1200 nm filter (SBR: 2.12; diameter: 1180 μm) (Figure 4a,d,f). For finer vessels, the more than 1300 nm filter group achieved an SBR of 1.44 and a diameter of 509 μm, notably higher than the >1100 nm group (SBR: 1.18; diameter: 582 μm) and >1200 nm group (SBR: 1.24; diameter: 543 μm) (Figure 4c,e,g). These findings demonstrate that SCU‐OX‐Hex affords superior SBR and imaging resolution at longer emission wavelength within the NIR‐II window. In summary, this study clearly demonstrates that SCU‐OX‐Hex exhibits excellent NIR‐II imaging performance, positioning it as a promising fluorescent probe for high‐contrast in vivo vascular visualization.

**FIGURE 4:**
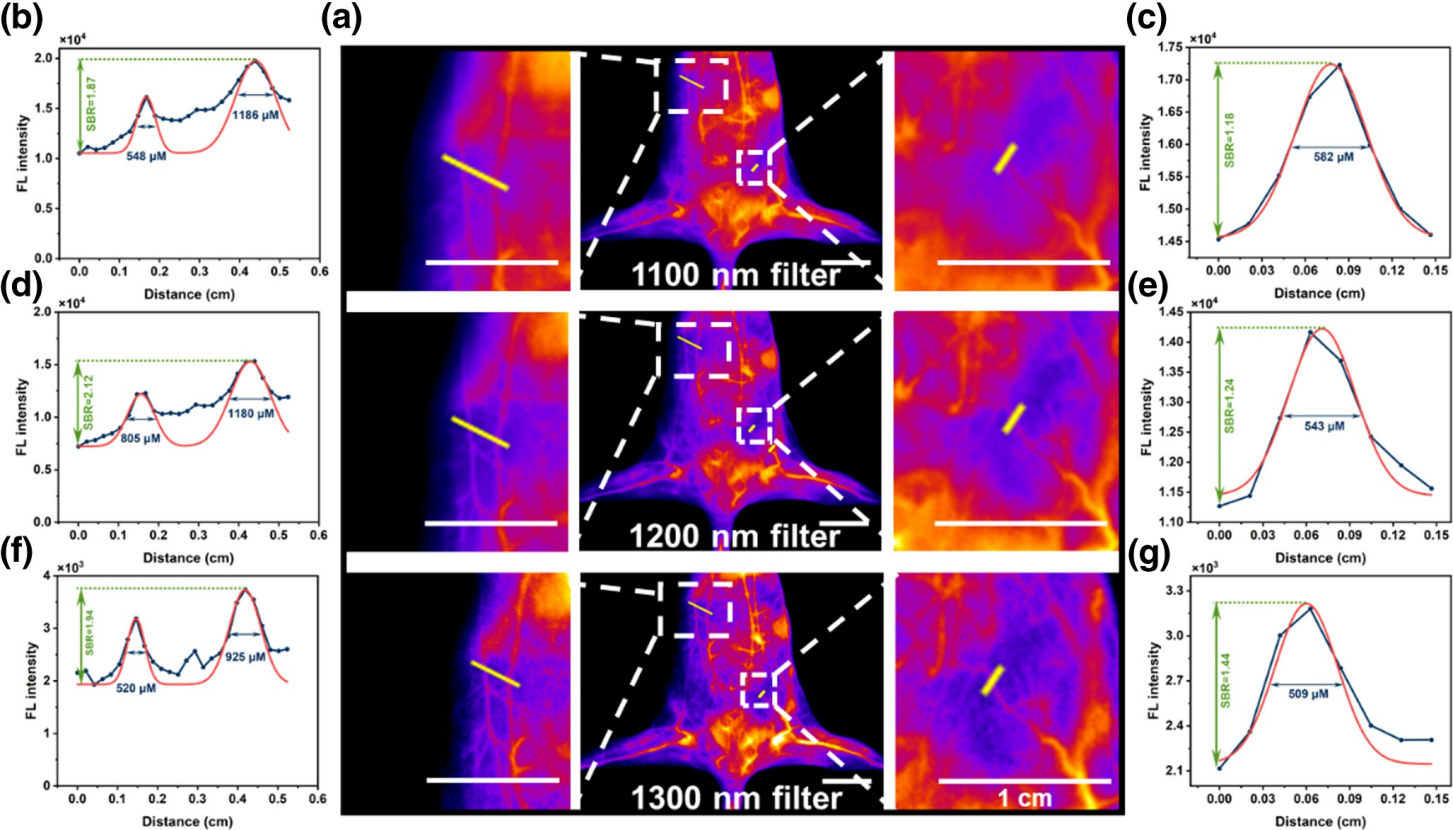
(a) NIR‐II fluorescence imaging of abdominal vasculature in BALB/c mice following intravenous injection of SCU‐OX‐Hex (1 mM in 10% DMSO/2% Tween 80). Images were acquired through 1100 nm, 1200 nm, and 1300 nm long‐pass (LP) filters, respectively. (b, c) Gaussian fitting curves (solid lines) of the cross‐sectional fluorescence intensity profiles along the left and right yellow dashed lines indicated in the 1100 nm LP filter image shown in (a). (d, e) Corresponding Gaussian fitting curves for the left and right yellow dashed lines in the 1200 nm LP filter image. (f, g) Corresponding Gaussian fitting curves for the left and right yellow dashed lines in the 1300 nm LP filter image. Imaging conditions: power density of 400 mW/cm^2^, exposure time of 100 ms.

### 2.5 In vivo NIR‐II fluorescence imaging of peritumoral vessels

Encouraged by its remarkable performance in abdominal vascular imaging, we next evaluated the utility of SCU‐OX‐Hex for visualizing tumor‐associated vasculature. Tumors, like healthy tissues, depend on the establishment of a functional blood supply to meet their metabolic demands for oxygen and nutrients.^[41]^ Consequently, real‐time monitoring of peritumoral vascular dynamics using fluorescent angiography probes can provide critical insights into tumor progression and microenvironmental remodeling. To this end, a murine orthotopic 4T1 breast tumor model was established in BALB/c mice via mammary pad injection and examined at three distinct stages (days 4, 10, and 17 post‐inoculation). Following intravenous administration of SCU‐OX‐Hex, real‐time fluorescence angiography was conducted using 1100 nm, 1200 nm, and 1300 nm long‐pass filters. Notably, images acquired through the 1300 nm filter exhibited substantially enhanced spatial resolution of peritumoral vessels compared with those obtained with shorter cutoff filters (Supporting Information S1: Figure S40). High‐resolution NIR‐II imaging through the 1300 nm filter revealed that, relative to normal abdominal vasculature (Figure 5a), the periphery of 4‐day tumors displayed markedly denser vascular networks (Figure 5b). Gaussian fitting of cross‐sectional intensity profiles demonstrated an increase in the FWHM.

(FWHM) of representative peritumoral vessels from 465 to 506 μm, accompanied by elevated fluorescence signal intensity (Figure 5e,f). Longitudinal assessment at 10 and 17 days post‐inoculation revealed progressive vascular remodeling (Figure 5c,d). At day 10, peritumoral vessels exhibited further FWHM expansion from 506 to 968 μm (Figure 5g), along with the appearance of neovessels linking to the tumor mass. By day 17, the FWHM of peritumoral vessels had further extended to 1223 μm (Figure 5h). Although the vascular fluorescence intensity remained relatively stable throughout tumor progression, clear evidence of vascular invasion into adjacent healthy tissue was observed. These morphological changes—including vessel dilation and neovascularization—suggest enhanced nutrient and oxygen acquisition to support rapid tumor growth. In summary, SCU‐OX‐Hex enables high‐resolution real‐time NIR‐II angiography of the peritumoral vasculature throughout the entire course of tumor development. This capability permits accurate non‐invasive assessment of vascular expansion and invasive remodeling, positioning SCU‐OX‐Hex as a powerful molecular imaging tool for investigating tumor angiogenesis and evaluating therapeutic responses.

**FIGURE 5:**
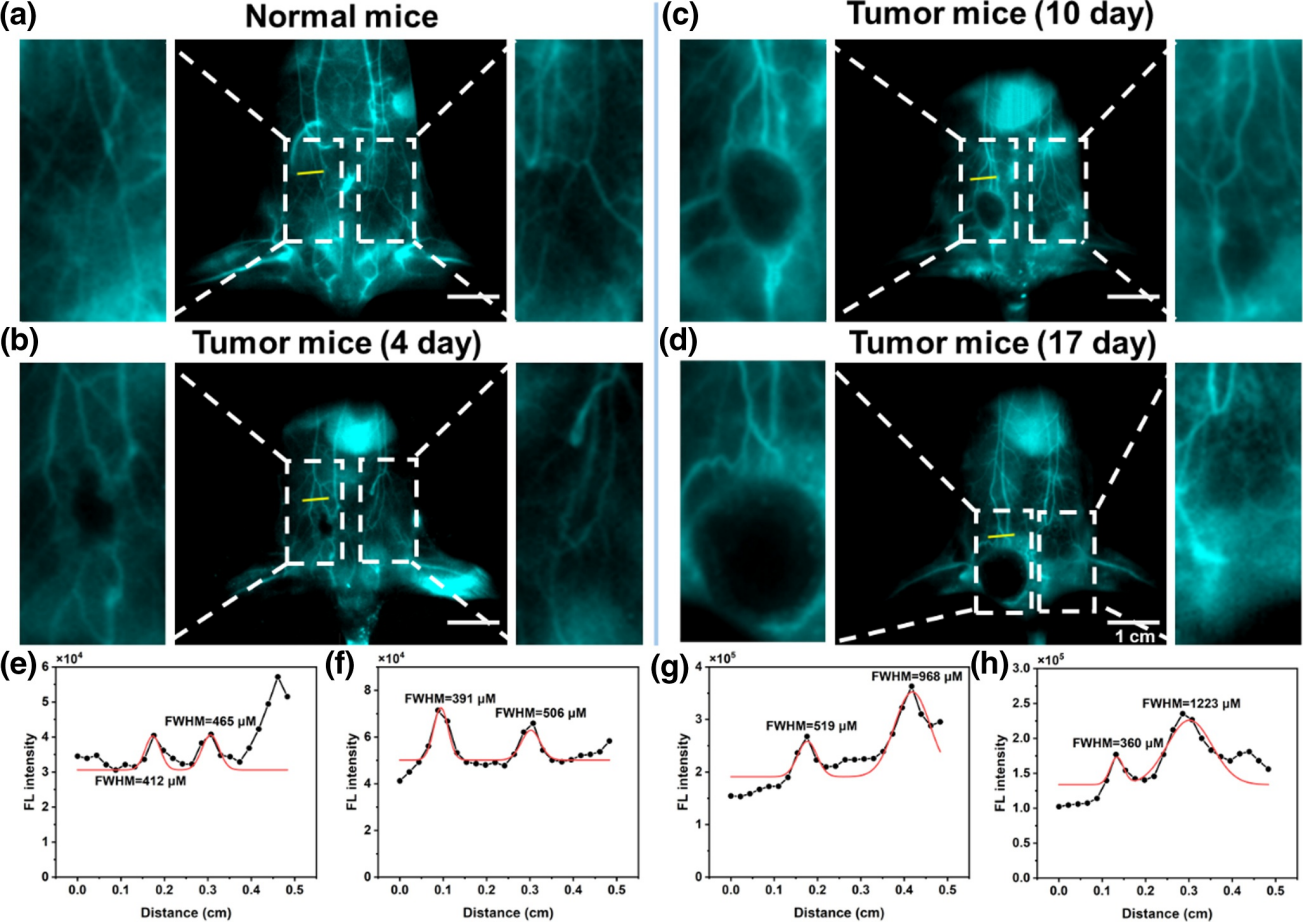
In vivo NIR‐II fluorescence imaging of peritumoral vascular dynamics during tumor progression using SCU‐OX‐Hex. Following intravenous injection of SCU‐OX‐Hex (1 mM in 10% DMSO/2% Tween 80), images were acquired through a 1300 nm long‐pass filter. (a) Whole‐body NIR‐II image of abdominal vasculature in a healthy BALB/c mouse (left) and corresponding magnified view of the region delineated by the white box (right). (b–d) Whole‐body NIR‐II images of peritumoral vasculature at 4 days (b), 10 days (c), and 17 days (d) post subcutaneous inoculation of 4T1 tumors, with magnified views of the white‐boxed regions shown adjacent to each whole‐body image. (e–h) Cross‐sectional fluorescence intensity profiles (dotted lines) extracted along the yellow dashed lines indicated in (a–d), overlaid with corresponding Gaussian fitting curves (solid red lines): (e) healthy control, (f) day 4, (g) day 10, and (h) day 17 post‐inoculation. Imaging conditions: power density of 400 mW/cm^2^, exposure time of 100 ms.

### 2.6 In vivo NIR‐II fluorescence imaging of gastrointestinal tract

To evaluate the utility of SCU‐OX‐Hex for gastrointestinal (GI) tract imaging, the probe was administered via oral gavage (100 μM, 200 μL) and NIR‐II fluorescence imaging was performed in real time using a 1300 nm long‐pass filter (Figure 6a,b). Fluorescence signal in the stomach was detectable immediately following administration. At 1 h post‐administration, the small intestine was clearly delineated with high signal‐to‐noise ratio, enabling detailed visualization of intestinal morphology. Cross‐sectional fluorescence intensity profiling along the small intestine yielded FWHM values of 3.36 mm, 1.64 mm, and 3.65 mm for distinct intestinal segments, reflecting the high spatial resolution afforded by SCU‐OX‐Hex (Figure 6c). To assess the clearance profile, fecal samples were collected at 8 and 24 h post‐administration and subjected to fluorescence analysis (Supporting Information S1: Figure S41). At 8 h, fecal specimens exhibited strong NIR‐II fluorescence; by 24 h, the signal intensity had diminished to less than one‐third of the 8 h level.

**FIGURE 6:**
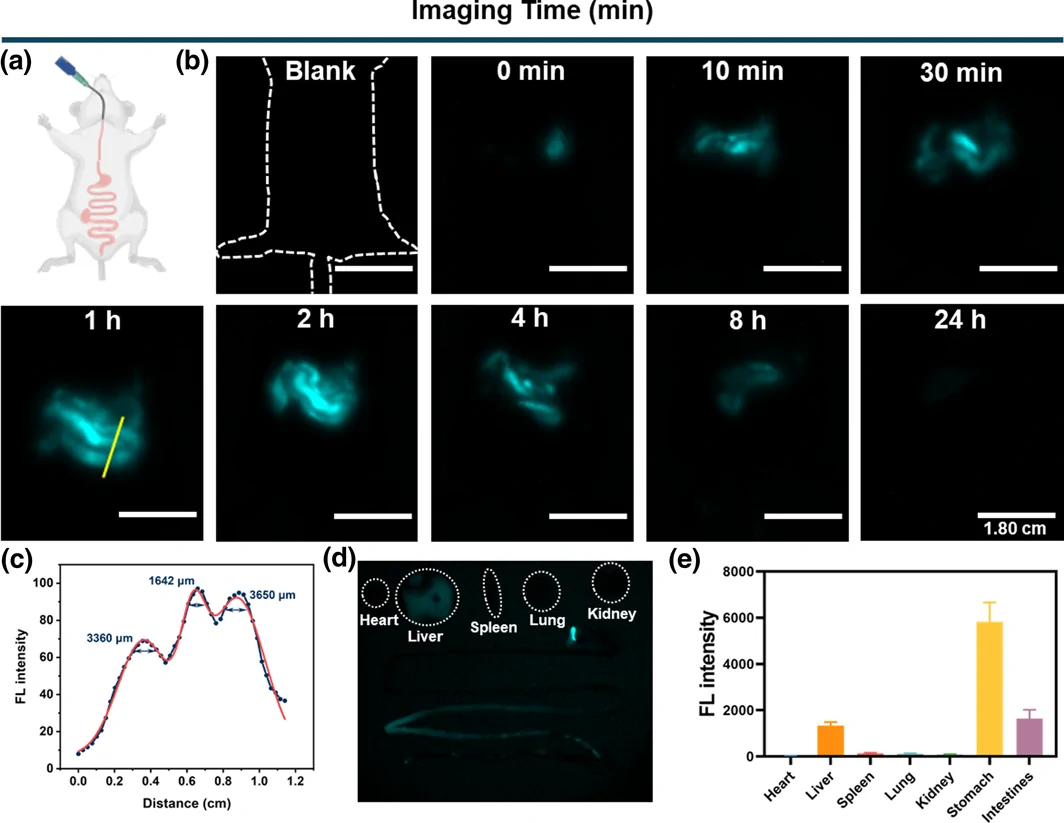
Biodistribution and gastrointestinal imaging performance of SCU‐OX‐Hex following oral administration. SCU‐OX‐Hex (100 μM in 10% DMSO, 2% Tween 80) was administered via oral gavage. (a) Schematic illustration of the oral gavage procedure in mice. (b) Time‐course in vivo NIR‐II fluorescence imaging of the abdominal region acquired through a 1300 nm long‐pass filter, depicting dynamic transit of SCU‐OX‐Hex through the gastrointestinal tract. (c) Cross‐sectional fluorescence intensity profile (dotted line) extracted along the yellow solid line indicated in the 1 h post‐administration image shown in (b), overlaid with the corresponding Gaussian fitting curve (solid red line). (d) Ex vivo NIR‐II fluorescence imaging of major organs harvested at 24 h post‐administration. (e) Quantitative analysis of mean fluorescence intensity for each organ depicted in (d). Imaging conditions: power density of 400 mW/cm^2^, exposure time of 100 ms.

Consistent with this excretion pattern, no appreciable NIR‐II fluorescence signal remained in vivo at 24 h, indicating efficient systemic clearance of the probe. To corroborate these imaging findings, mice were euthanized and dissected at 24 h post‐administration (Figure 6d and Supporting Information S1: Figure S42). With the exception of trace residual signal detected in the stomach, no significant fluorescence accumulation was observed in any major organs (Figure 6e). These results confirm that SCU‐OX‐Hex remains predominantly confined to the GI tract following oral administration and was effectively eliminated via the fecal route. Collectively, this study establishes SCU‐OX‐Hex as a promising NIR‐II fluorescent probe for real‐time high‐resolution imaging of GI tract structure and motility, with favorable clearance characteristics.

### 2.7 In vivo NIR‐II fluorescence imaging of metastatic popliteal lymph nodes

Current intraoperative detection of metastatic lymph nodes remains heavily dependent on manual palpation, surgeon experience, preoperative imaging modalities (e.g., computed tomography, magnetic resonance imaging), and intraoperative frozen section analysis. However, palpation and experiential assessment are inherently subjective and offer limited accuracy, whereas frozen section biopsy is time‐consuming and susceptible to sampling error.^[42]^ SCU‐OX‐Hex, as an emerging NIR‐II fluorescent probe, has demonstrated substantial potential for high‐contrast bioimaging. This study aims to leverage SCU‐OX‐Hex for precise intraoperative identification of tumor‐draining metastatic lymph nodes, thereby enabling complete resection of metastatic or suspicious nodes and ultimately improving postoperative quality of life.

A unilateral (left paw) mouse model of 4T1 breast cancer was established in BALB/c mice by plantar inoculation (Figure 7a). Commercial indocyanine green (ICG, 1 mM in 10% DMSO, 2% Tween 80) and SCU‐OX‐Hex (1 mM in 10% DMSO, 2% Tween 80) were administered via tail vein injection, and NIR‐II fluorescence imaging was performed to dynamically monitor signal accumulation in the popliteal and sciatic lymph nodes at multiple time points. ICG successfully labeled bilateral sciatic lymph nodes from 60 to 180 min post‐injection; however, no fluorescence signal was detectable in the popliteal lymph nodes throughout the entire imaging period (Figure 7b). Notably, SCU‐OX‐Hex clearly delineated both sciatic and popliteal lymph nodes at 120 min post‐injection, maintaining a high signal‐to‐noise ratio up to 180 min. The SBR on the tumor‐bearing side was also markedly elevated relative to the contralateral side (1.93, 1.31, Figure 7h). Overall, SCU‐OX‐Hex afforded superior SBR and enhanced imaging resolution for metastatic lymph node identification compared with ICG.

**FIGURE 7:**
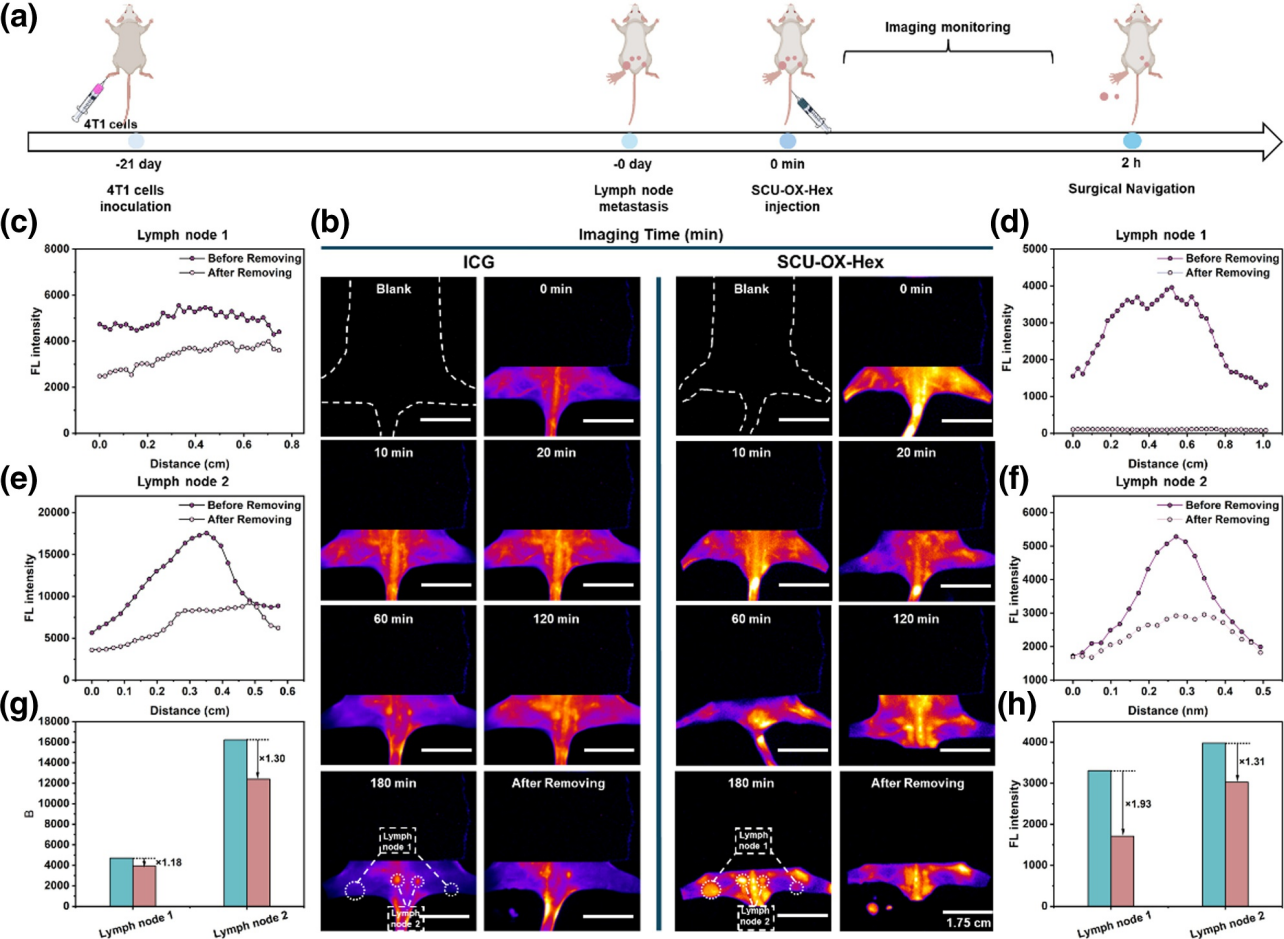
NIR‐II fluorescence imaging of metastatic lymph nodes using ICG and SCU‐OX‐Hex. SCU‐OX‐Hex or ICG (1 mM in 10% DMSO, 2% Tween 80) was intravenously injected into BALB/c mice bearing lymph node metastases, and images were acquired through a 1200 nm long‐pass filter. (a) Schematic illustration of the tumor inoculation and subsequent surgical resection procedure. (b) Time‐course NIR‐II fluorescence imaging of the sciatic and popliteal regions following intravenous administration of ICG (left) and SCU‐OX‐Hex (right). (c), (d) Quantitative analysis of fluorescence intensity in the popliteal lymph node (Lymph Node 1) before and after surgical resection, corresponding to ICG (c) and SCU‐OX‐Hex (d) imaging. (e), (f) Quantitative analysis of fluorescence intensity in the sciatic lymph node (Lymph Node 2) before and after surgical resection, corresponding to ICG (e) and SCU‐OX‐Hex (f) imaging. (g), (h) Comparative analysis of pre‐surgical fluorescence intensity between popliteal and sciatic lymph nodes visualized by NIR‐II imaging with ICG (g) and SCU‐OX‐Hex (h). Imaging conditions: power density of 400 mW/cm^2^, exposure time of 100 ms.

The preferential accumulation of SCU‐OX‐Hex in metastatic nodes is attributed to the enhanced permeability and retention (EPR) effect resulting from tumor‐induced lymphangiogenesis and vascular remodeling. In fluorescence‐guided lymph node resection experiments, ICG provided insufficient resolution for popliteal lymph nodes and failed to offer clear intraoperative navigation (Figure 7c, Video S3 and S4). Conversely, SCU‐OX‐Hex enabled high‐quality imaging and successfully guided complete resection of popliteal lymph nodes (Figure 7d, Video S1 and S2). For sciatic lymph nodes, both probes afforded relatively high imaging contrast sufficient to assist surgical resection (Figure 7e,f). To further validate the pathological basis for enhanced probe accumulation, H&E staining was performed on resected lymph nodes (Supporting Information S1: Figure S43). Tumor‐invaded lymph nodes exhibited pronounced neovascularization and thickened vascular walls, alterations that facilitate increased lymphatic drainage and probe extravasation—finding consistent with the observed NIR‐II fluorescence imaging results. Dark toxicity evaluation in NIH‐3T3 fibroblasts demonstrated that SCU‐OX‐Hex maintained cell viability above 70% even at concentrations up to 40 μM, indicating favorable biocompatibility under the tested conditions (Supporting Information S1: Figure S44). In summary, SCU‐OX‐Hex, leveraging its superior NIR‐II imaging performance, enables high‐quality, high‐resolution fluorescence visualization of metastatic sciatic and popliteal lymph nodes and permits precise intraoperative identification and guidance for complete resection. These findings establish SCU‐OX‐Hex as a promising candidate for clinical translation in image‐guided oncologic surgery.

## 3 CONCLUSION

In this study, we employed O‐xanthene as the electron acceptor and aniline as the electron donor, and demonstrated that extending the N‐alkyl chain on the aniline moiety effectively enhances both fluorescence QY and molecular brightness. Guided by this design principle, we synthesized a series of NIR‐II fluorescent probes—SCU‐OX‐Me, SCU‐OX‐Et, SCU‐OX‐Bu, and SCU‐OX‐Hex. Among them, SCU‐OX‐Hex exhibited exceptional photophysical properties, including a high molar extinction coefficient (1.71 × 10^5^ M^−1^ cm^−1^), a NIR‐II fluorescence QY of 0.57%, and a molecular brightness of 974.7 M^−1^ cm^−1^. Moreover, this fluorophore demonstrated excellent photostability and favorable biocompatibility. In vivo NIR‐II imaging studies revealed that SCU‐OX‐Hex enables high‐quality visualization of the abdominal vasculature and the gastrointestinal tract in mice. It also permits dynamic monitoring of tumor vascular remodeling and invasive progression across distinct anatomical sites. In a lymph node metastasis model, SCU‐OX‐Hex afforded markedly superior imaging contrast and spatial resolution for metastatic sciatic and popliteal lymph nodes compared with the clinically approved dye indocyanine green (ICG), facilitating precise intraoperative identification and complete surgical resection. These results collectively underscore the exceptional NIR‐II imaging capability of SCU‐OX‐Hex. In summary, this work not only establishes a viable molecular engineering strategy for the development of NIR‐II probes with high fluorescence QY and brightness, but also provides a versatile small‐molecule scaffold based on O‐xanthene for the rational design of next‐generation high‐performance NIR‐II fluorescent probes.

## CONFLICT OF INTEREST STATEMENT

The authors declare no conflicts of interest.

## ETHICS STATEMENT

All animal studies were approved by the Sichuan University Animal Charity Protection and Treatment Committee and performed in accordance with humane care and use of research animals under a protocol (Ethics No. 20240226173).

## Supporting information

Supporting Information S1

Video S1: NIR‐II fluorescence imaging and surgically navigated resection of the popliteal and sciatic lymph nodes on the tumor side 21 days after plantar inoculation with 4T1 tumor cells, following tail‐vein administration of SCU‐OX‐Hex.

Video S2: NIR‐II fluorescence imaging and surgically navigated resection of the popliteal lymph node on the normal side following tail‐vein administration of SCU‐OX‐Hex.

Video S3: NIR‐II fluorescence imaging and surgically navigated resection of the popliteal and sciatic lymph nodes on the tumor side 21 days after plantar inoculation with 4T1 tumor cells, following tail‐vein administration of ICG.

Video S4: NIR‐II fluorescence imaging and surgically navigated resection of the popliteal lymph node on the normal side following tail‐vein administration of ICG.

## Data Availability

The data that supports the findings of this study are available in the supplementary material of this article.
