# Supplementary material for: Alkyl chain elongation engineering: Unlocking high‐brightness NIR‐II O‐xanthene dyes for in vivo bioimaging
Source: Smart Mol. 2026 Oct 29:e70117. Online ahead of print. doi: 10.1002/smo2.70117 (PMC13719896; doi:10.1002/smo2.70117)
Supplement: Supplementary file 1 [file SMO2-9999-0-s002.pdf]

Alkyl Chain Elongation Engineering: Unlocking High-brightness NIR-II O-Xanthene Dyes for In Vivo Bioimaging

Yu Zhao [a], Hong Zhang [a], Ruirui Zhang [a], Wenli Xia [a], Zhouyu Wang [b], Jie Chen [a], Xiaoqi Yu *[b], and Kun Li *[a]

[a]Key Laboratory of Green Chemistry and Technology, Ministry of Education, College of Chemistry, Sichuan University, Chengdu, 610064, P. R. China. E-mail: kli@scu.edu.cn (K. Li).

[b] Sichuan Engineering Research Center of Molecular Targeted Diagnostic & Therapeutic Drugs, Department of Chemistry, Xihua University, Chengdu 610039, P. R. China. Email: yuxq@xhu.edu.cn

SUPPLEMENTARY METHODS/SECTION

1. Materials

All chemicals and reagents were commercially available and used as received without further purification. The intermediate compounds were synthesized according to the previously reported procedures. Cell Counting Kit-8 (CCK-8) were purchased from Sigma-Aldrich and used as received. For cell culture, Roswell Park Memorial Institute (RPMI) 1640, Dulbecco's Modified Eagle Medium (DMEM) fetal bovine serum (FBS), penicillin–streptomycin solution was purchased from Beyotime.

2. Instruments

^{1}H NMR and ^{13}C NMR spectra were measured on Bruker AM400 NMR spectrometer. Mass spectra were recorded on Finnigan LCQDECA mass spectrometer. UV absorption spectra were measured on Horiba Duetta. NIR-II fluorescence spectra were measured on a Fluorolog-3 (HORIBA) spectrofluorometer. In vivo imaging was obtained using a NIR-II Live Imaging System with NIR-Ⅱ-ST (DIGI-UNITED BIOTECHNOLOGY).

Table S1. The summary of photophysical parameters of NIR-II fluorophores of recently reported.

Dyes	λ_{abs} (nm)	λ_{em} (nm)	ε (M^{-1} cm^{-1})	Φ (%)	Brightness (M^{-1} cm^{-1})	References
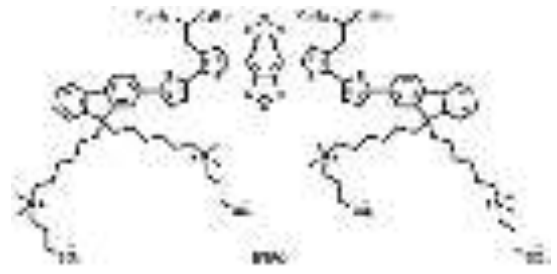	**870**	**1098**	**2.4×10^{3}**	**0.65**	**15.6**	***Adv. Funct. Mater.*** **2022,32, 2208463**
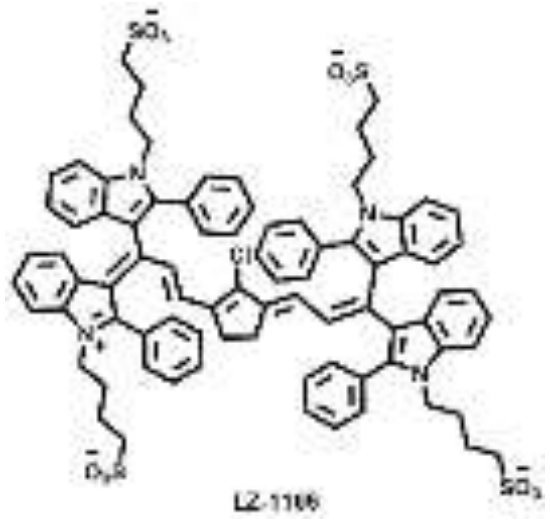 LZ-1105	**1041**	**1105**	**1.01×10^{5}**	**0.03**	**30**	***Nat. Commun. 2020,*** **11, 3102**
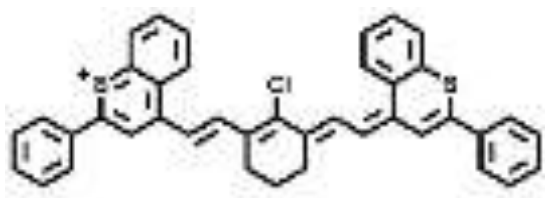 IR26	**1084**	**1144**	**1.3×10^{5}**	**0.05**	**65**	***Nat. Commun. 2019, 10, 1058***
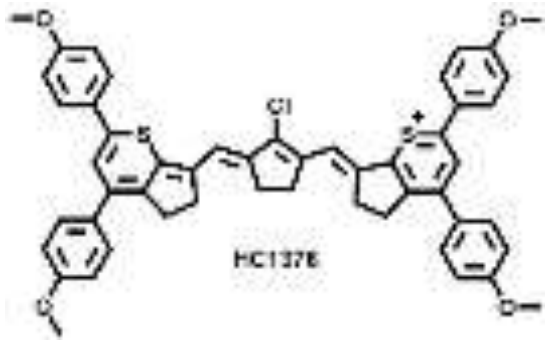 HC1376	**1312**	**1376**	**9.18×10^{4}**	**0.011**	**10.1**	***Angew. Chem. Int. Ed. 2022, 61, e202117436***
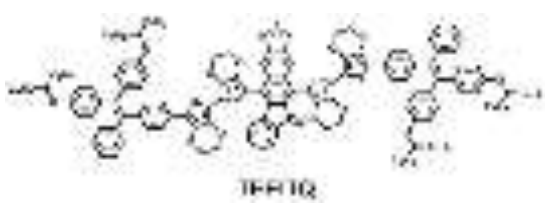	**799**	**1080**	**1.68×10^{4}**	**0.24**	**40.32**	***Angew. Chem. Int. Ed. 2025, 64, e202413219***
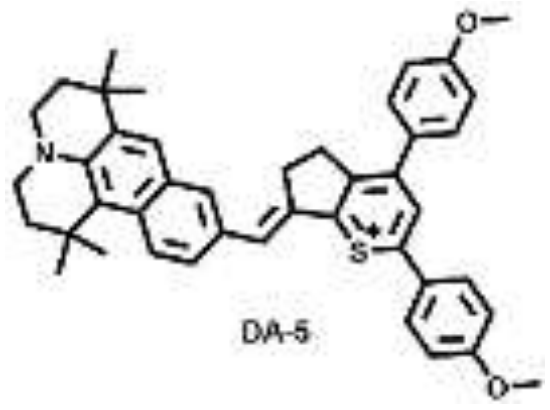 DA-5	**847**	**1088**	**4.16×10^{4}**	**0.77**	**321**	***J. Med. Chem. 2025, 68, 3782−3794***
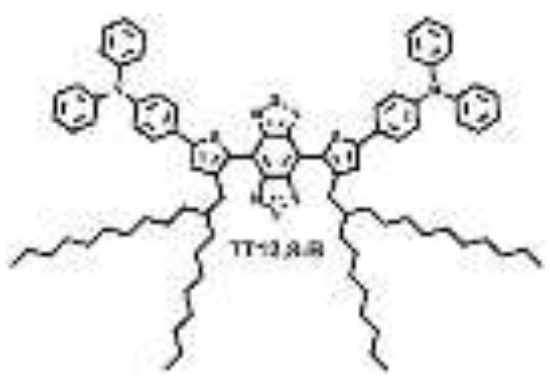	**700**	**1010**	**1.96×10^{4}**	**0.69 (NPs)**	**70**	***ACS Nano 2025, 10.1021/acsnano.5c16882***
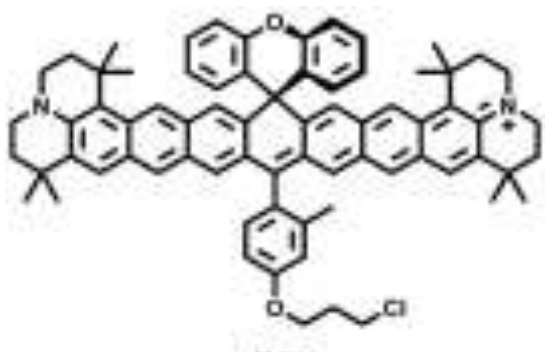 EC7	**1204**	**1290**	**3.91×10^{5}**	**0.053**	**207**	***J. Am. Chem. Soc. 2023, 145, 22, 12013–12022***

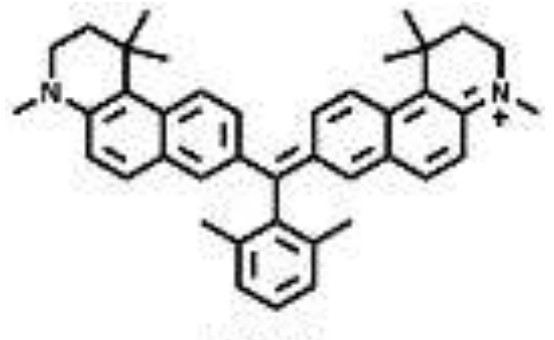 EA5_c3	877	979	1.38×10^5	0.05	69.00	*Adv. Mater.* *2025, 37, 2411515*
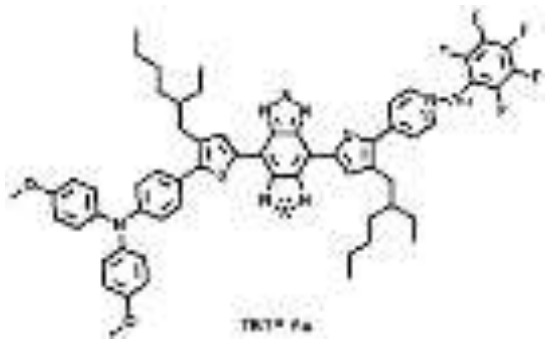	809	1080	2.58×10^4	0.2	51.6	*Adv. Mater.* *2023, 35, 2306616*
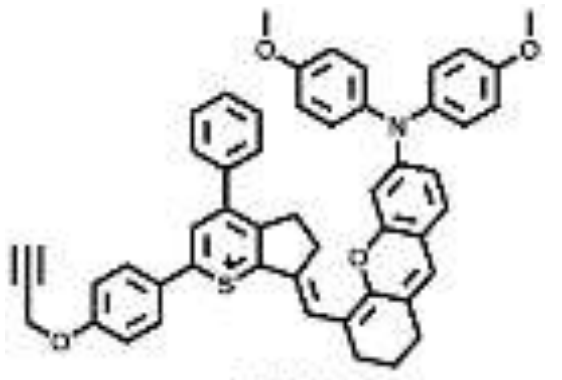 HTPA	950	1115	2.23×10^4	0.19	42.37	*Adv. Mater.* *2025, 2420006*
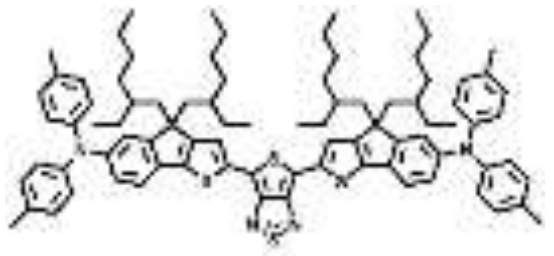	794	1060	3.1×10^4	2.52	783.7	*Adv. Mater.* *2025, e10386*
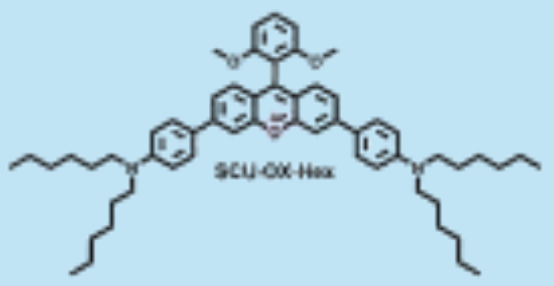 SCU-OX-Hex	812	984	1.71×10^5	0.57	974.7	*This work*

SYNTHESIS AND CHARACTERISTICS

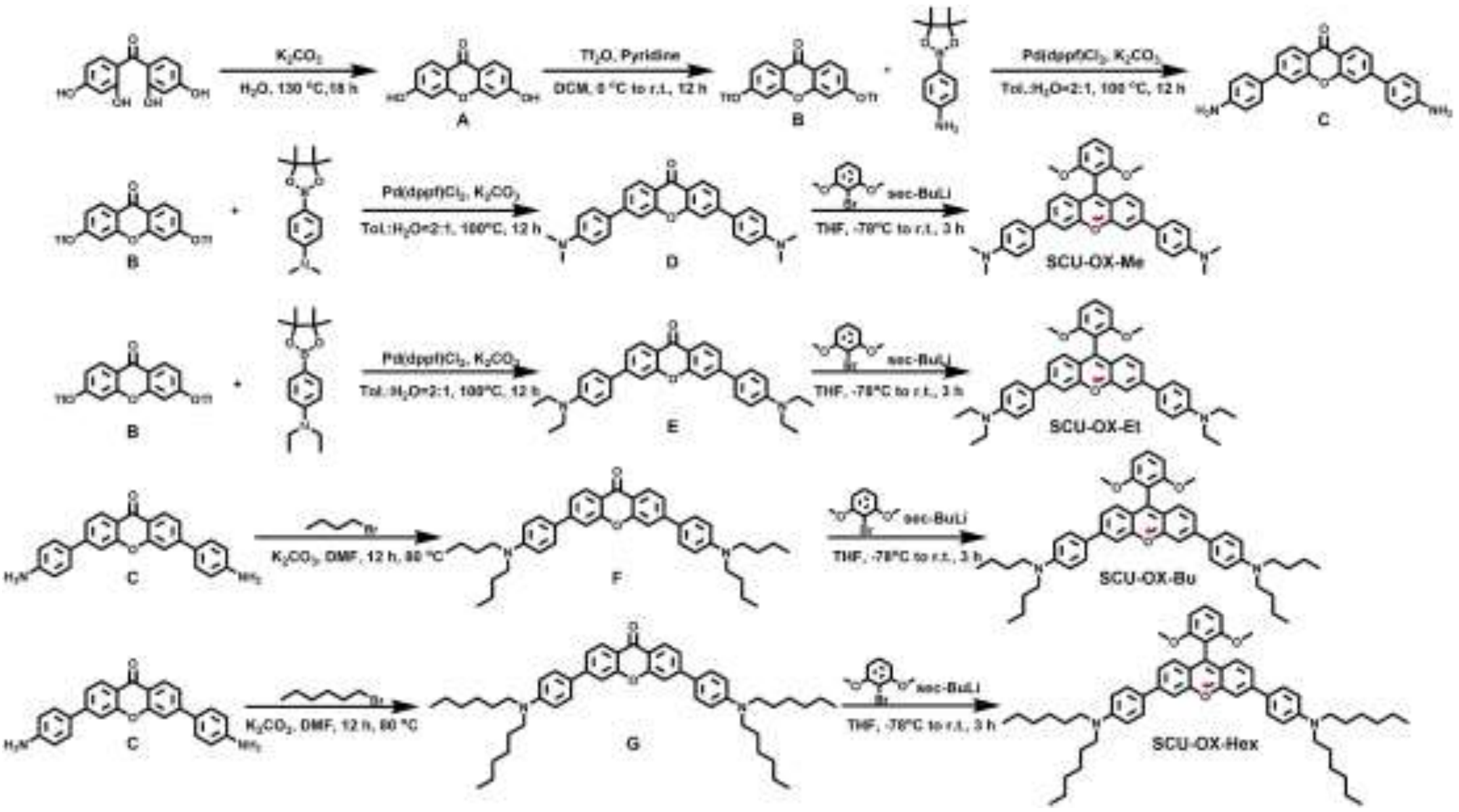

Scheme S1. The structure and synthetic route of SCU-OX-Me, SCU-OX-Et, SCU-OX-Bu, SCU-OX-Hex.

Synthetic route of Compound A: To a solution of 2,2',4,4'-Tetrahydroxybenzophenone (10 g, 40.6 mmol) in 100 mL of water, K_2CO_3 (560 mg, 0.1 mmol) was added. Then the reaction mixture was stirred for 18 h at 150 °C. Upon cooling room temperature, filter under vacuum, and wash the residue three times with water. The crude product obtained is unpurified and ready for the next step.

Synthetic route of Compound B: The compound A (8 g, 35 mmol) was dissolved in anhydrous dichloromethane, and pyridine (8.3 g 3 mmol) was slowly added at 0 °C. The mixture was stirred for 30 min. Subsequently, a solution of trifluoromethanesulfonic anhydride (21.7 g, 2.2 mmol) in dichloromethane was added dropwise via a pressure-equalizing dropping funnel. After completion of the addition, the reaction mixture was stirred at room temperature for 12 h. The reaction was quenched with 2 M HCl to consume the excess trifluoromethanesulfonic anhydride. The mixture was extracted with dichloromethane, and the combined organic layers were washed successively with 2 M HCl and saturated NaCl solution (three times each) to remove residual pyridine. After removing the solvent, the crude product was purified by column chromatography on silica gel using dichloromethane as eluent to give the final product as white solid (15 g, yield: 86.9 %). ^{1}H NMR (400 MHz, $CDCl_3$) δ 8.43 (d, *J* = 8.8 Hz, 2H), 7.49 (d, *J* = 2.1 Hz, 2H), 7.35 (dd, *J* = 8.8, 2.1 Hz, 2H). ^{13}C NMR (101 MHz, $CDCl_3$) δ 174.50 (s), 156.54 (s), 153.31 (s), 129.59 (s), 121.34 (s), 120.24 (s), 118.16 (s), 117.05 (s), 111.42 (s). ^{19}F NMR(376 MHz,CDC:) -72.55.

Synthetic route of Compound C: A solution of compound B (5 g, 10.2 mmol), 4-(4,4,5,5-Tetramethyl-1,3,2-dioxaborolan-2-yl) aniline (4.69 g, 21.42 mmol), Pd(dppf)Cl_2 (75 mg,

0.102 mmol), and K_2CO_3 (4.23 g, 30.6 mmol) was refluxed under nitrogen in the dry solvent of toluene and water (100 mL ($V_{Tol.}/V_{water}$=2:1) at 110 °C overnight. After cooling to room temperature, the crude product was purified by column chromatography on silica gel to give the final product as a yellow solid (3 g, yield: 78.9 %). ^{1}H NMR (400 MHz, DMSO) δ 8.12 (d, *J* = 8.4 Hz, 2H), 7.72 (d, *J* = 1.3 Hz, 2H), 7.68 (dd, *J* = 8.4, 1.4 Hz, 2H), 7.59 (d, *J* = 8.6 Hz, 4H), 6.68 (d, *J* = 8.5 Hz, 4H), 5.57 (s, 4H). ^{13}C NMR (101 MHz, DMSO) δ 175.37 (s), 156.86 (s), 150.54 (s), 147.84 (s), 128.52 (s), 126.79 (s), 125.05 (s), 121.88 (s), 119.19 (s), 114.59 (s), 113.15 (s).

Synthetic route of Compound D: A solution of compound B (500 mg, 1.02 mmol), N,N-Dimethyl-4-(4,4,5,5-tetramethyl-1,3,2-dioxaborolan-2-yl)aniline (529 mg, 2.14 mmol), Pd(dppf)Cl_2 (75 mg, 0.102 mmol), and K_2CO_3 (423 mg, 3.06 mmol) was refluxed under nitrogen in the dry solvent of toluene and water (20 mL ($V_{Tol.}/V_{water}$=2:1) at 110 °C overnight. After cooling to room temperature, the crude product was purified by column chromatography on silica gel to give the final product as a yellow solid (350 mg, yield: 79.4 %). ^{1}H NMR (400 MHz, cdcl$_3$) δ 8.25 (d, *J* = 8.3 Hz, 2H), 7.58 (dd, *J* = 15.1, 8.5 Hz, 8H), 6.79 (d, *J* = 8.3 Hz, 4H), 2.99 (s, 12H). ^{13}C NMR (101 MHz, cdcl$_3$) δ 177.13 (s), 156.90 (s), 147.80 (s), 128.06 (s), 126.78 (s), 121.99 (s), 119.50 (s), 113.84 (s), 112.64 (s), 40.38 (s).

Synthetic route of Compound SCU-OX-Me: To a solution of 1-Bromo-2,6-dimethoxybenzene (499 mg, 2.3 mmol) in 5 mL of dry THF, 1.77 mL of sec-BuLi (1.3 M in hexane, 2.3 mmol) was added dropwise at -78 °C. Then the reaction mixture was stirred for 1 h at -78 °C. After that, the compound **D** (100 mg, 0.23 mmol) was added to one portion. The mixture was stirred for 12 h at room temperature. Subsequently, 2 M HCl was added and stirred for 15 min, and poured into water and extracted with DCM (20 mL) three times. The organic phase was dried with Na_2SO_4 and evaporated in vacuo. The crude product was purified by column chromatography on silica gel to give the final product as a green solid (100 mg, yield: 78.1 %). ^{1}H NMR (400 MHz, $CDCl_3$) δ 8.13 (s, 2H), 7.98 (d, *J* = 9.7 Hz, 2H), 7.90 (d, *J* = 8.3 Hz, 4H), 7.68 (dd, *J* = 16.8, 8.3 Hz, 3H), 6.90 (t, *J* = 7.0 Hz, 6H), 3.73 (s, 6H), 3.19 (s, 12H). ^{13}C NMR (101 MHz, $CDCl_3$) δ 165.11 – 164.91 (m), 161.47 (d, *J* = 7.9 Hz), 157.85 (s), 156.92 (s), 137.51 (s), 134.65 (s), 133.68 (s), 129.52 (s), 127.09 (s), 126.44 (s), 116.69 (s), 114.84 (s), 112.13 – 111.93 (m), 108.27 (s), 59.90 (s), 43.91 (s). HRMS (ESI) calculated $C_{37}H_{35}N_2O_3^+$ (M^+) 555.2643, found 555.2648.

Synthetic route of Compound E: A solution of compound B (500 mg, 1.02 mmol), N,N-Diethyl-4-(4,4,5,5-tetramethyl-1,3,2-dioxaborolan-2-yl)aniline (589 mg, 2.14 mmol), Pd(dppf)Cl_2 (75 mg, 0.102 mmol), and K_2CO_3 (423 mg, 3.06 mmol) was refluxed under nitrogen in the dry solvent of toluene and water (20 mL ($V_{Tol.}/V_{water}$=2:1) at 110 °C overnight. After cooling to room temperature, the crude product was purified by column chromatography on silica gel to give the final product as a yellow solid (400 mg, yield:

80.3 %). ^{1}H NMR (400 MHz, $CDCl_3$) δ 8.32 (d, *J* = 8.3 Hz, 2H), 7.61 (dd, *J* = 14.3, 8.5 Hz, 8H), 6.78 (d, *J* = 8.3 Hz, 4H), 3.43 (q, *J* = 6.9 Hz, 8H), 1.22 (t, *J* = 6.9 Hz, 12H). ^{13}C NMR (101 MHz, $CDCl_3$) δ 156.91 (s), 148.13 (s), 147.62 (s), 128.32 (s), 126.90 (s), 125.39 (s), 121.71 (s), 119.61 (s), 113.55 (s), 111.72 (s), 44.43 (s), 12.62 (s).

Synthetic route of Compound SCU-OX-Et: To a solution of 1-Bromo-2,6-dimethoxybenzene (440 mg, 2.0 mmol) in 5 mL of dry THF, 1.54 mL of sec-BuLi (1.3 M in hexane, 2.0 mmol) was added dropwise at -78 °C. Then the reaction mixture was stirred for 1 h at -78 °C. After that, the compound **E** (100 mg, 0.20 mmol) was added to one portion. The mixture was stirred for 12 h at room temperature. Subsequently, 2 M HCl was added and stirred for 15 min, and poured into water and extracted with DCM (20 mL) three times. The organic phase was dried with Na_2SO_4 and evaporated in vacuo. The crude product was purified by column chromatography on silica gel to give the final product as a green solid (90 mg, yield: 72.0 %). ^{1}H NMR (400 MHz, $CDCl_3$) δ 8.01 (s, 2H), 7.88 (d, *J* = 9.0 Hz, 2H), 7.82 (d, *J* = 9.0 Hz, 4H), 7.62 (td, *J* = 8.5, 3.4 Hz, 1H), 7.56 (d, *J* = 8.9 Hz, 2H), 6.87 – 6.75 (m, 6H), 3.67 (s, 6H), 3.55 – 3.42 (m, 8H), 1.24 (t, *J* = 7.0 Hz, 12H). ^{13}C NMR (101 MHz, $cdcl_3$) δ 160.01 (s), 157.45 (s), 153.44 (s), 150.80 (s), 133.44 (s), 130.59 (s), 130.01 (s), 125.29 (s), 122.40 (d, *J* = 19.0 Hz), 112.32 (s), 110.44 (s), 108.05 (s), 104.28 (s), 55.99 (s), 44.81 (s), 12.54 (s). HRMS (ESI) calculated $C_{41}H_{43}N_2O_3^+$ (M^+) 611.3269, found 611.3269.

Synthetic route of Compound F: The Compound C (1g, 2.6 mmol) was dissolved in 20 mL of DMF, followed by the addition of potassium carbonate (1.08 g, 7.8 mmol) and bromobutane (3.56 g, 26 mmol). The reaction mixture was stirred at 100 °C for 12 h. After cooling to room temperature, the mixture was diluted with a large amount of water and extracted with ethyl acetate. The organic phase was dried with Na_2SO_4 and evaporated in vacuo. The crude product was purified by column chromatography on silica gel to give the final product as a yellow solid (1 g, yield: 62.9 %). ^{1}H NMR (400 MHz, $cdcl_3$) δ 8.32 (d, *J* = 8.3 Hz, 2H), 7.60 (dd, *J* = 12.0, 9.1 Hz, 8H), 6.74 (d, *J* = 8.7 Hz, 4H), 3.41 – 3.25 (m, 8H), 1.62 (dt, *J* = 15.0, 7.7 Hz, 8H), 1.39 (dq, *J* = 14.6, 7.3 Hz, 8H), 0.99 (t, *J* = 7.3 Hz, 12H). ^{13}C NMR (101 MHz, $cdcl_3$) δ 176.59 (s), 156.91 (s), 148.54 (s), 147.60 (s), 128.23 (s), 126.88 (s), 125.16 (s), 121.65 (s), 119.58 (s), 113.48 (s), 111.72 (s), 50.77 (s), 29.43 (s), 20.37 (s), 14.05 (s).

Synthetic route of Compound SCU-OX-Bu: To a solution of 1-Bromo-2,6-dimethoxybenzene (369 mg, 1.7 mmol) in 5 mL of dry THF, 1.31 mL of sec-BuLi (1.3 M in hexane, 1.7 mmol) was added dropwise at -78 °C. Then the reaction mixture was stirred for 1 h at -78 °C. After that, the compound **F** (100 mg, 0.17 mmol) was added to one portion. The mixture was stirred for 12 h at room temperature. Subsequently, 2 M HCl was added and stirred for 15 min, and poured into water and extracted with DCM (20 mL) three times. The organic phase was dried with Na_2SO_4 and evaporated in vacuo. The crude product was purified by column chromatography on silica gel to give the final product as a green

solid (80 mg, yield: 66.7 %). ^{1}H NMR (400 MHz, cdcl$_3$) δ 8.08 (s, 2H), 7.94 (d, *J* = 8.9 Hz, 2H), 7.87 (d, *J* = 8.6 Hz, 4H), 7.64 (t, *J* = 8.6 Hz, 1H), 7.58 (d, *J* = 9.0 Hz, 2H), 6.85 (d, *J* = 8.4 Hz, 2H), 6.79 (d, *J* = 8.6 Hz, 4H), 3.70 (s, 6H), 3.45 – 3.35 (m, 8H), 1.68 – 1.60 (m, 8H), 1.42 (dd, *J* = 14.1, 6.9 Hz, 8H), 0.99 (t, *J* = 7.2 Hz, 12H). ^{13}C NMR (101 MHz, cdcl$_3$) δ 159.88 (s), 157.45 (s), 153.39 (s), 151.20 (s), 133.43 (s), 130.58 (s), 129.92 (s), 125.25 (s), 122.38 (d, *J* = 11.4 Hz), 112.45 (s), 111.70 (s), 110.39 (s), 104.28 (s), 55.99 (s), 50.92 (s), 29.51 (d, *J* = 17.0 Hz), 20.18 (s), 13.82 (s). HRMS (ESI) calculated $C_{49}H_{59}N_2O_3^+$ (M^+) 723.4521, found 723.4518.

Synthetic route of Compound G: The Compound C (1g, 2.6 mmol) was dissolved in 20 mL of DMF, followed by the addition of potassium carbonate (1.08 g, 7.8 mmol) and bromobutane (4.29 g, 26 mmol). The reaction mixture was stirred at 100 °C for 12 h. After cooling to room temperature, the mixture was diluted with a large amount of water and extracted with ethyl acetate. The organic phase was dried with Na_2SO_4 and evaporated in vacuo. The crude product was purified by column chromatography on silica gel to give the final product as a yellow solid (1.17 g, yield: 61.9 %). ^{1}H NMR (400 MHz, cdcl$_3$) δ 8.33 (d, *J* = 8.2 Hz, 2H), 7.65 – 7.56 (m, 8H), 6.74 (d, *J* = 8.0 Hz, 4H), 3.33 (t, *J* = 7.3 Hz, 8H), 1.63 (s, 8H), 1.35 (s, 24H), 0.93 (s, 12H). ^{13}C NMR (101 MHz, cdcl$_3$) δ 176.61 (s), 160.02 (s), 156.92 (s), 148.52 (s), 147.61 (s), 146.85 (s), 128.24 (s), 126.88 (s), 125.16 (s), 121.67 (s), 119.58 (s), 113.48 (s), 111.71 (s), 105.09 (s), 97.49 (s), 55.24 (s), 51.06 (s), 31.74 (s), 27.24 (s), 26.84 (s), 22.71 (s), 14.08 (s).

Synthetic route of Compound SCU-OX-Hex: To a solution of 1-Bromo-2,6-dimethoxybenzene (304 mg, 1.4 mmol) in 5 mL of dry THF, 1.08 mL of sec-BuLi (1.3 M in hexane, 1.4 mmol) was added dropwise at -78 °C. Then the reaction mixture was stirred for 1 h at -78 °C. After that, the compound **F** (100 mg, 0.14 mmol) was added to one portion. The mixture was stirred for 12 h at room temperature. Subsequently, 2 M HCl was added and stirred for 15 min, and poured into water and extracted with DCM (20 mL) three times. The organic phase was dried with Na_2SO_4 and evaporated in vacuo. The crude product was purified by column chromatography on silica gel to give the final product as a green solid (80.9 mg, yield: 69.1 %). ^{1}H NMR (400 MHz, cdcl$_3$) δ 8.06 (d, *J* = 1.6 Hz, 2H), 7.93 (dd, *J* = 9.0, 1.6 Hz, 2H), 7.86 (d, *J* = 9.1 Hz, 4H), 7.68 (t, *J* = 8.5 Hz, 1H), 7.61 (d, *J* = 9.0 Hz, 2H), 6.89 (d, *J* = 8.5 Hz, 2H), 6.81 (d, *J* = 9.1 Hz, 4H), 3.72 (s, 6H), 3.47 – 3.40 (m, 8H), 1.72 – 1.64 (m, 8H), 1.37 (s, 24H), 0.93 (t, *J* = 6.4 Hz, 12H). ^{13}C NMR (101 MHz, cdcl$_3$) δ 159.85 (s), 157.45 (d, *J* = 2.3 Hz), 153.37 (s), 151.18 (s), 133.44 (s), 130.58 (s), 129.93 (s), 125.26 (s), 122.38 (d, *J* = 10.7 Hz), 112.44 (s), 110.38 (s), 108.08 (s), 104.30 (s), 56.01 (s), 51.21 (s), 31.56 (s), 27.30 (s), 26.64 (s), 22.55 (s), 13.90 (s). HRMS (ESI) calculated $C_{57}H_{75}N_2O_3^+$ (M^+) 835.5773, found 835.5769.

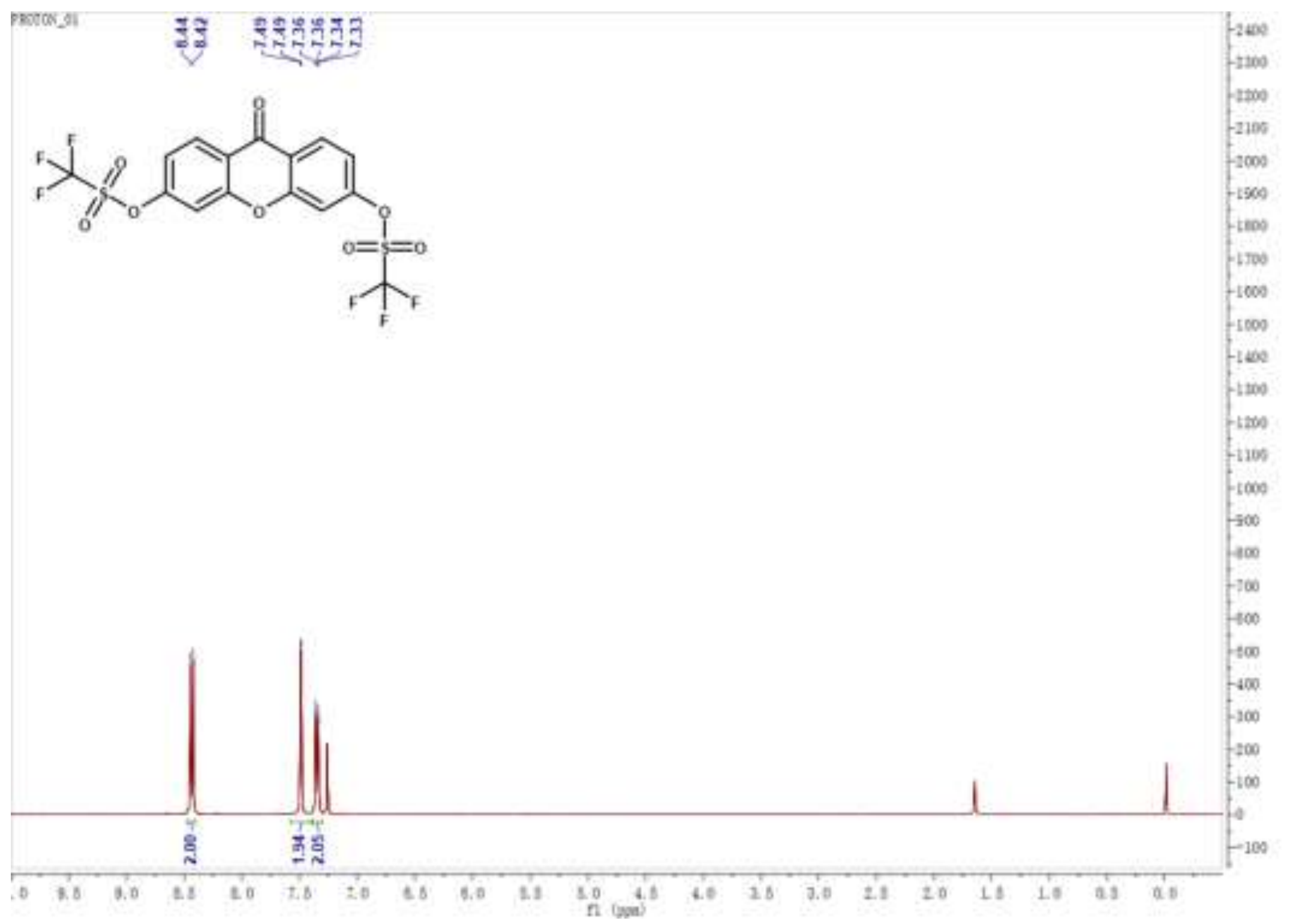

Figure S1. The ^{1}H NMR spectra of compound B in $CDCl_3$.

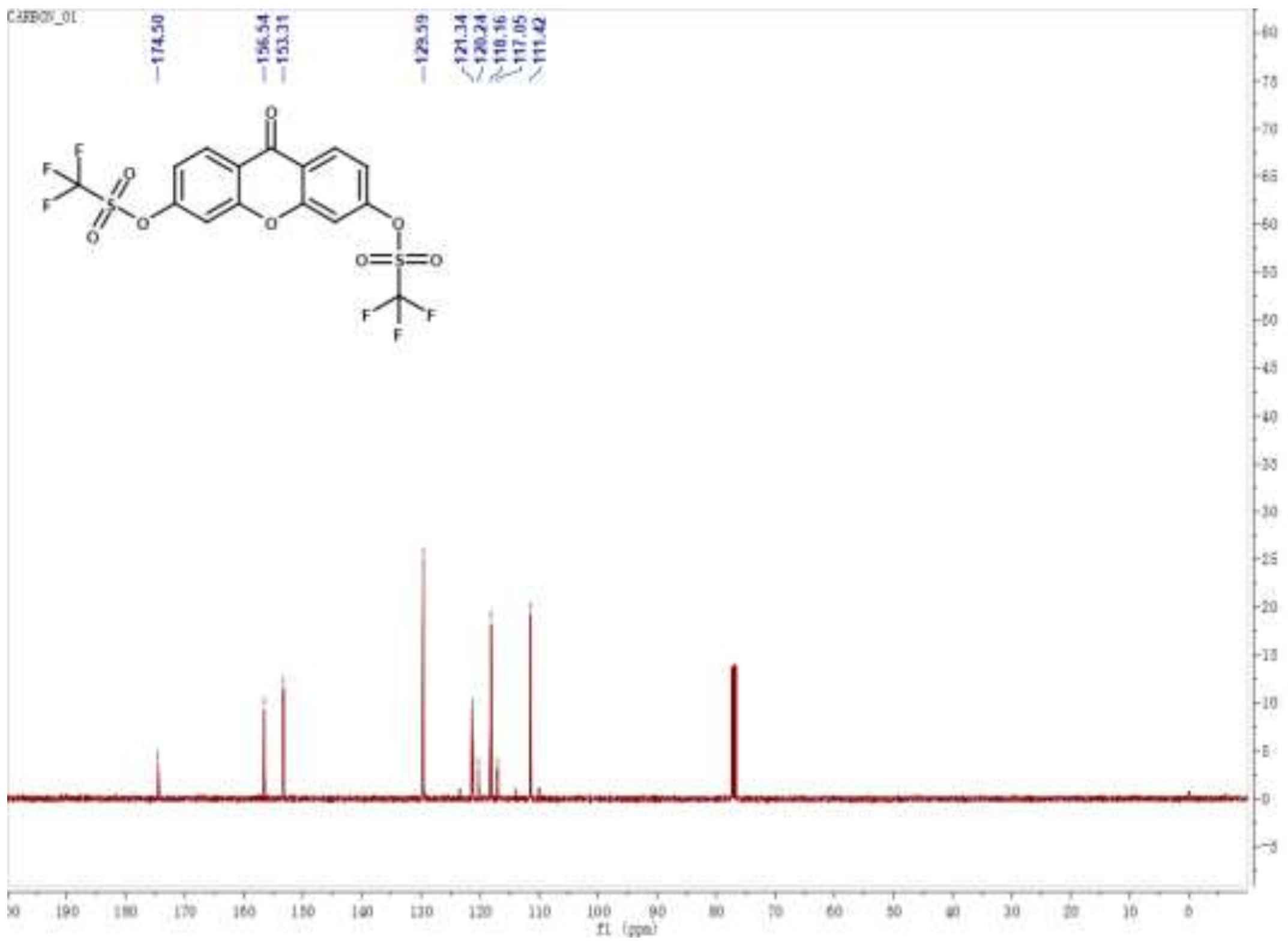

Figure S2. The ^{13}C NMR spectra of compound B in $CDCl_3$.

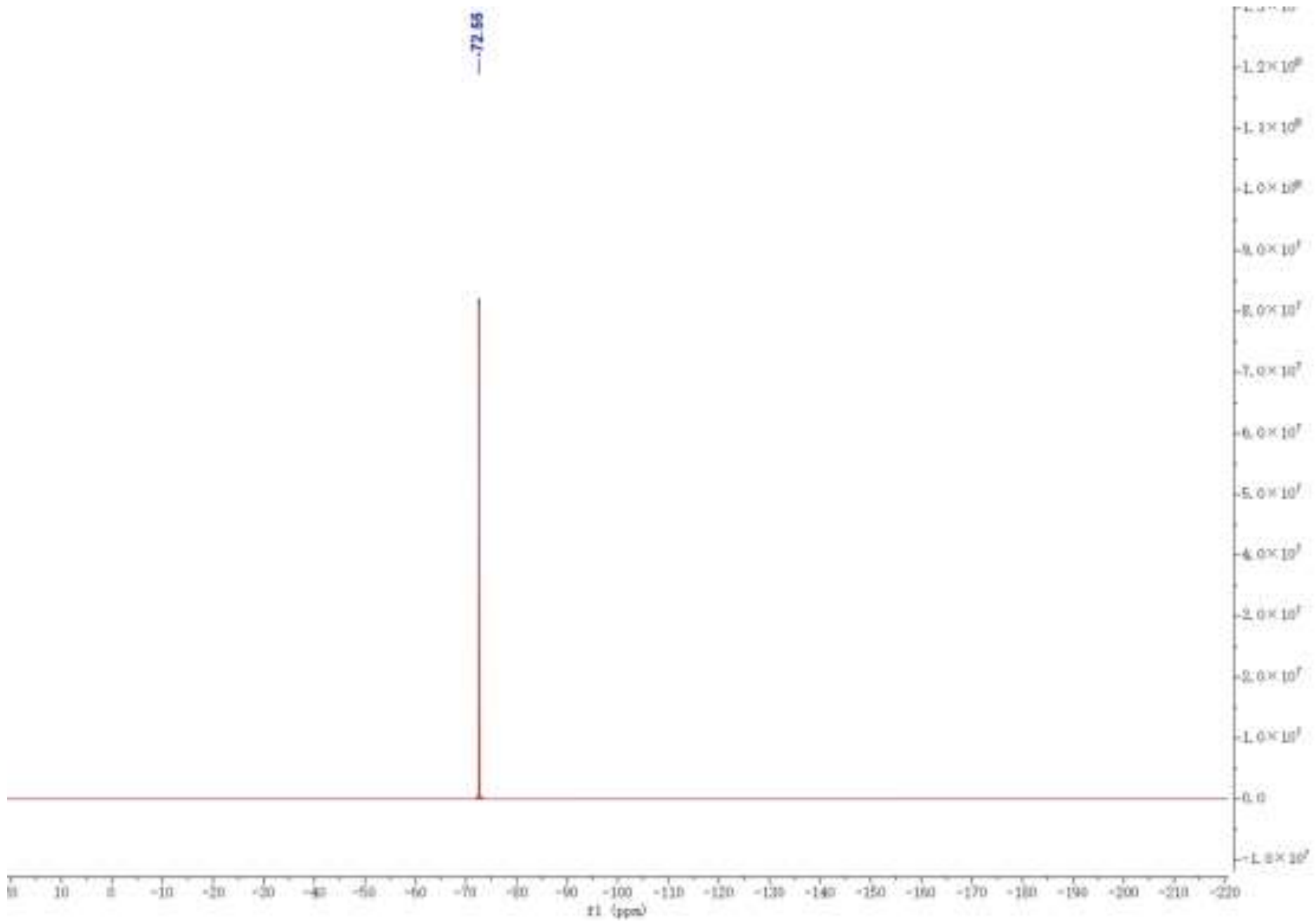

Figure S3. The ^{19}F NMR spectra of compound B in $CDCl_3$.

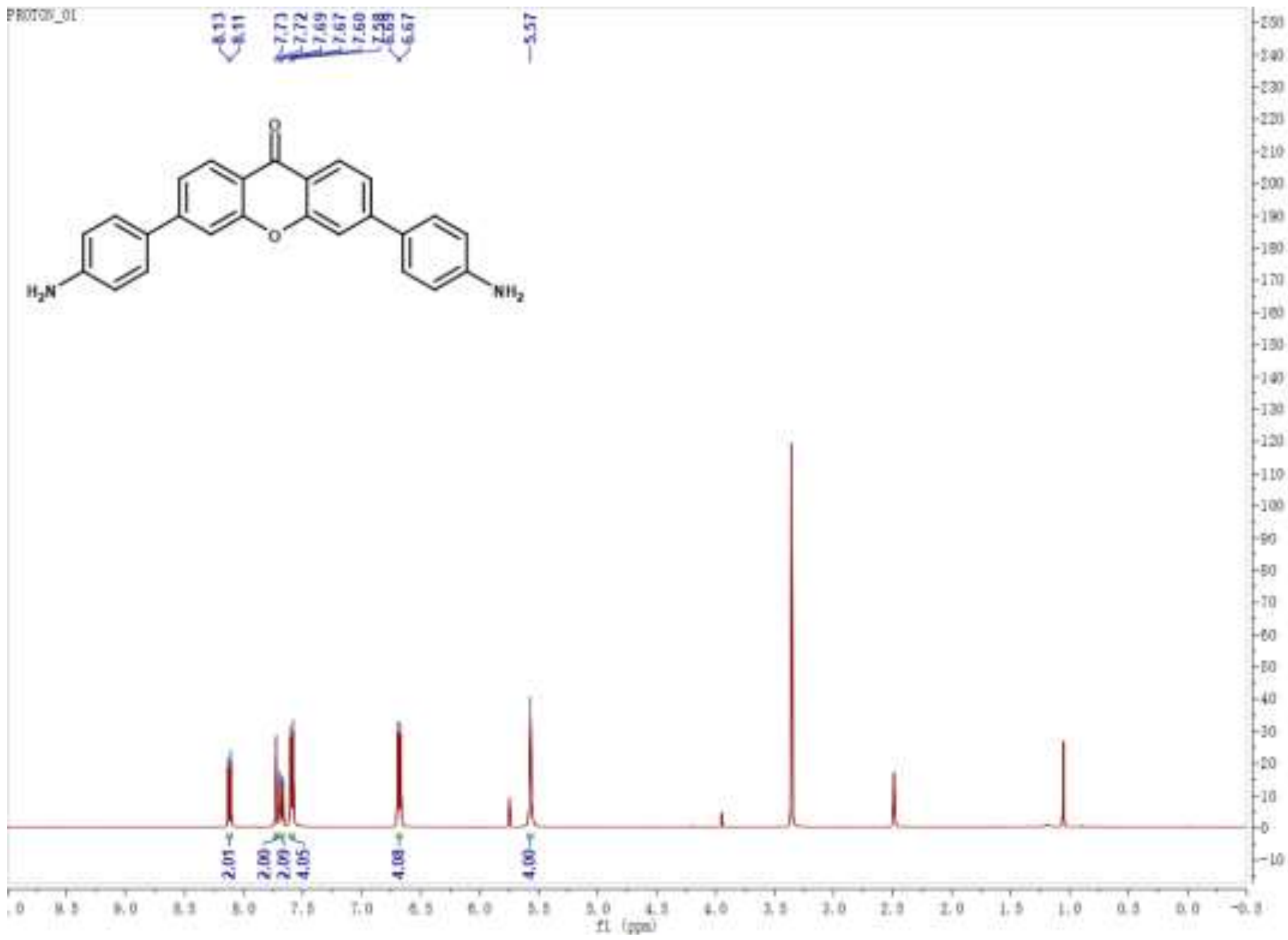

Figure S4. The ^{1}H NMR spectra of compound C in DMSO.

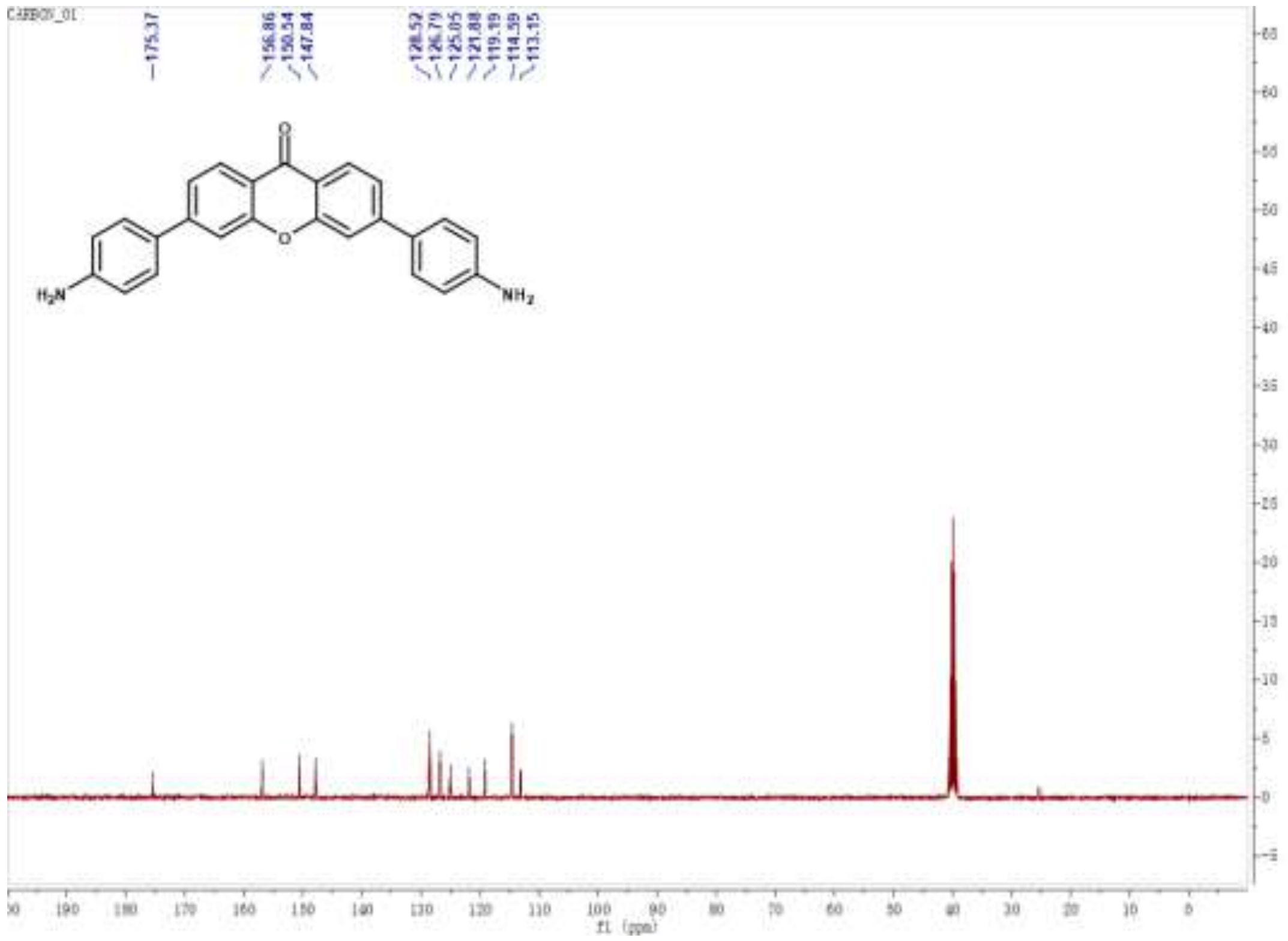

Figure S5. The ^{13}C NMR spectra of compound C in DMSO.

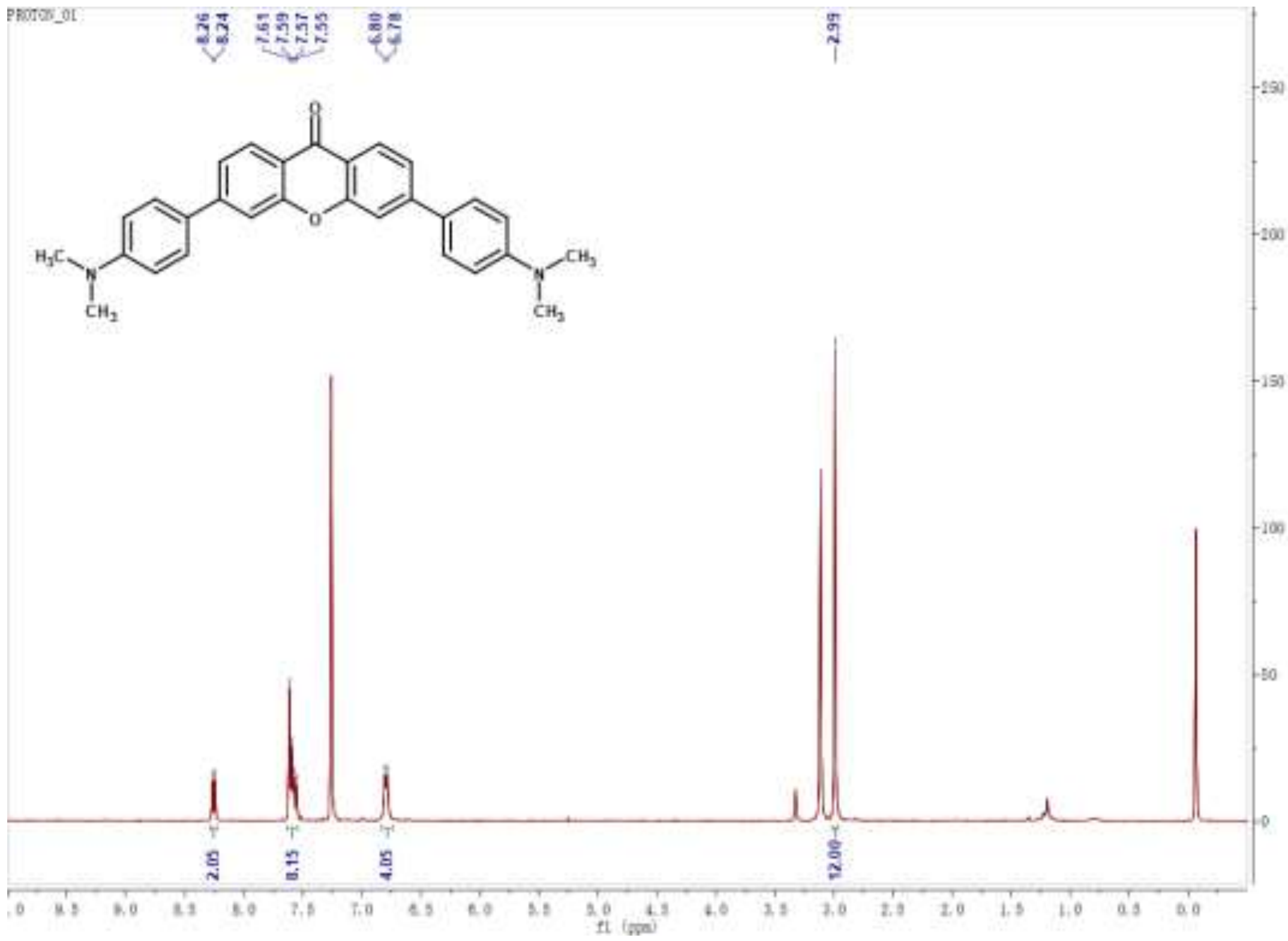

Figure S6. The ^{1}H NMR spectra of compound D in $CDCl_3$ and CD_3OD.

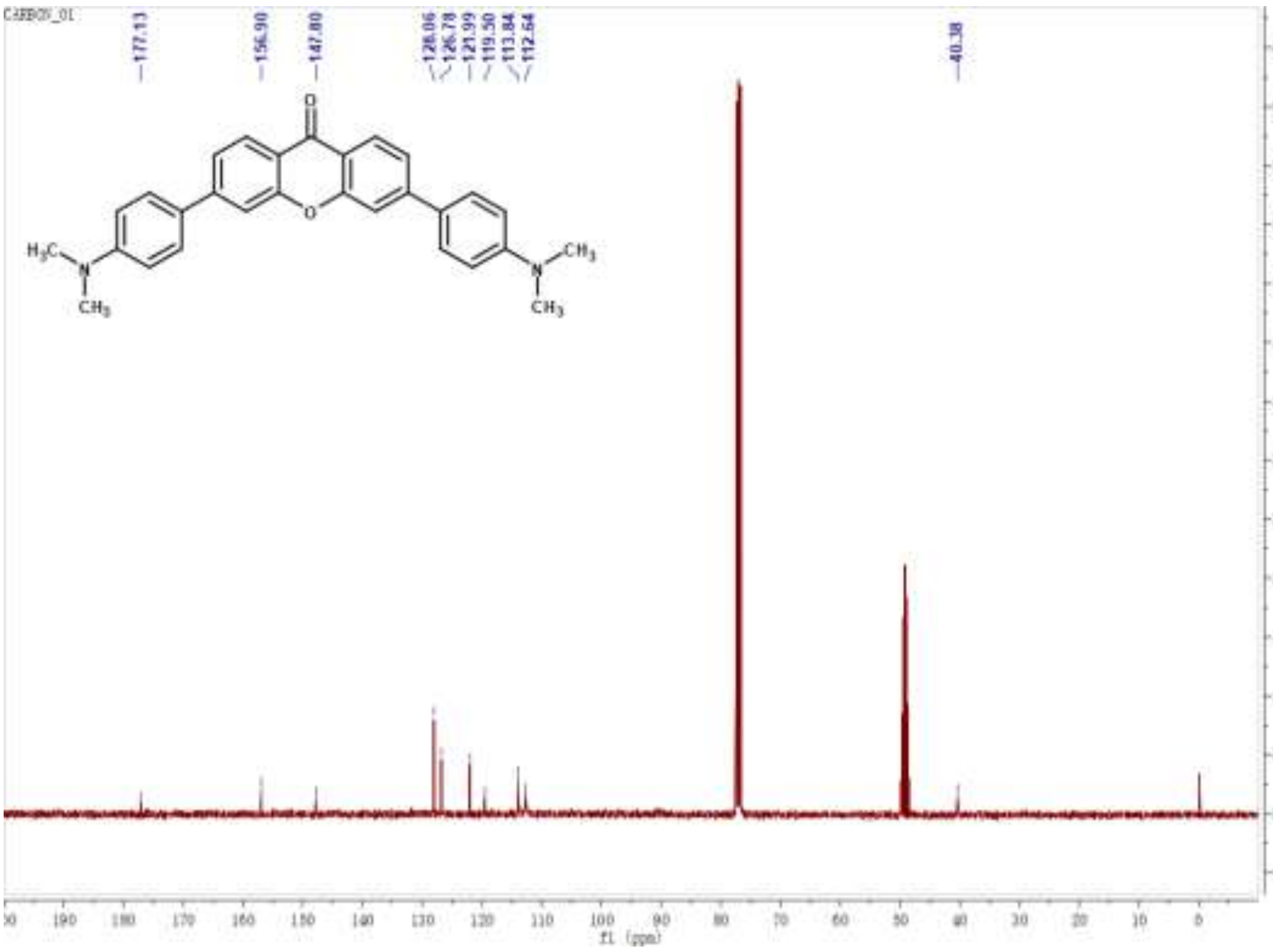

Figure S7. The ^{13}C NMR spectra of compound D in $CDCl_3$ and CD_3OD.

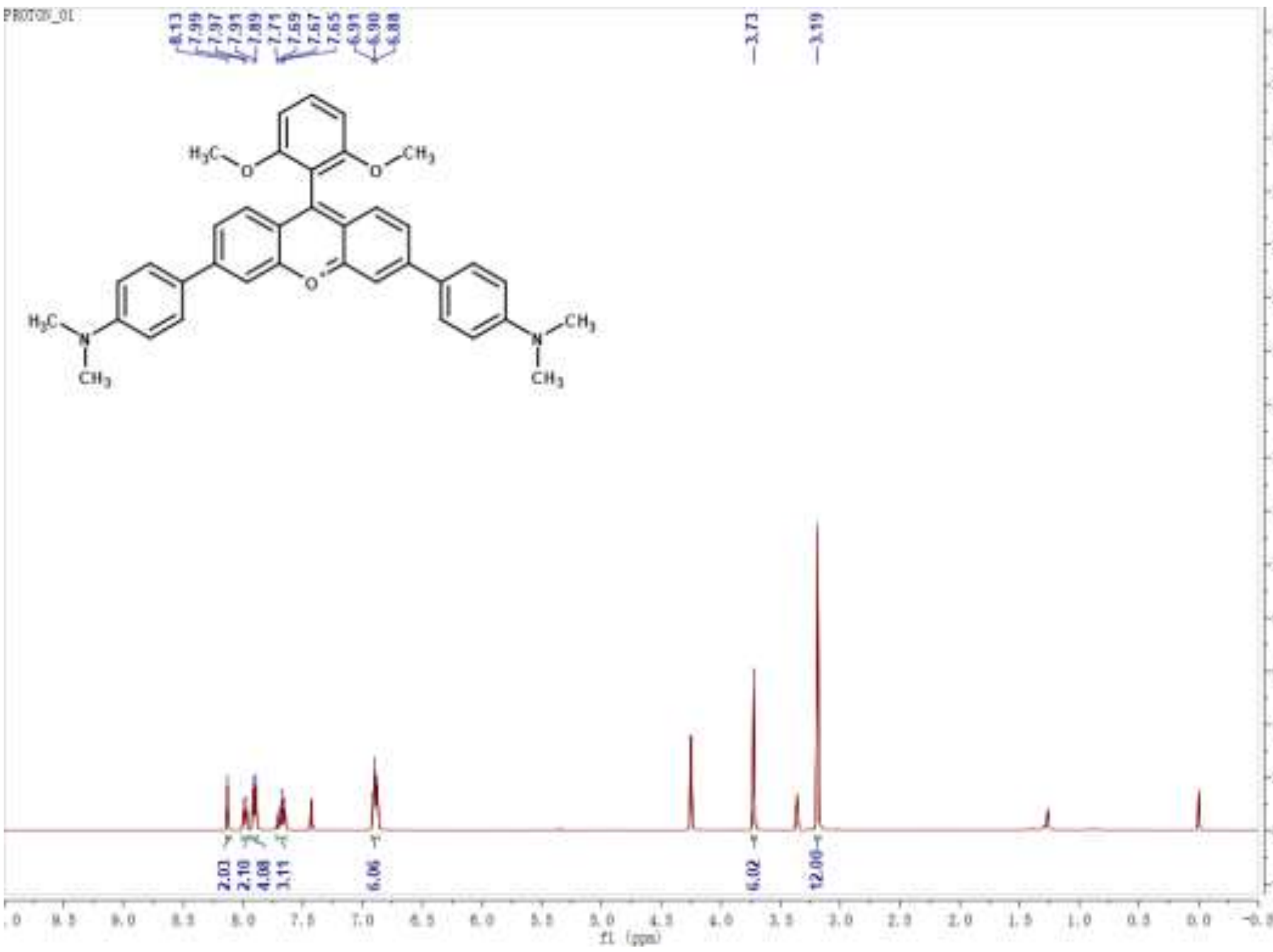

Figure S8. The ^{1}H NMR spectra of compound SCU-OX-Me in $CDCl_3$ and CD_3OD.

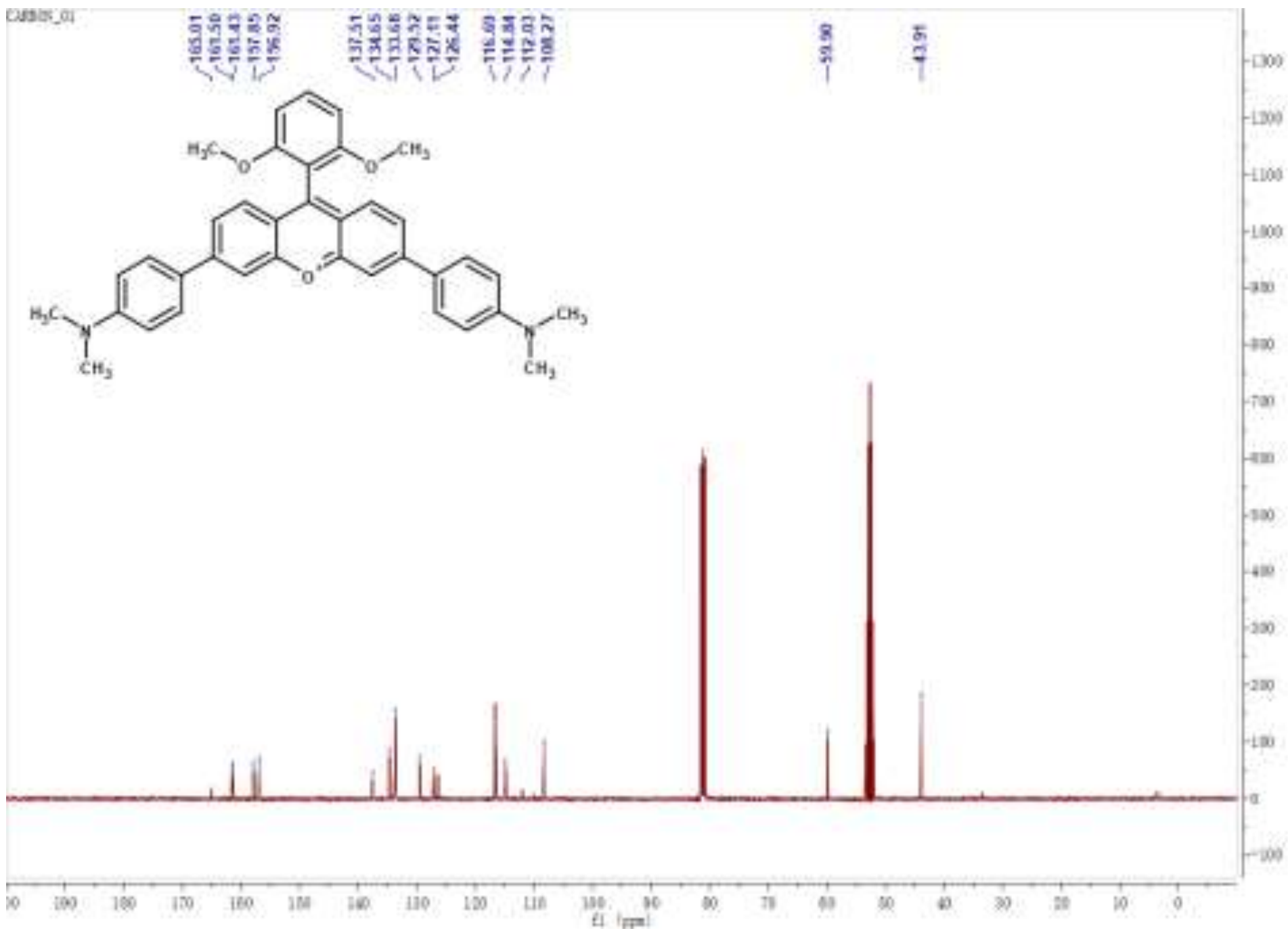

Figure S9. The ^{13}C NMR spectra of compound SCU-OX-Me in $CDCl_3$ and CD_3OD.

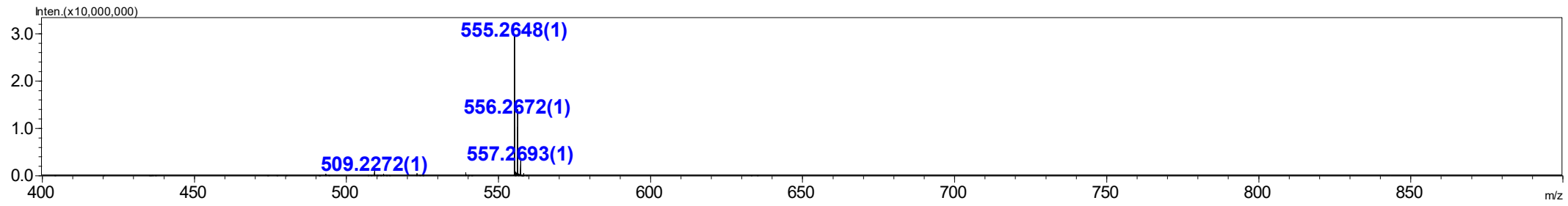

Figure S10. The HRMS spectra of compound SCU-OX-Me.

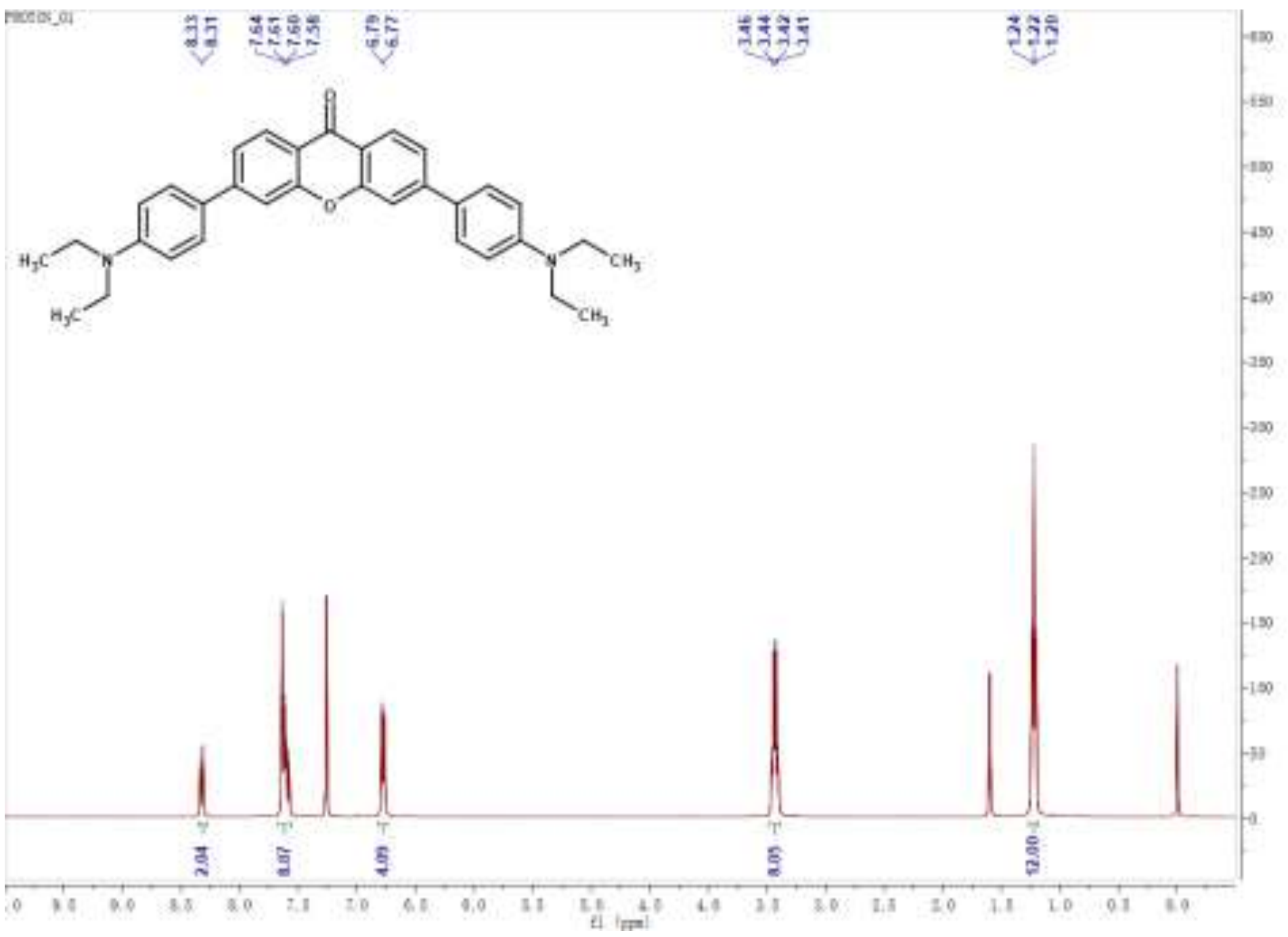

Figure S11. The ^{1}H NMR spectra of compound E in $CDCl_3$.

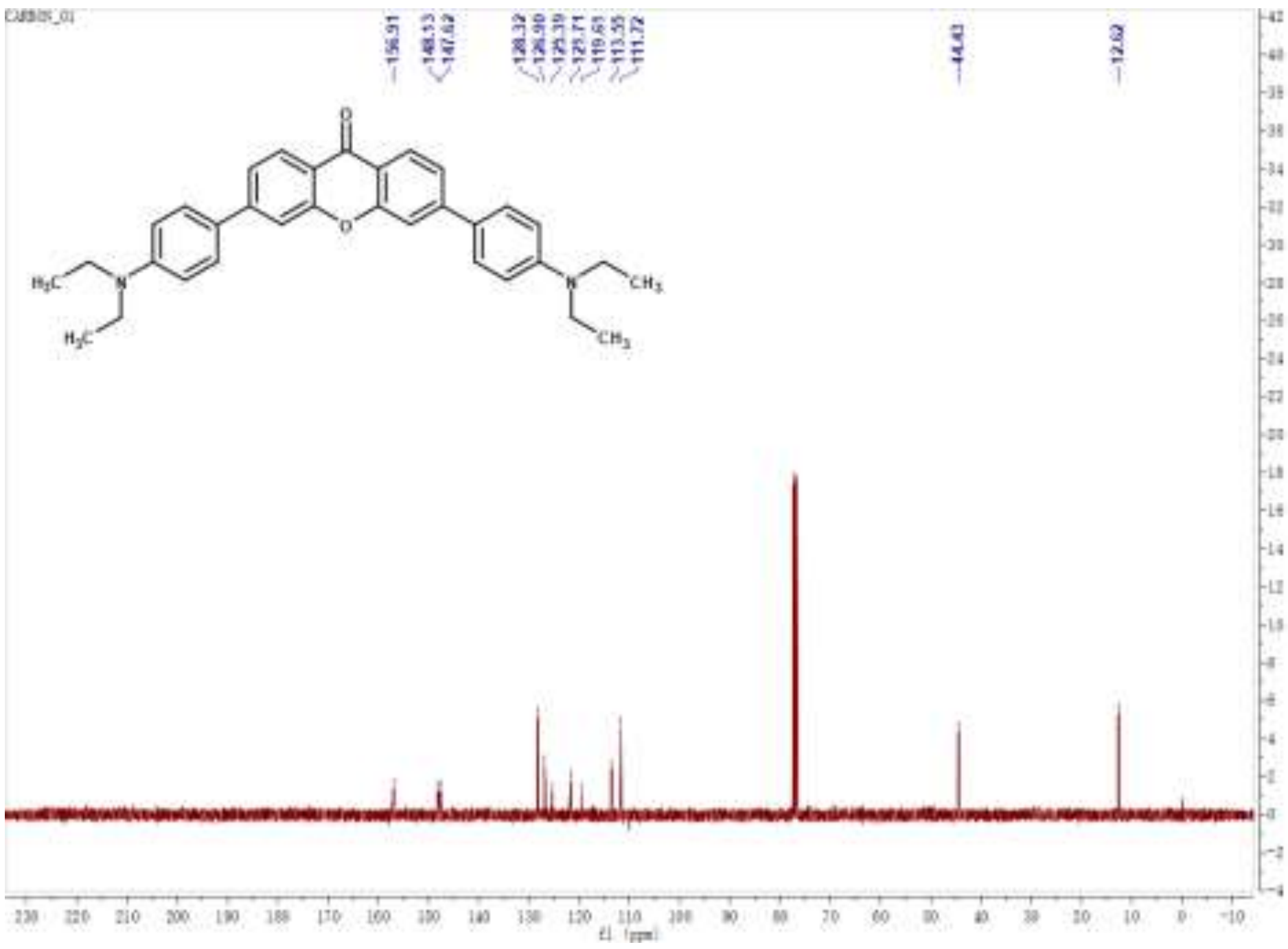

Figure S12. The ^{13}C NMR spectra of compound E in $CDCl_3$.

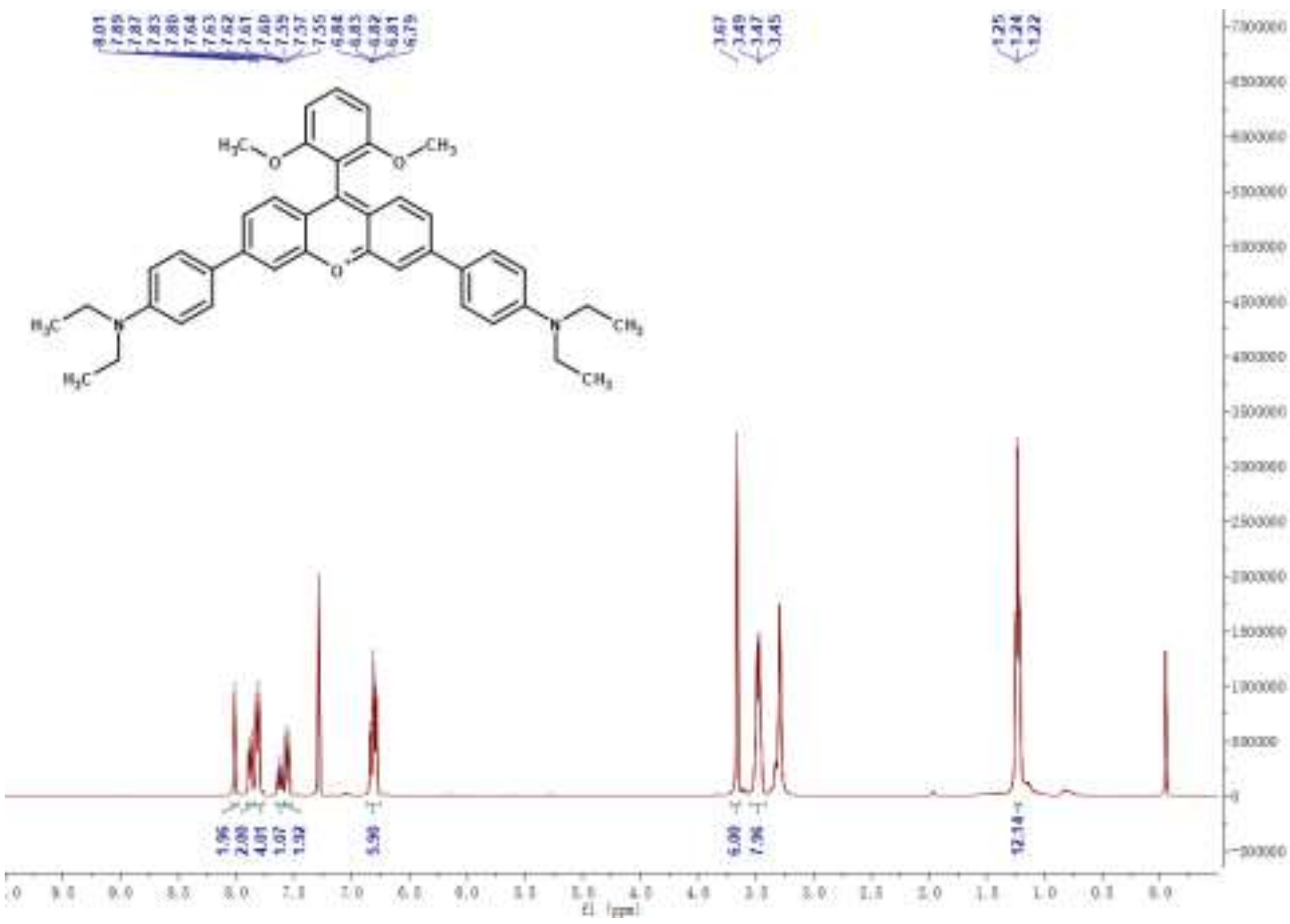

Figure S13. The ^{1}H NMR spectra of compound SCU-OX-Et in $CDCl_3$ and CD_3OD.

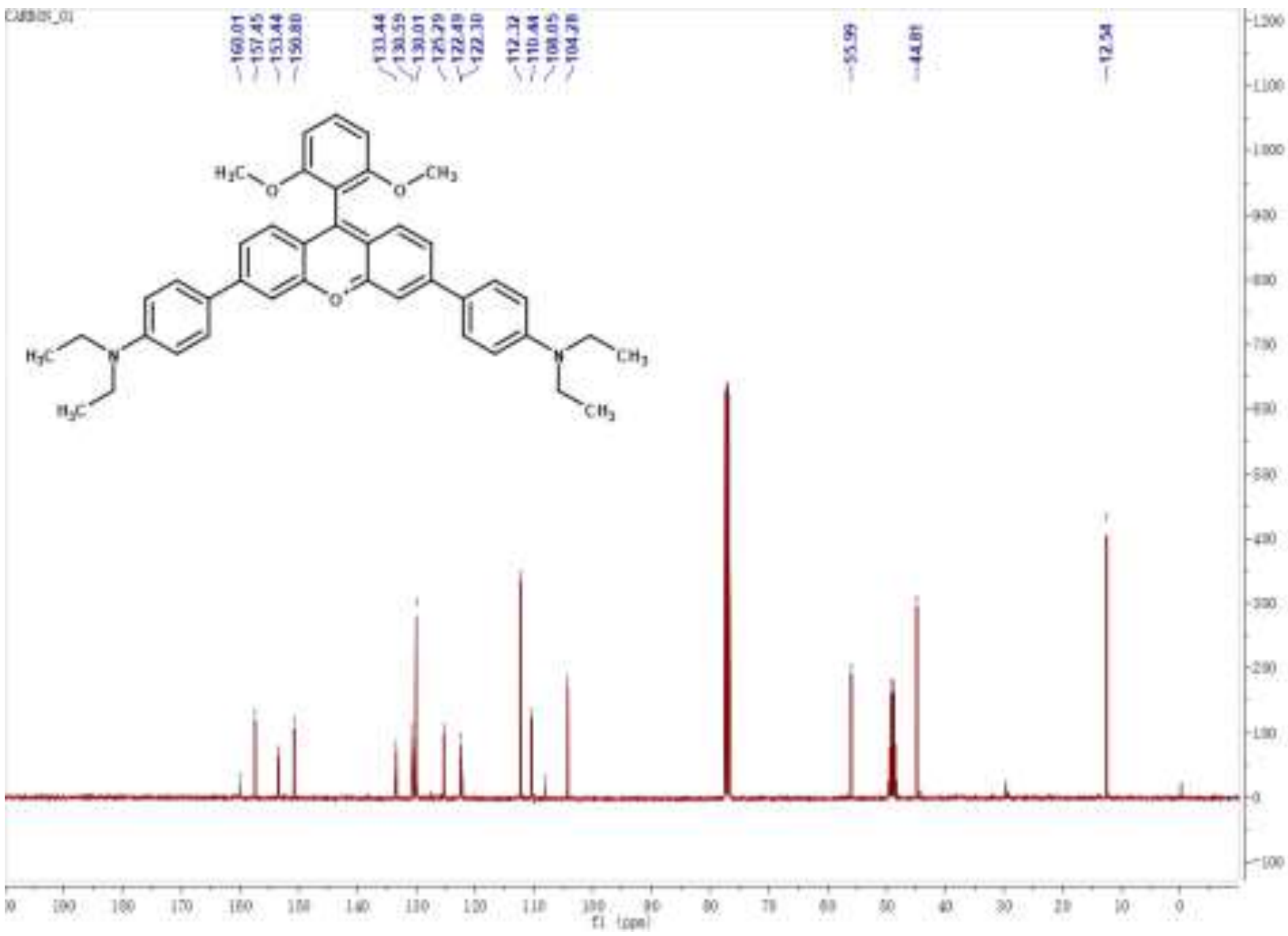

Figure S14. The ^{13}C NMR spectra of compound SCU-OX-Et in $CDCl_3$ and CD_3OD.

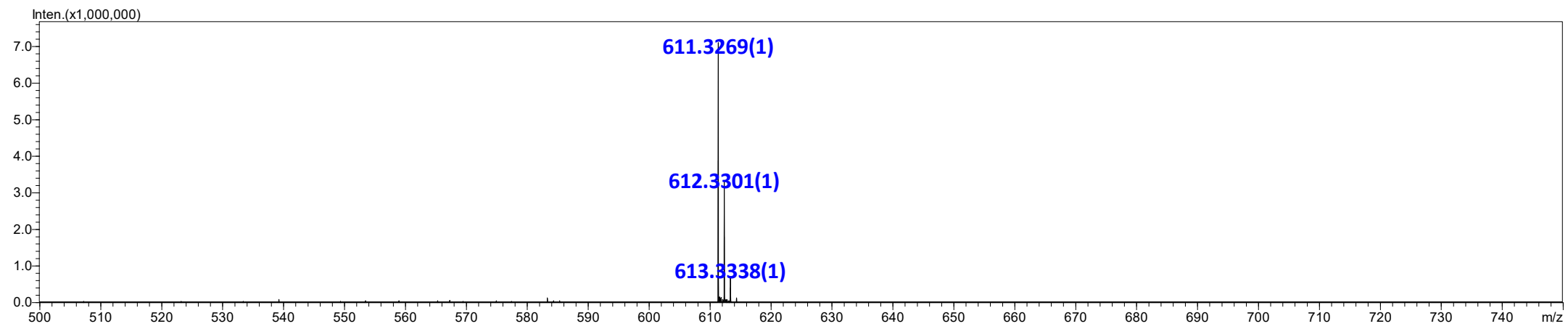

Figure S15. The HRMS spectra of compound SCU-OX-Et.

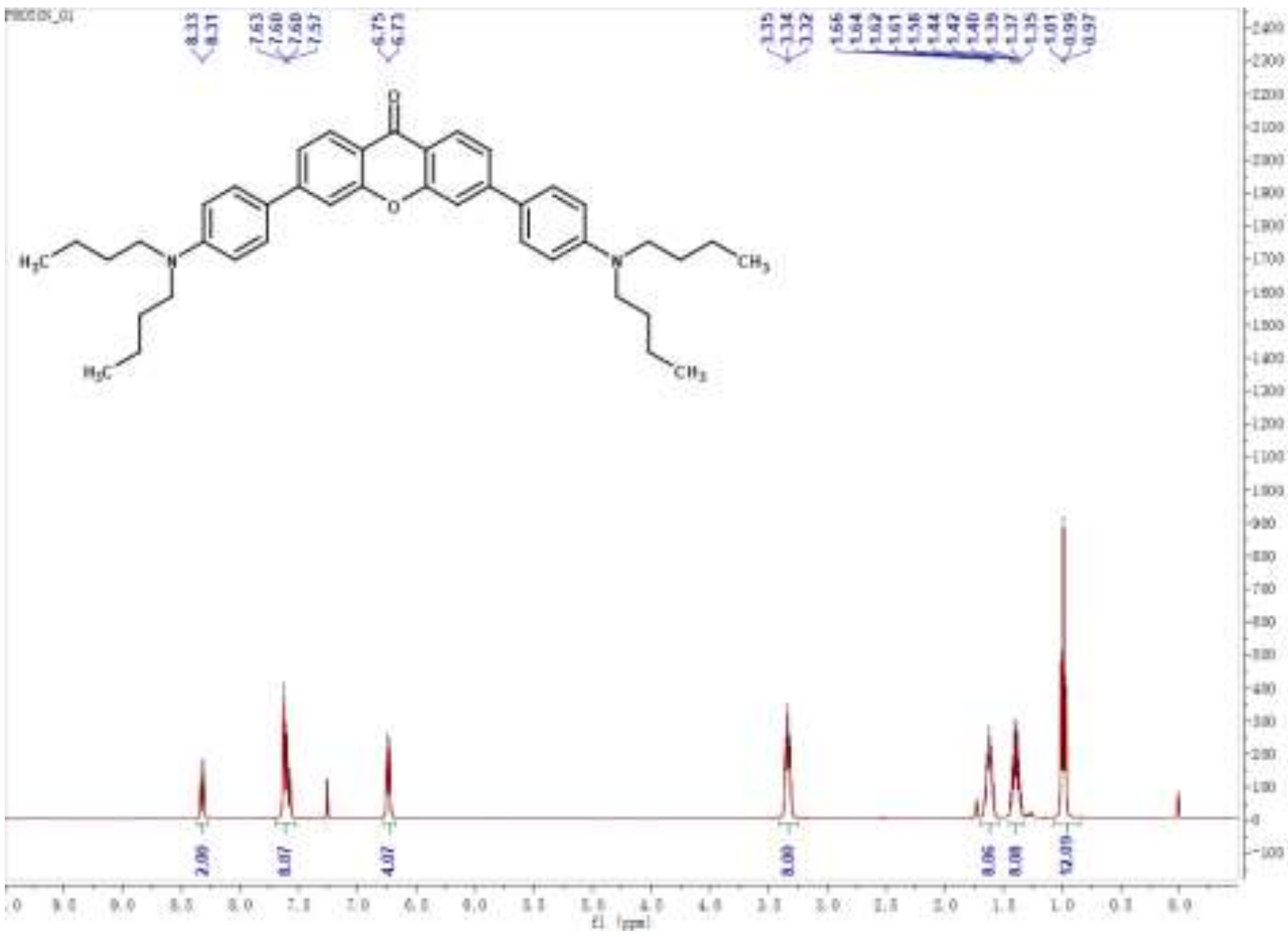

Figure S16. The ^{1}H NMR spectra of compound F in $CDCl_3$.

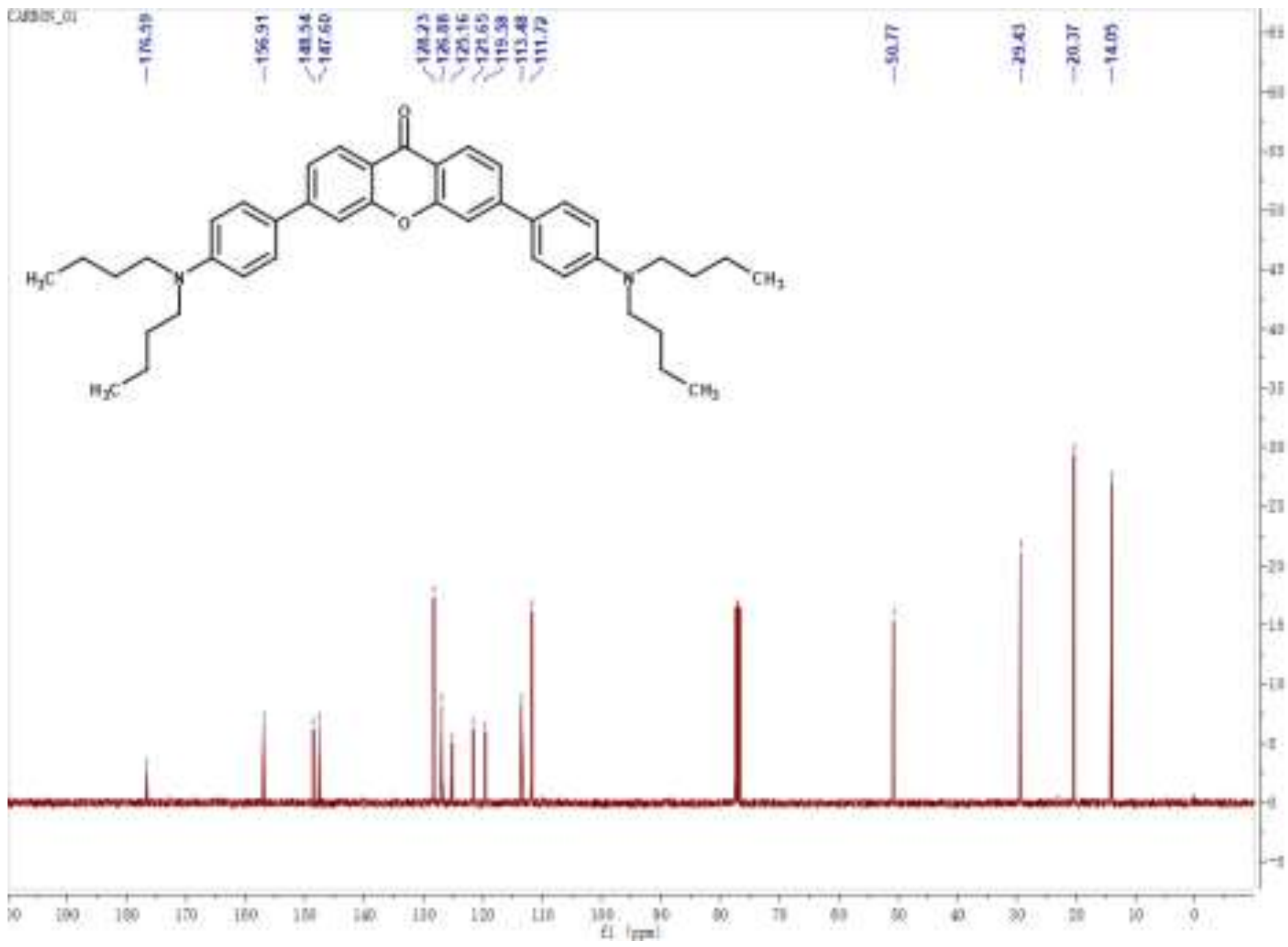

Figure S17. The ^{13}C NMR spectra of compound F in $CDCl_3$.

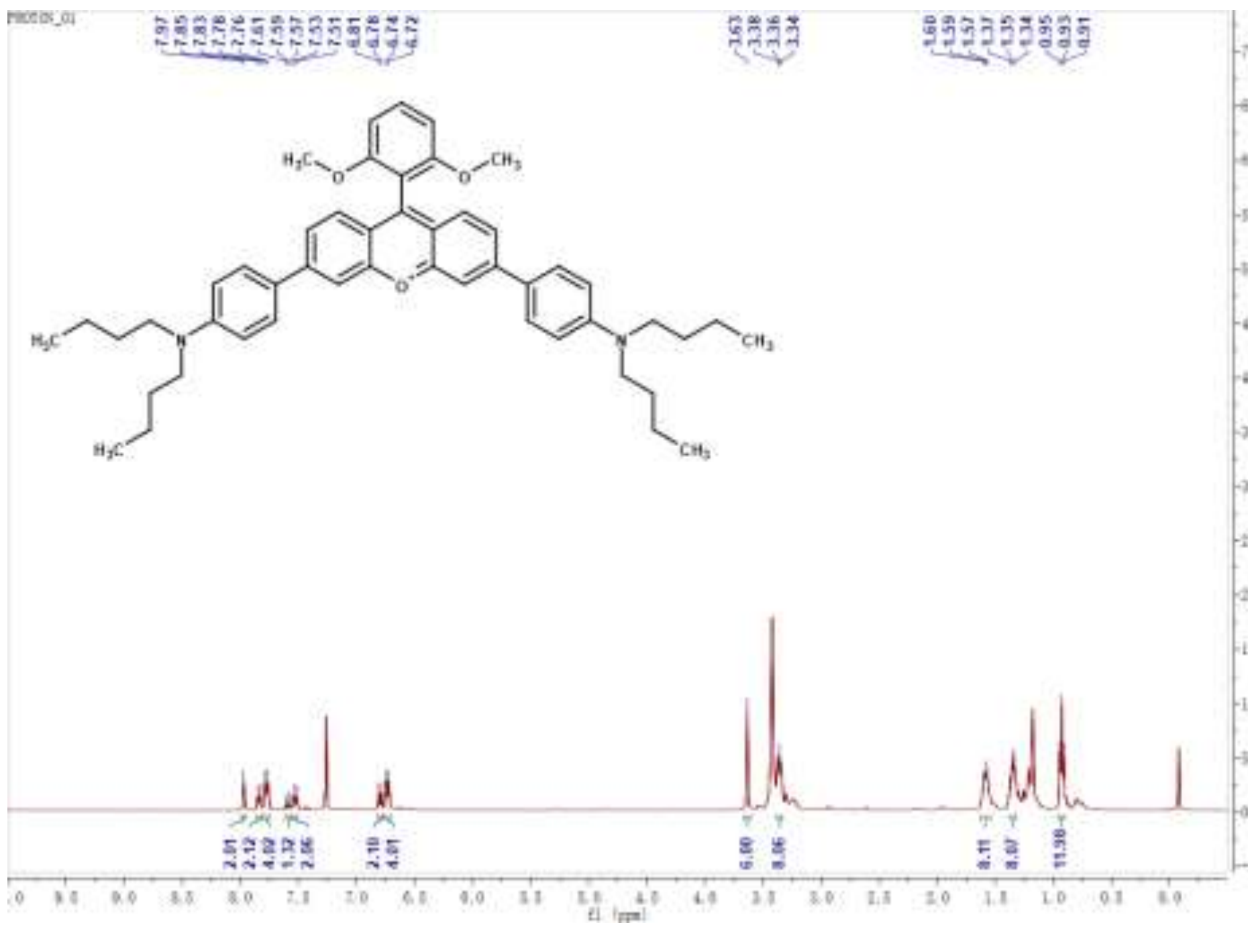

Figure S18. The ^{1}H NMR spectra of compound SCU-OX-Bu in $CDCl_3$ and CD_3OD.

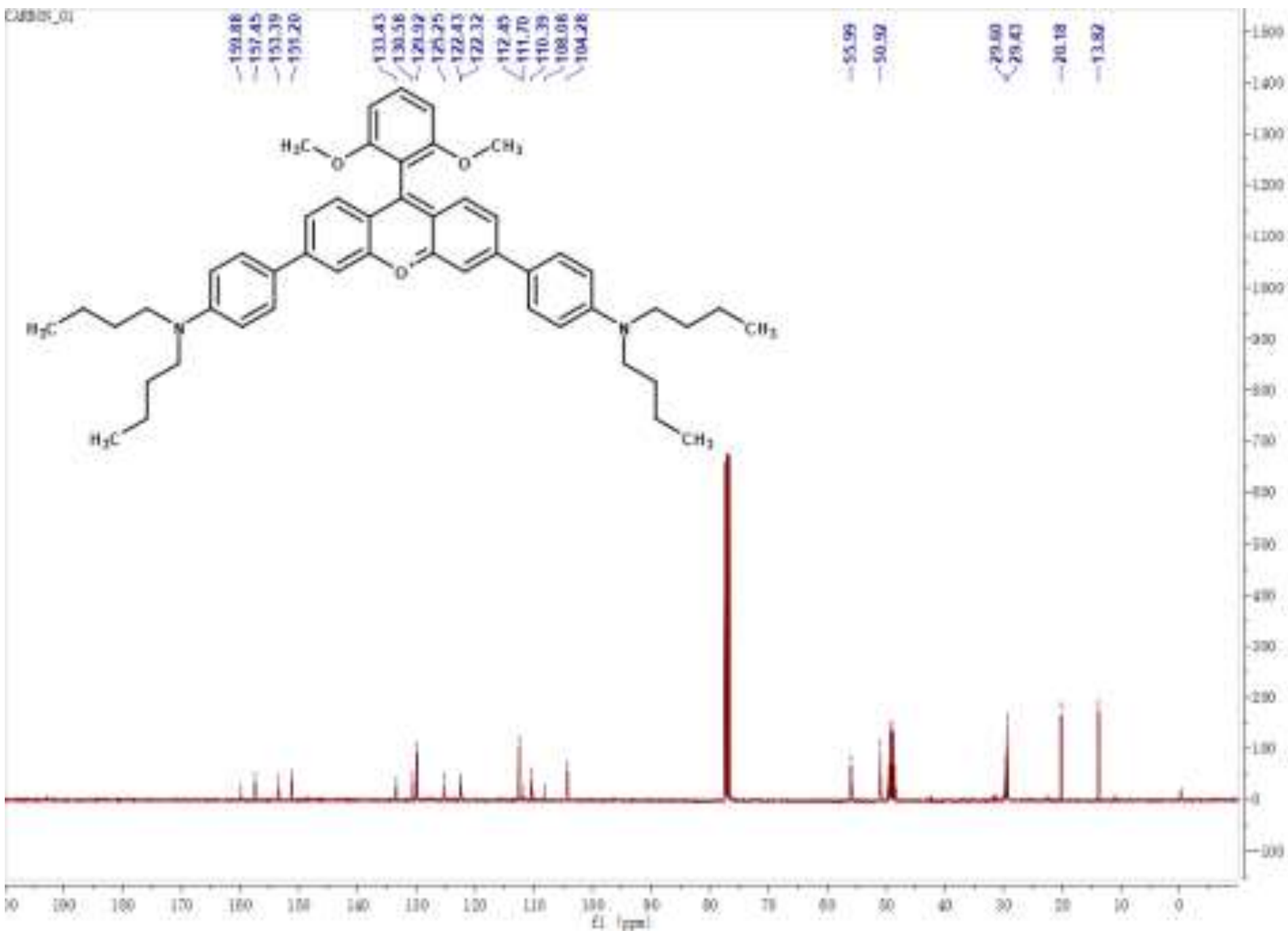

Figure S19. The ^{13}C NMR spectra of compound SCU-OX-Bu in $CDCl_3$ and CD_3OD.

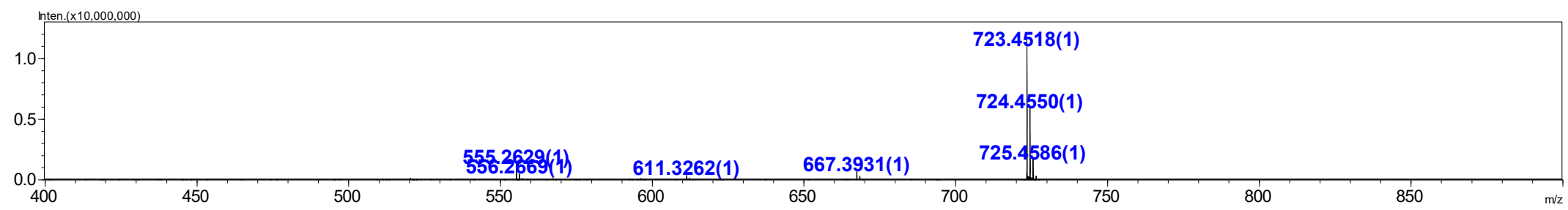

Figure S20. The HRMS spectra of compound SCU-OX-Bu.

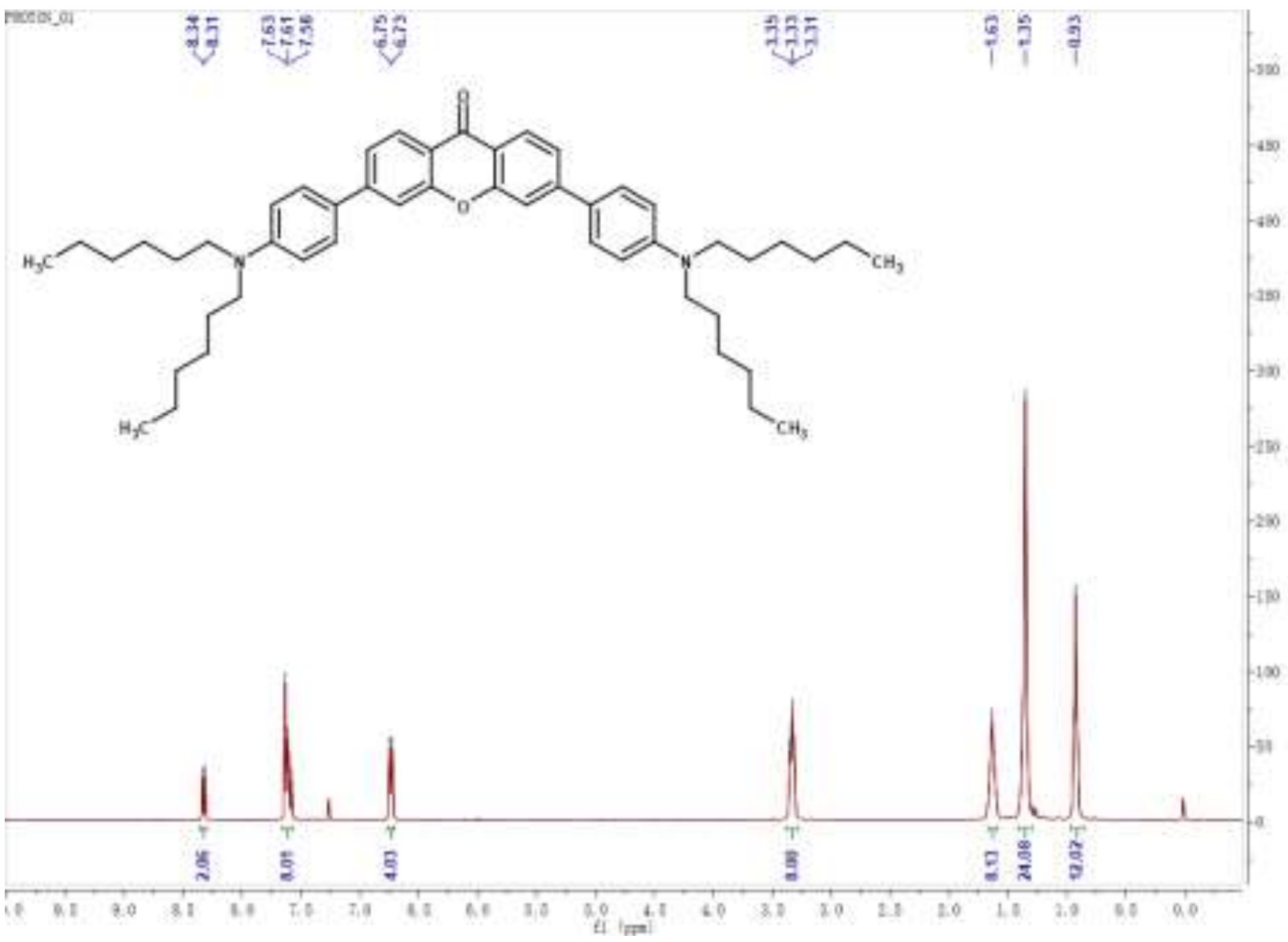

Figure S21. The ^{1}H NMR spectra of compound G in $CDCl_3$.

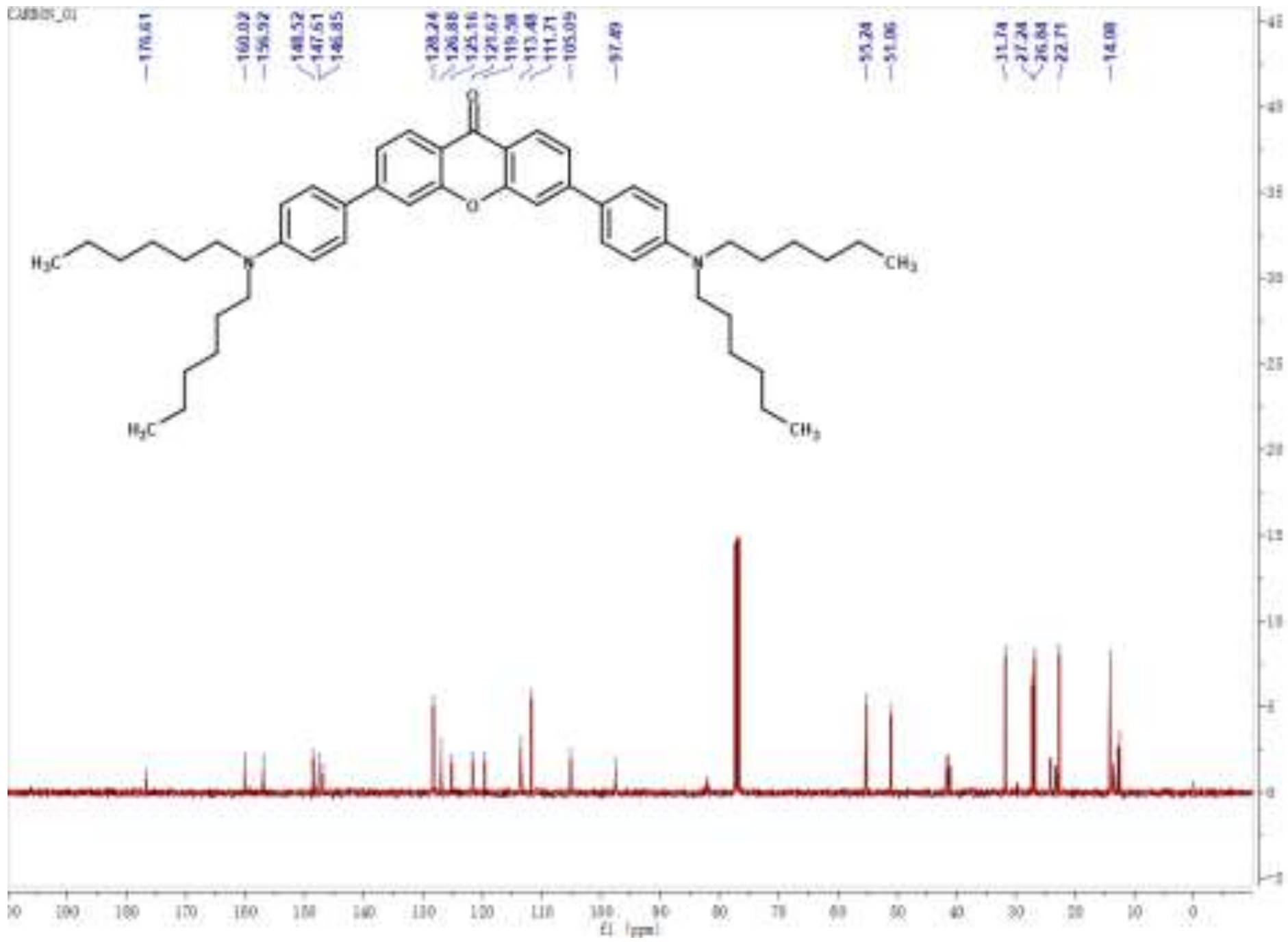

Figure S22. The ^{13}C NMR spectra of compound G in $CDCl_3$.

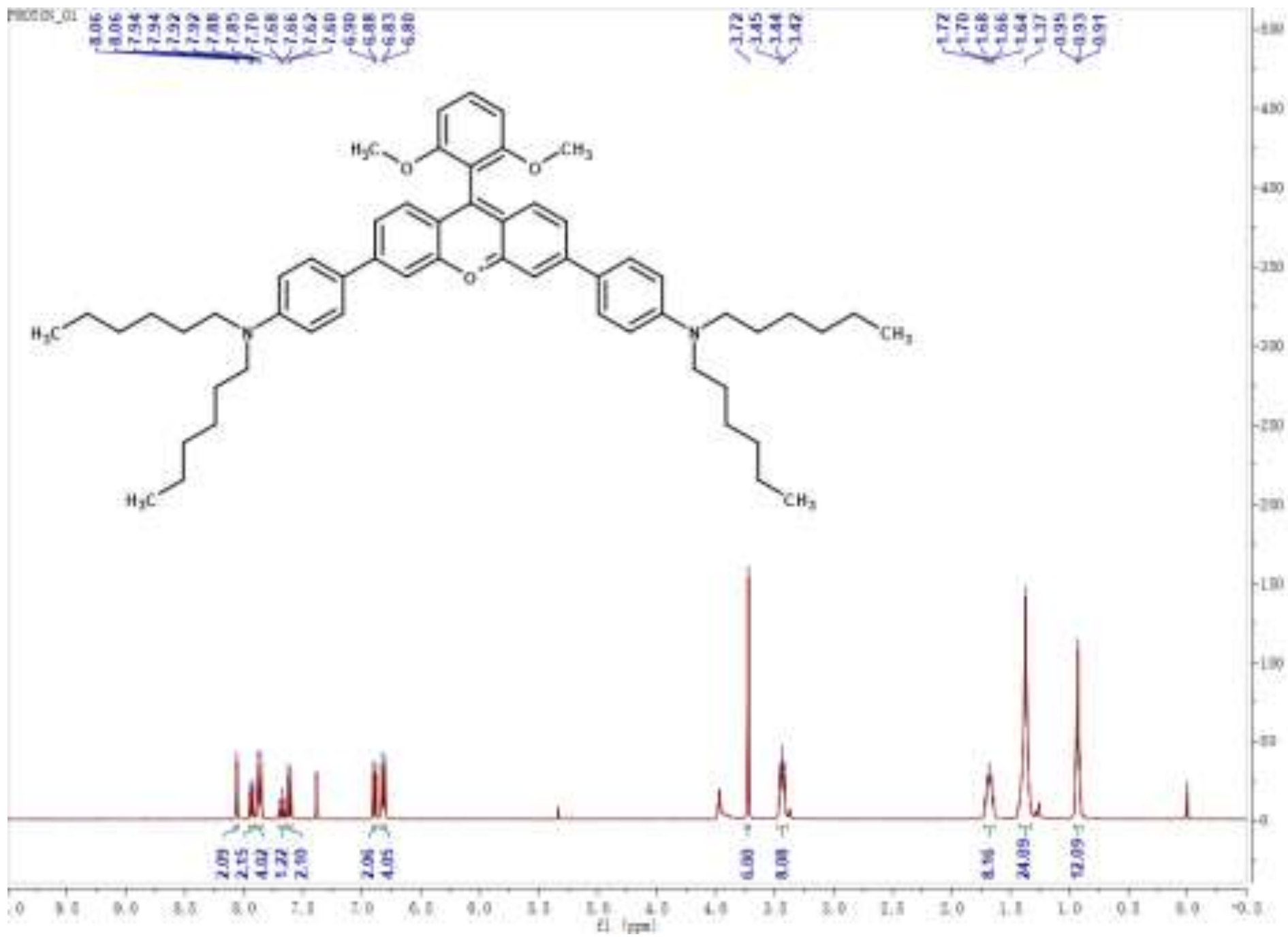

Figure S23. The ^{1}H NMR spectra of compound SCU-OX-Hex in $CDCl_3$ and CD_3OD.

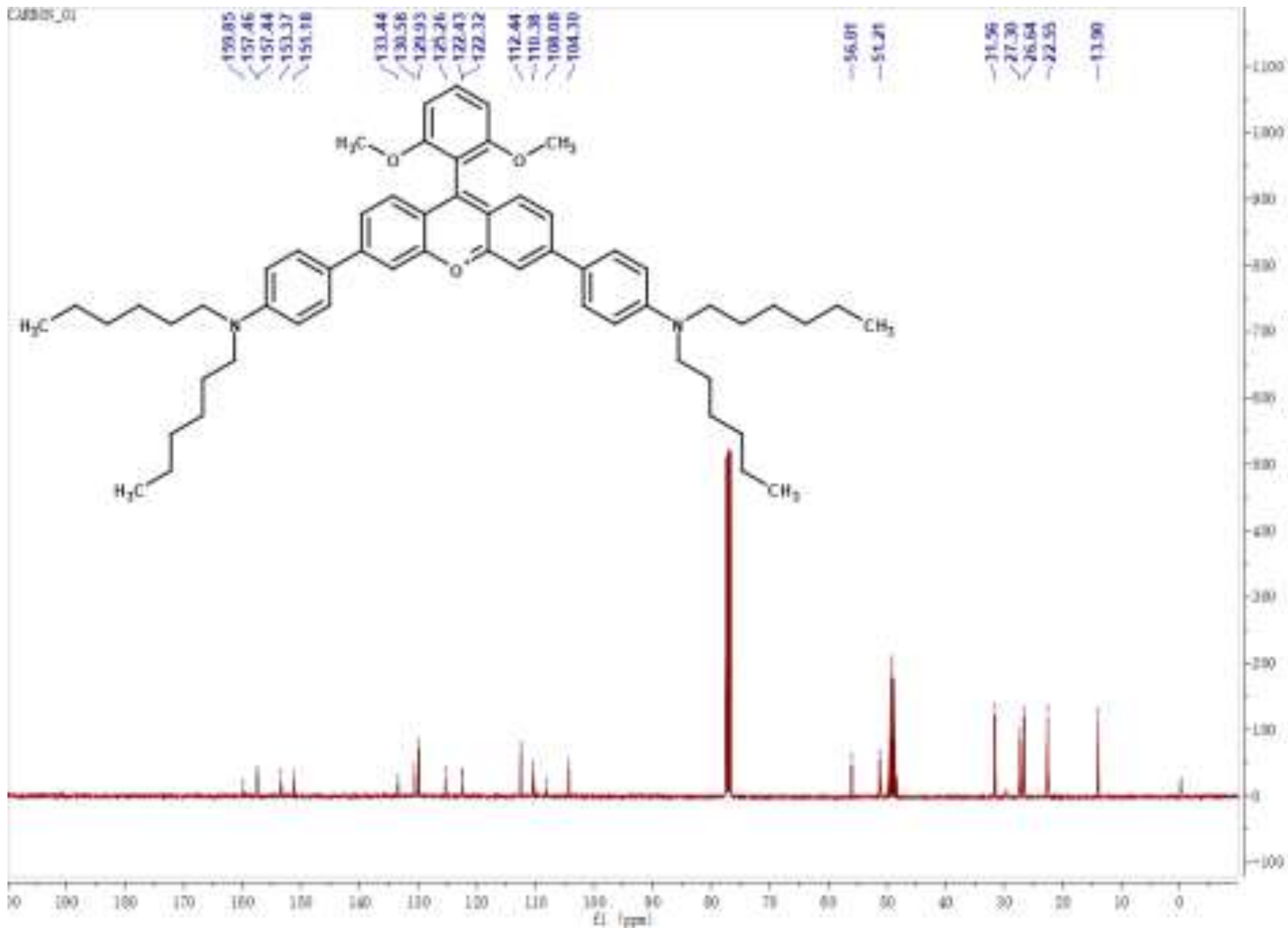

Figure S24. The ^{13}C NMR spectra of compound SCU-OX-Hex in $CDCl_3$ and CD_3OD.

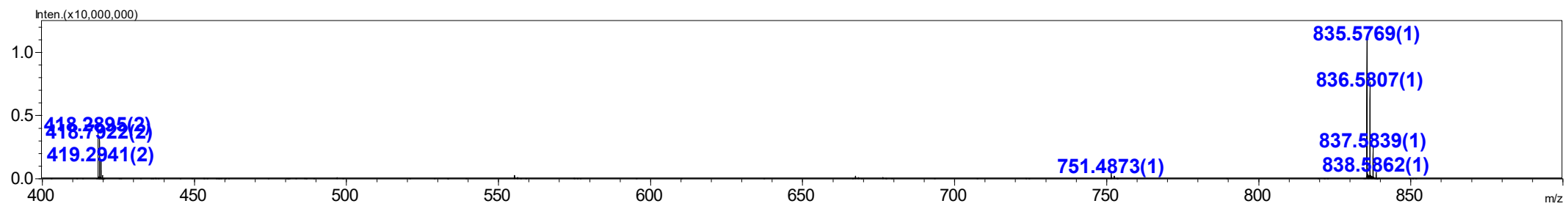

Figure S25. The HRMS spectra of compound SCU-OX-Hex.

Fluorescence QY measurement

Using IR-26 as a reference (QY = 0.05%, in dichloroethane) [1], the QYs of three molecules are determined as follows. Dilute IR-26 with 1,2-dichloroethane into a series of samples, with concentrations of 0.1, 0.2, 0.4, 0.6, and 0.8 μM at 808 nm. Then integrate the emission spectrum into the 960 to 1300 nm region. Emission spectra of different concentrations fit into a linear relationship. The QY calculation Equation (1) is as follows:

$$QY_{sample} = QY_{ref.} \times \frac{S_{sample}}{S_{ref.}} \times (\frac{n_{sample}}{n_{ref.}})^2$$

QY_{sample} is the QY of molecules, $QY_{ref.}$ is the QY of IR-26 (0.05% in dichloroethane), S_{sample} and $S_{ref.}$ are obtained by linear fitting of the integrated emission spectrum of molecules and IR-26 at a slope of 808 nm. and n_{sample} and $n_{ref.}$ are the refractive indices of dichloroethane, respectively.

Molecular Dynamics Simulations

All classical molecular dynamics (MD) simulations were performed using the GROMACS software package (version 2024.1) with an integration timestep set to 1 fs. For the treatment of force field and long-range interactions, the Particle Mesh Ewald (PME) method was adopted to calculate long-range electrostatic interactions with a Fourier grid spacing of 0.12 nm, while non-bonded interactions were truncated at a distance of 13 Å.

Temperature and pressure of the system were controlled via specific coupling schemes: temperature was maintained using velocity rescaling with a stochastic term, where the temperature coupling time constant (τT) was set to 1 ps; pressure was regulated isotropically using a Berendsen barostat, with a target pressure of 1 bar and a pressure coupling time constant (τP) of 1 ps.

The initial molecular configuration was constructed using the PACKMOL program, which randomly inserted 30 target molecules into a cubic simulation box with a side length of 8×8×8 nm. Subsequent solvation of the system was performed using GROMACS. Prior to formal production simulations, the system underwent a three-step preparation and equilibration process: (1) a heating phase, where the system was heated from 300 K to 500 K at a rate of 0.04 K/ps over a duration of 5 ns; (2) a cooling phase, where the system was cooled back to 300 K at the same rate for an additional 5 ns after reaching 500 K; (3) an equilibration phase, where the system was equilibrated at 300 K for 10 ns to ensure the density reached a steady state, thus avoiding system instability and guaranteeing the reliability of calculated system properties.

Post-simulation data analysis was conducted using the MDAnalysis tool, and molecular visualization was achieved with the VMD software.

Cytotoxicity assay

NIH-3T3 cells were seeded at a density of 1 × 10^4 cells/well in a 96-well plate and incubated for 24 h. Subsequently, the cells were treated with various concentrations of SCU-OX-Hex (2 % Tween, 20 % DMSO) in fresh culture medium. After further incubation for 12 h, the medium was removed and washed with PBS. The following steps for the CCK-8 test were carried out as same as the above-mentioned procedures. Finally, the absorbance of the products was measured at a wavelength of 450 nm by a microplate reader.

Animal and tumor model

We have revised and clarified the animal model descriptions in both the main text and the Supporting Information, as detailed below:

Both models were established using female BALB/c mice (6-7 weeks old, purchased from Beijing HFK Bioscience).

(1) Subcutaneous orthotopic tumor model (uniformly referred to as the "orthotopic/subcutaneous model" in the main text): 4T1 cells (1 × 10^6 cells, suspended in PBS) were injected into the left mammary fat pad/nipple region for dynamic monitoring of peritumoral vasculature, with a sample size of n = 2; Imaging conditions: power density of 400 mW/cm^2 , exposure time of 100 ms.

(2) Footpad tumor model (uniformly referred to as the "footpad/sole tumor model" in the main text): 4T1 cells (1 × 10^6 cells, suspended in PBS) were injected into the plantar surface of the foot for identification and resection-guided imaging of metastatic lymph nodes, with a sample size of n = 2. Imaging conditions: power density of 400 mW/cm^2 , exposure time of 100 ms.

In vivo NIR-II fluorescence imaging

For NIR-II fluorescent imaging, the 4T1 breast tumor-bearing mice were administered with 200 μL of SCU-OX-Hex (1mM) through the tail vein. Then, the in vivo NIR-II fluorescent images at predetermined time intervals post-injection were subsequently captured on the NIR-II in vivo imaging system with the excitation of 808 nm. All animal studies were approved by the Sichuan University Animal Charity Protection and Treatment Committee and performed in accordance with humane care and use of research animals, approval No. 20240226173.

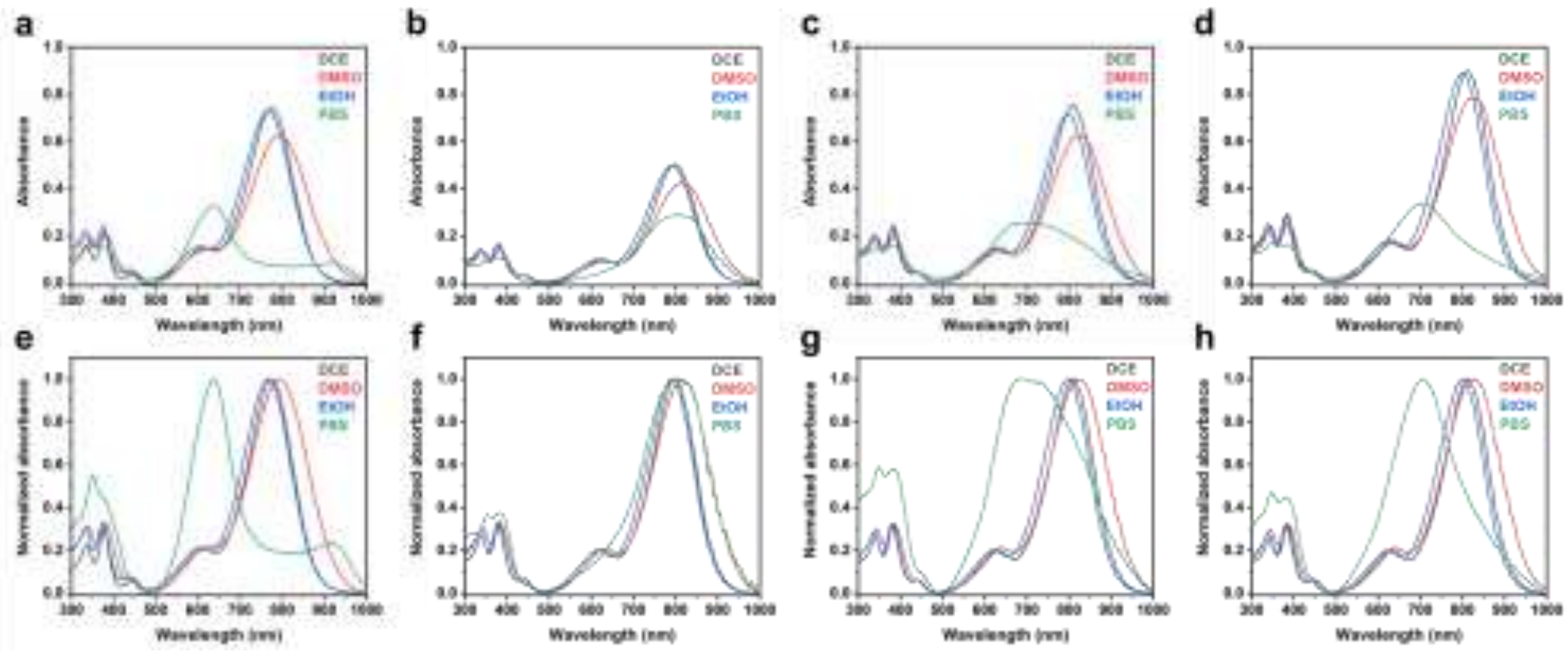

Figure S26. (a-d) Absorption spectrum of SCU-OX-Me, SCU-OX-Et, SCU-OX-Bu, SCU-OX-Hex in different solvents. (e-h) The normalized absorption spectrum of SCU-OX-Me, SCU-OX-Et, SCU-OX-Bu, SCU-OX-Hex in various solvents.

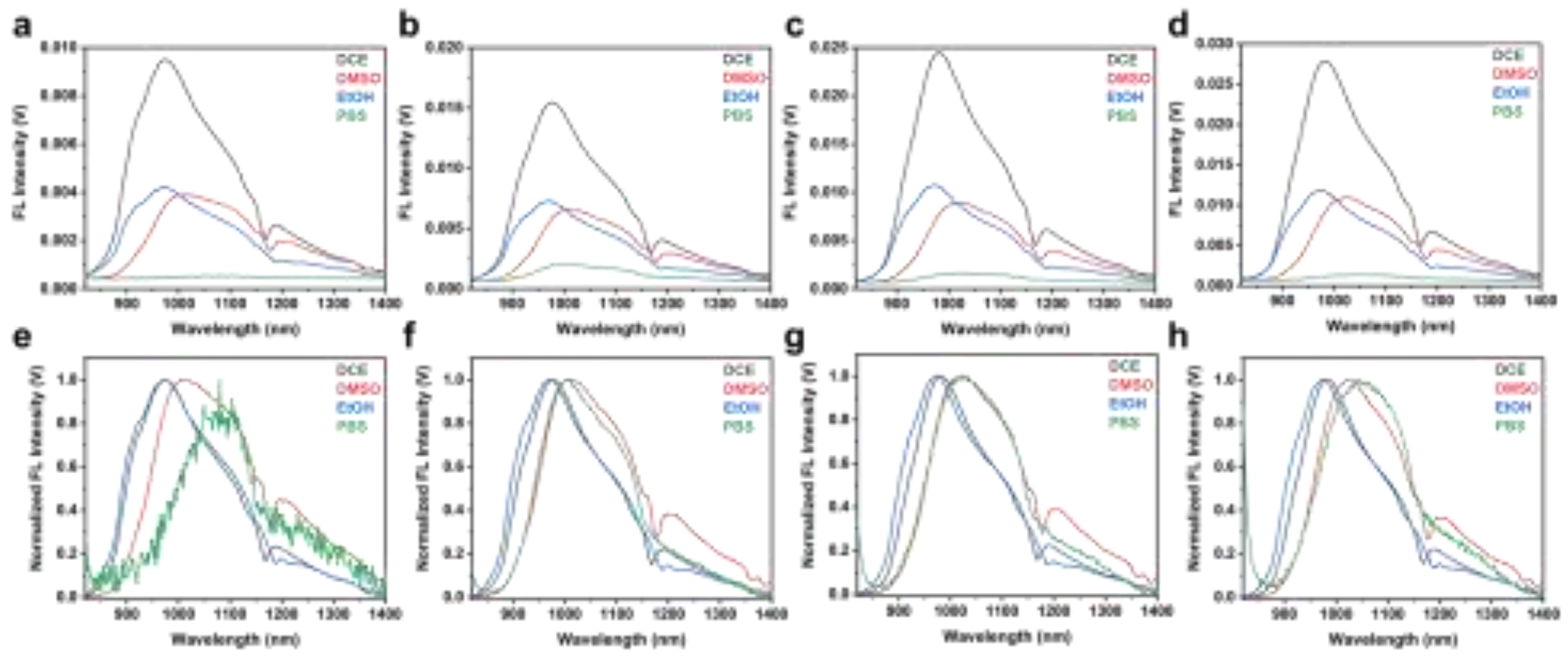

Figure S27. (a-b) FL spectrum of SCU-OX-Me, SCU-OX-Et, SCU-OX-Bu, SCU-OX-Hex in different solvents. (e-f) Normalized FL spectrum of SCU-OX-Me, SCU-OX-Et, SCU-OX-Bu, SCU-OX-Hex in different solvents with 10 μM.

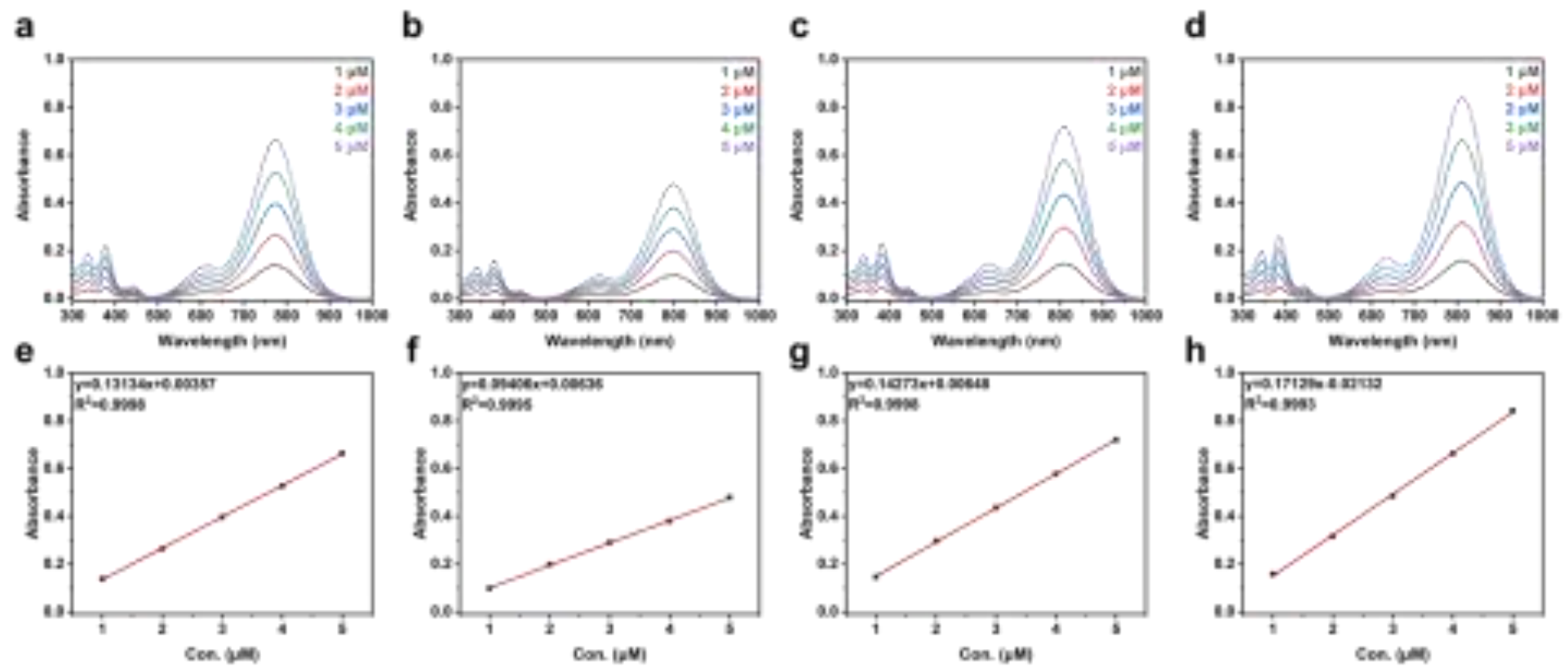

Figure S28. (a-d) The absorption spectrum of SCU-OX-Me, SCU-OX-Et, SCU-OX-Bu, SCU-OX-Hex in DCE solvent with various concentrations (1-5 μM). (E-H) the maximum absorption value of SCU-OX-Me, SCU-OX-Et, SCU-OX-Bu, SCU-OX-Hex in DCE increases linearly with concentration from 1 to 5 μM.

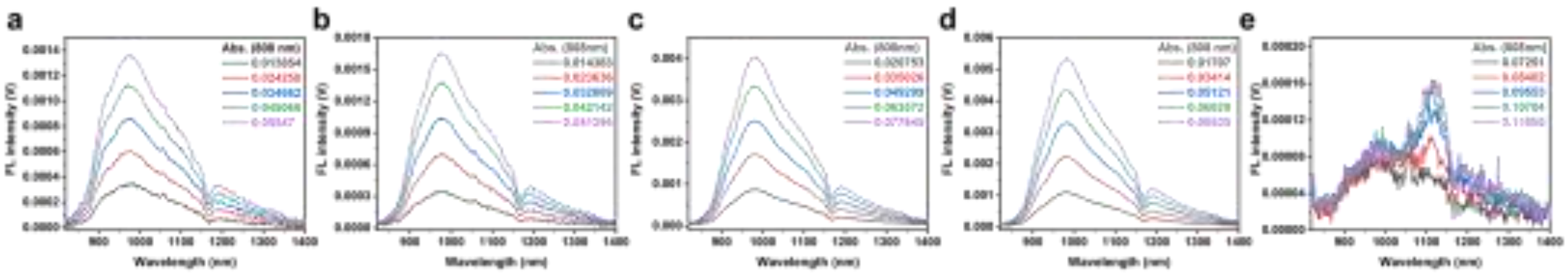

Figure S29. The FL spectrum of SCU-OX-Me, SCU-OX-Et, SCU-OX-Bu, SCU-OX-Hex, IR-26 in DCE with five various optical density values at 808 nm.

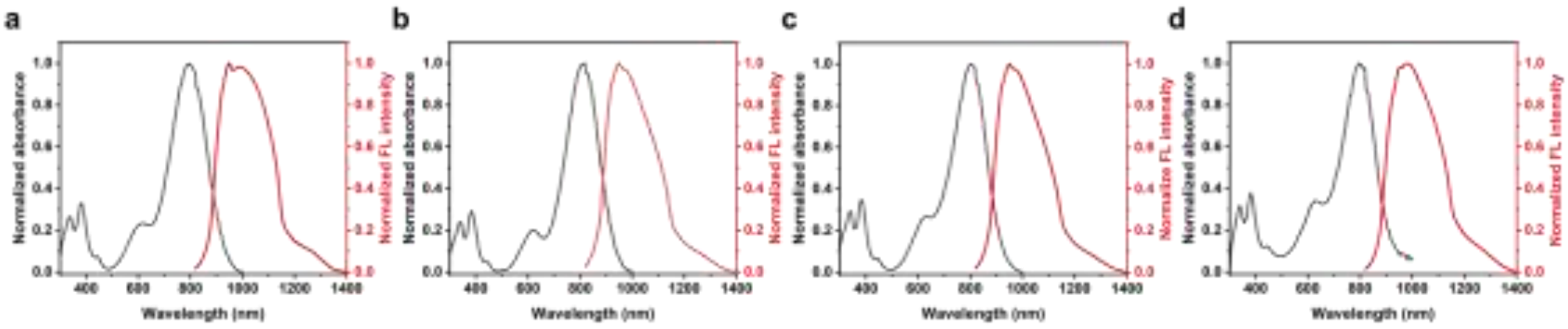

Figure S30. (a-d) Normalized absorption and emission spectra of four compounds (10 μM) in an aqueous formulation containing 10% DMSO and 2% Tween 80.

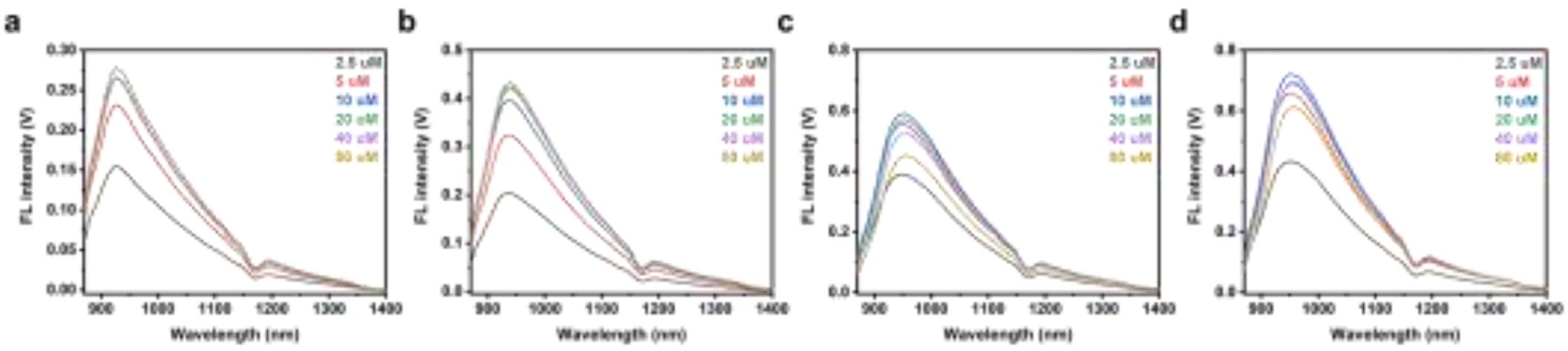

Figure S31. Fluorescence emission spectra of (a) SCU-OX-Me, (b) SCU-OX-Et, (c) SCU-OX-Bu, and (d) SCU-OX-Hex in DCE at different concentrations (2.5, 5, 10, 20, 40, and 80 μM).

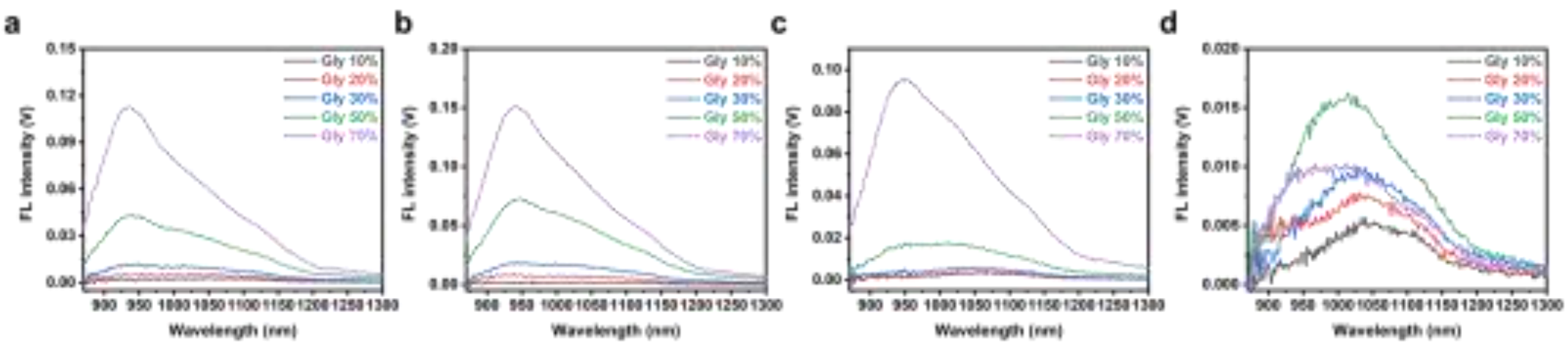

Figure S32. Fluorescence emission spectra of (a) SCU‑OX‑Me, (b) SCU‑OX‑Et, (c) SCU‑OX‑Bu, and (d) SCU‑OX‑Hex measured in glycerol/water mixtures with glycerol volume fractions of 10%, 20%, 30%, 50%, and 70% (v/v).

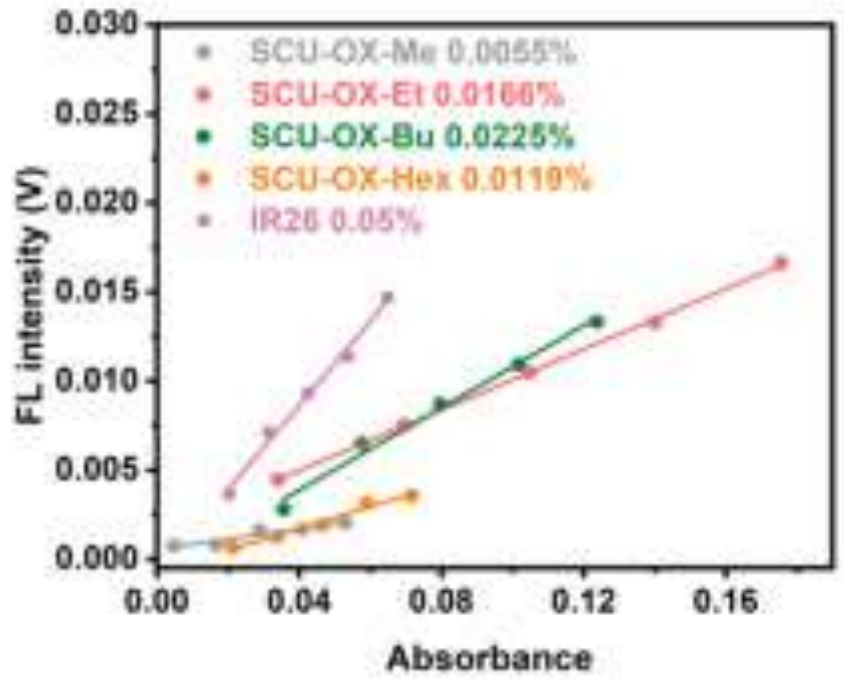

Figure S33. Fluorescence quantum yields of the four compounds in PBS.

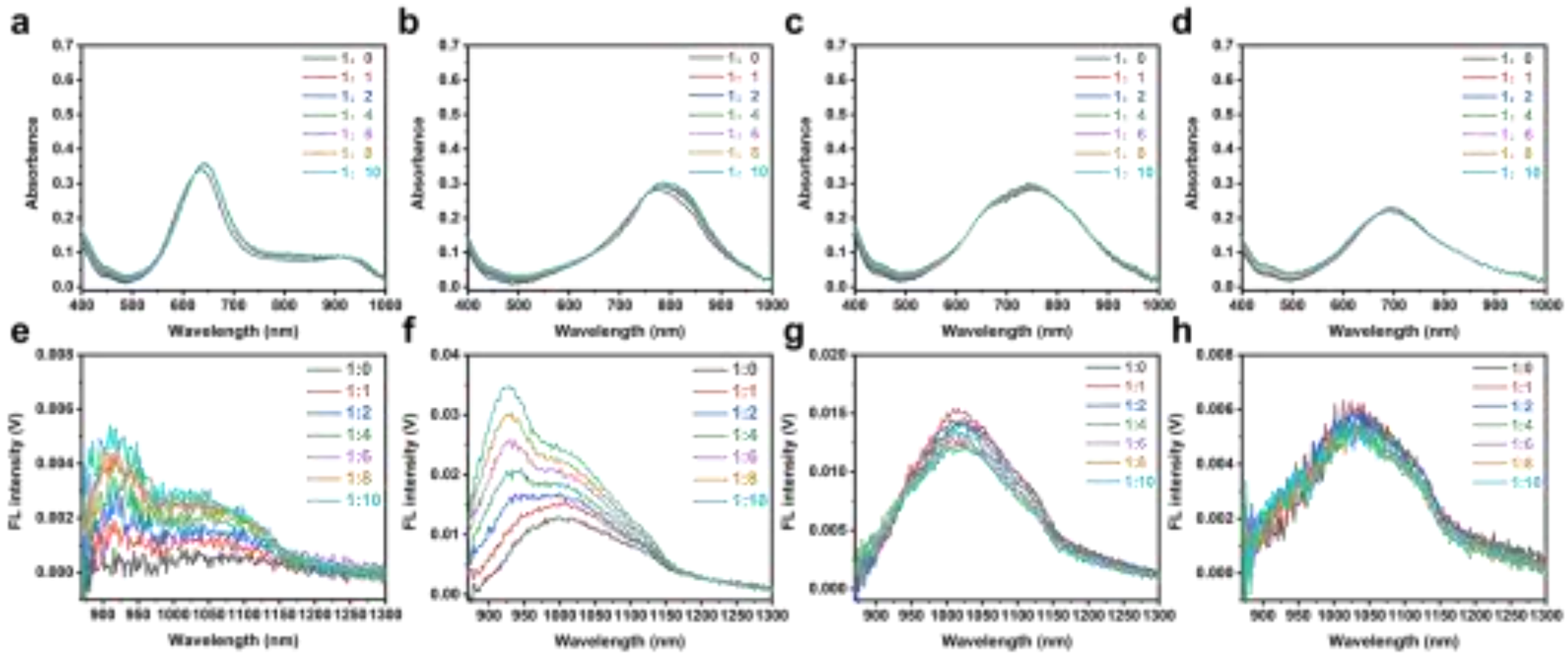

Figure S34. (a-d) Absorption spectra and (e-h) emission spectra of SCU-OX-Me, SCU-OX-Et, SCU-OX-Bu, and SCU-OX-Hex at varying molar ratios with human serum albumin (HSA).

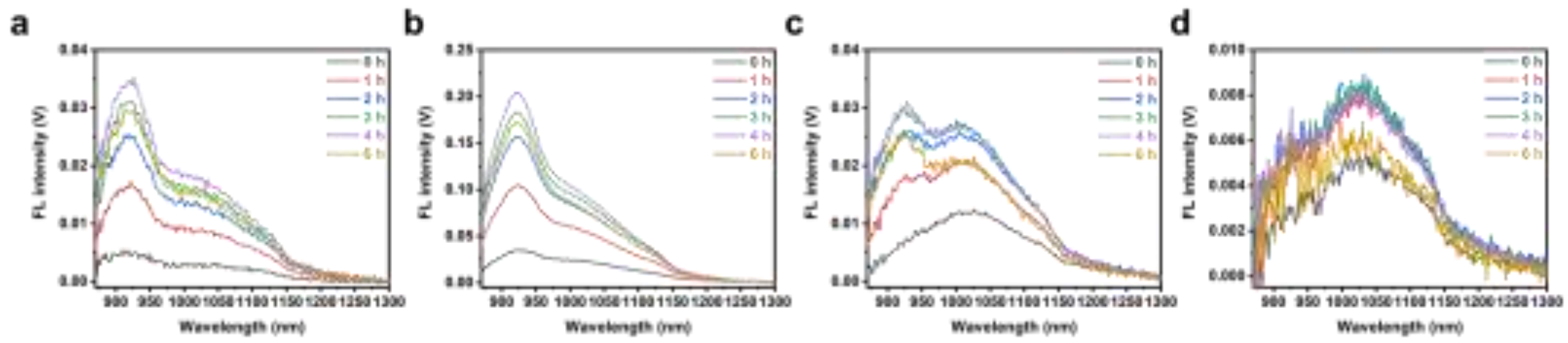

Figure S35. (a-d) Time-dependent fluorescence spectra of SCU-OX-Me, SCU-OX-Et, SCU-OX-Bu, and SCU-OX-Hex with HSA at a 1:10 molar ratio.

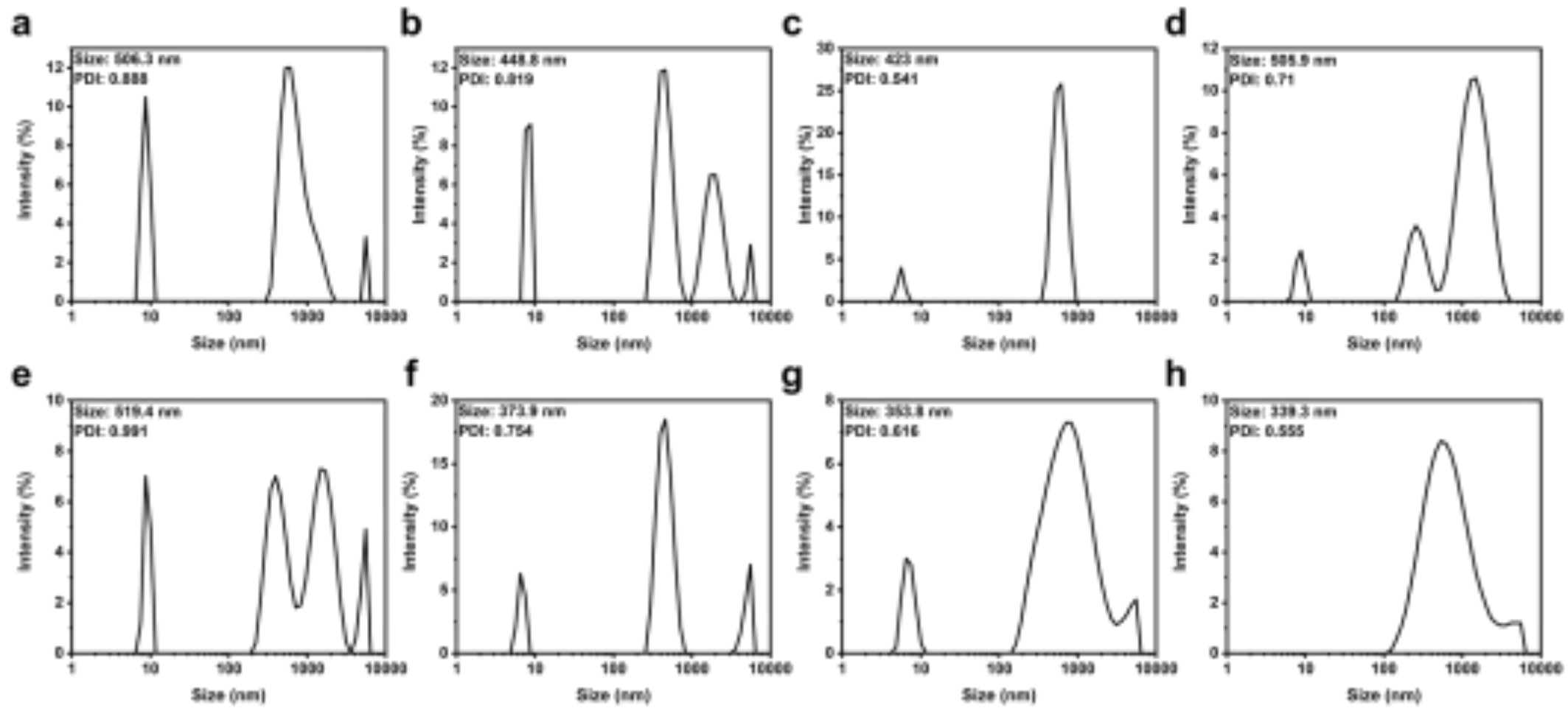

Figure S36. Figure (a-d) Dynamic light scattering of SCU-OX-Me, SCU-OX-Et, SCU-OX-Bu, and SCU-OX-Hex with HSA at a 1:10 molar ratio, (e-h) and corresponding diameters after 6 h of mixing.

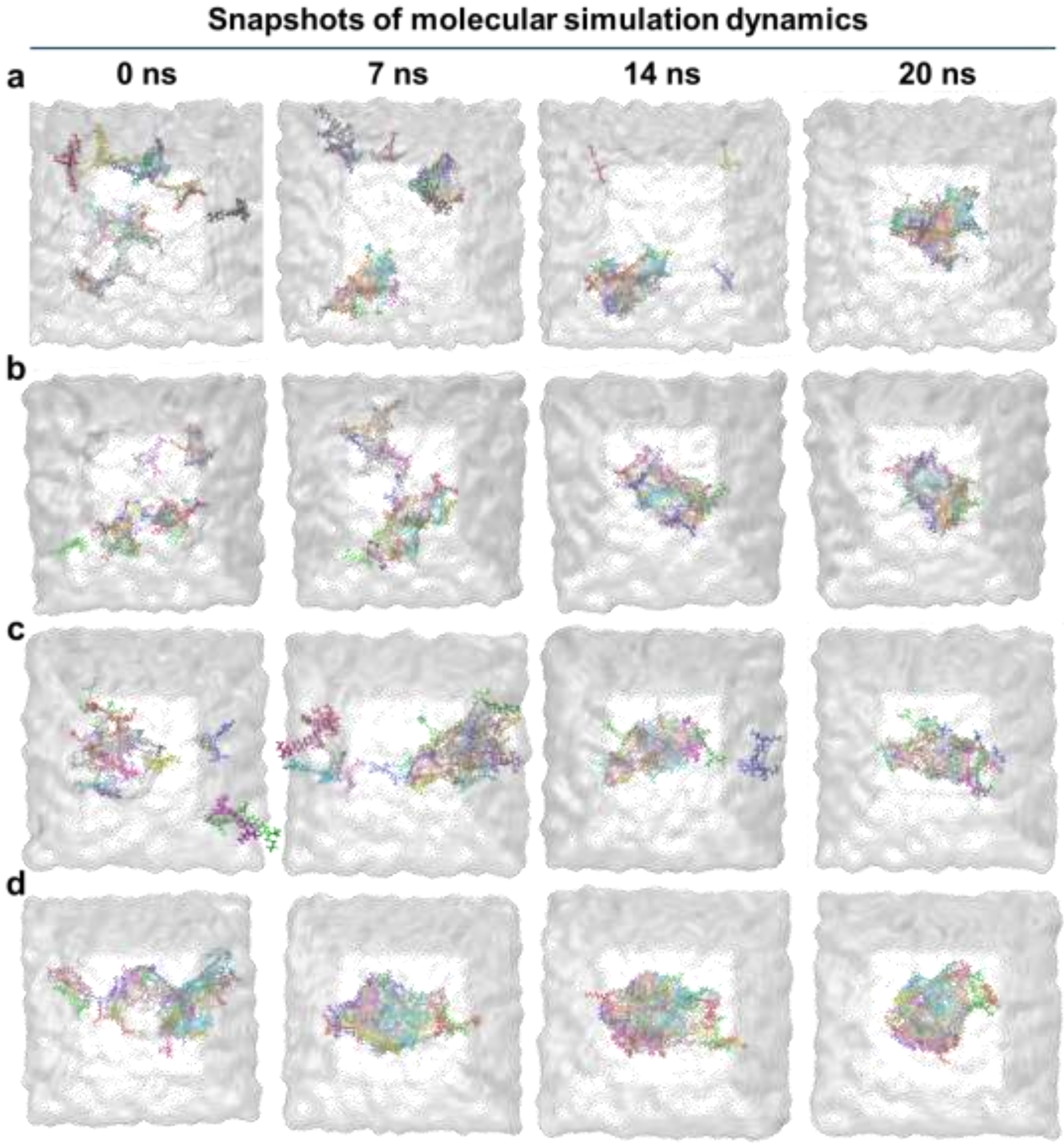

Figure S37. (a-d) Molecular dynamics simulation snapshots of SCU-OX-Me, SCU-OX-Et, SCU-OX-Bu, SCU-OX-Hex in aqueous solution at 0 ns, 7 ns, 14 ns, 20 ns, demonstrating its aggregate evolution over time.

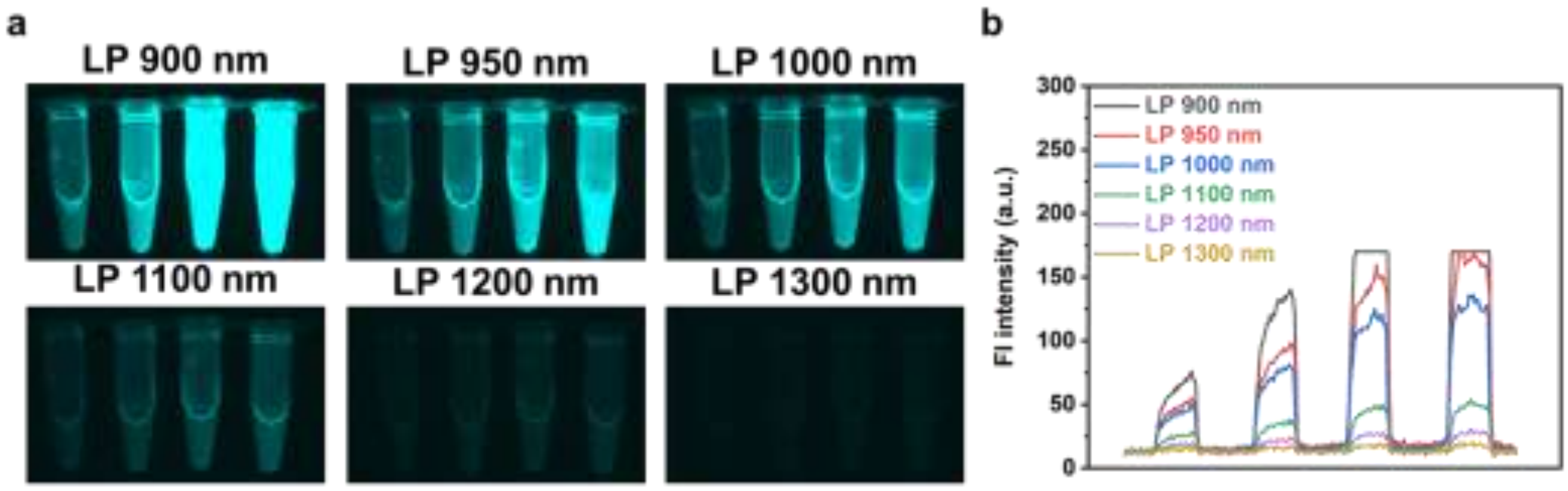

Figure S38. (a) Ep tube images of SCU-OX-Hex with different long pass filters (900 nm, 950 nm, 1000 nm, 1100 nm, 1200 nm, 1300 nm) and (b) fluorescence intensity under the corresponding filter.

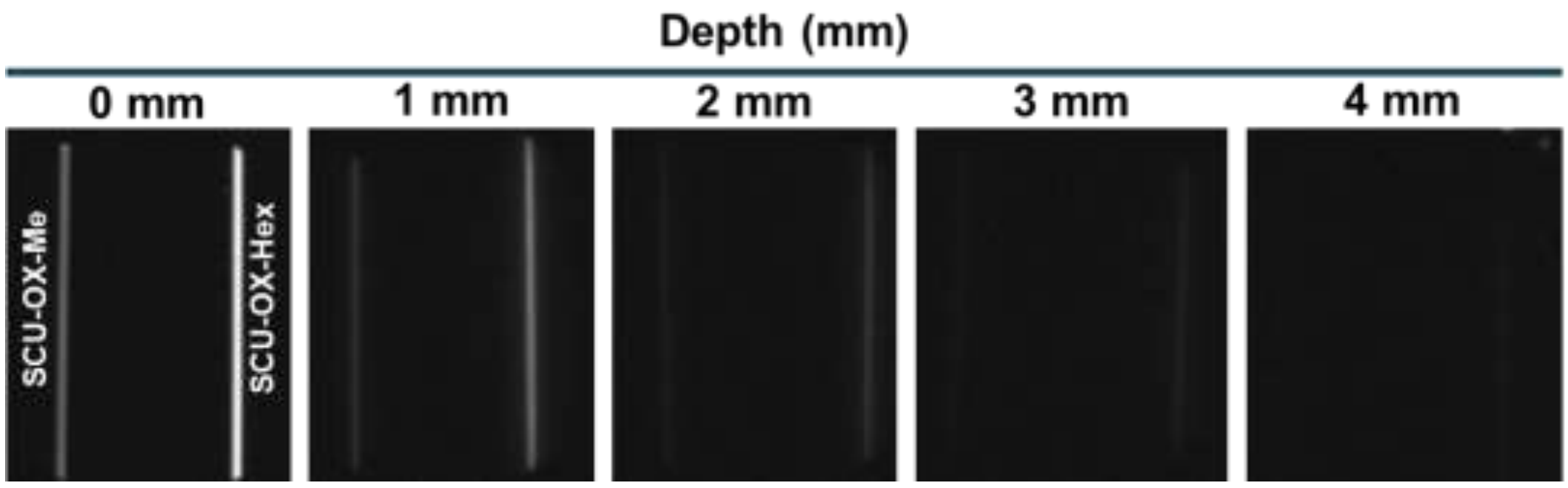

Figure S39. Tissue penetration depth images of SCU-OX-Me and SCU-OX-Hex (10 μM) with different thicknesses of 1 % intralipid.

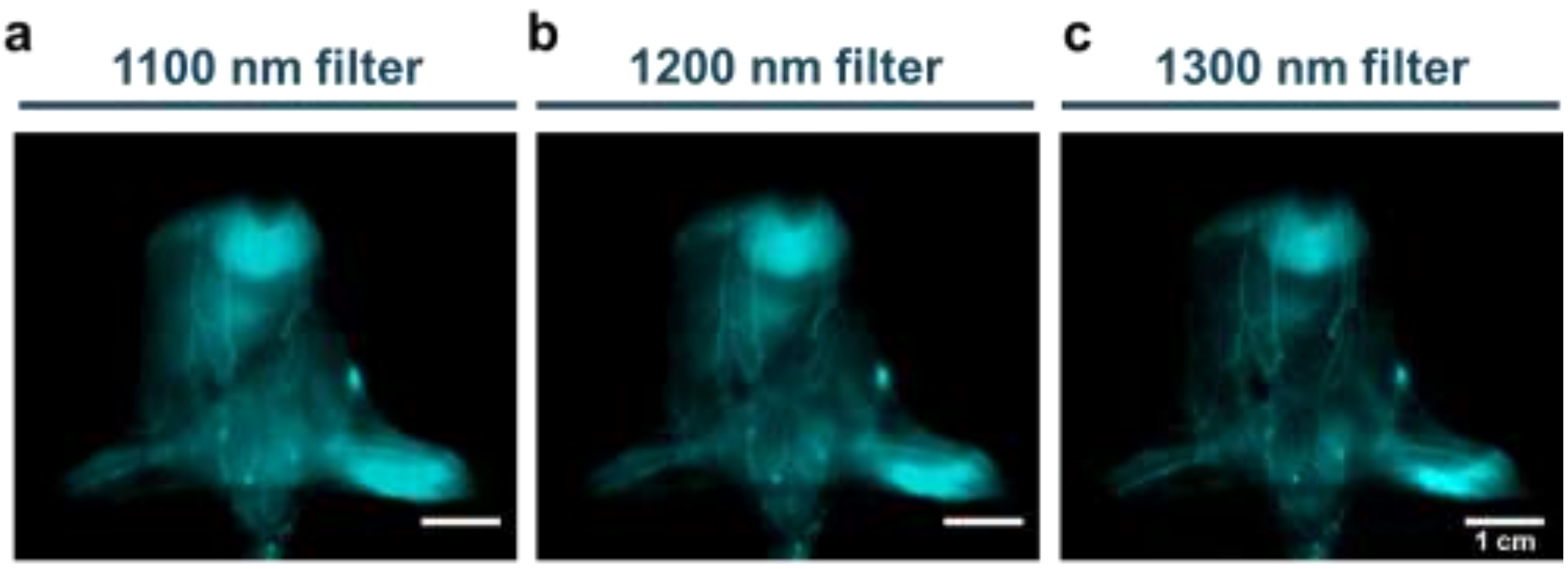

Figure S40. Perivascular imaging of abdominal tumors of SCU-OX-Hex with different long pass filters (1100 nm, 1200 nm, 1300 nm).

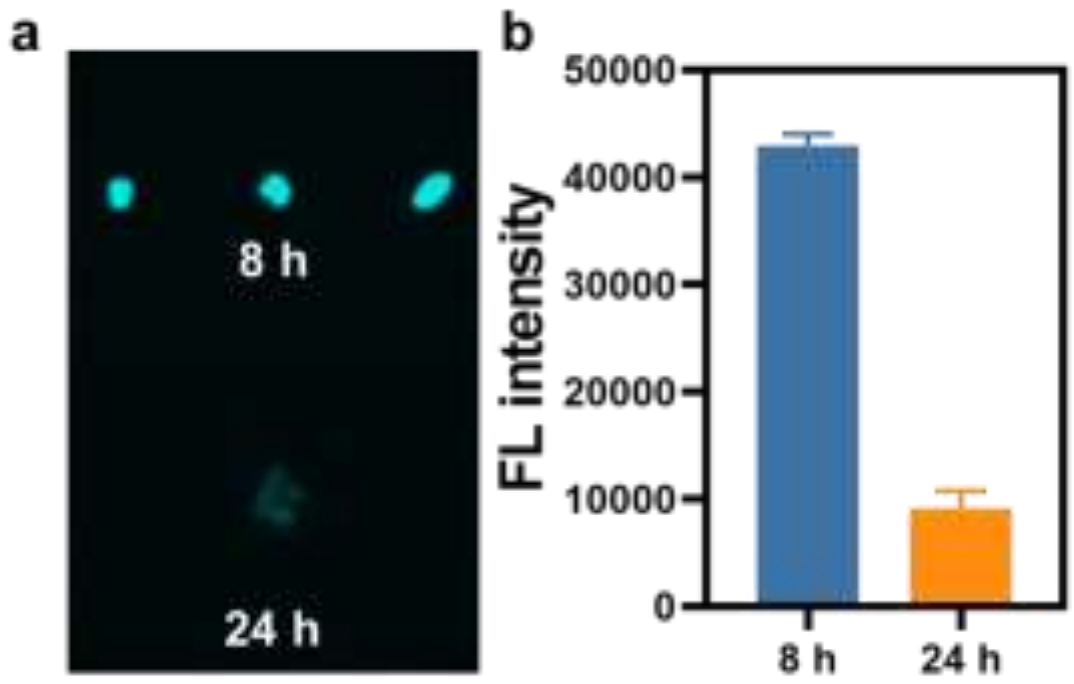

Figure S41. Intestinal imaging in the NIR-II region was performed at 8 and 24 hours, followed by the collection of fecal samples and measurement of their corresponding fluorescence intensities.

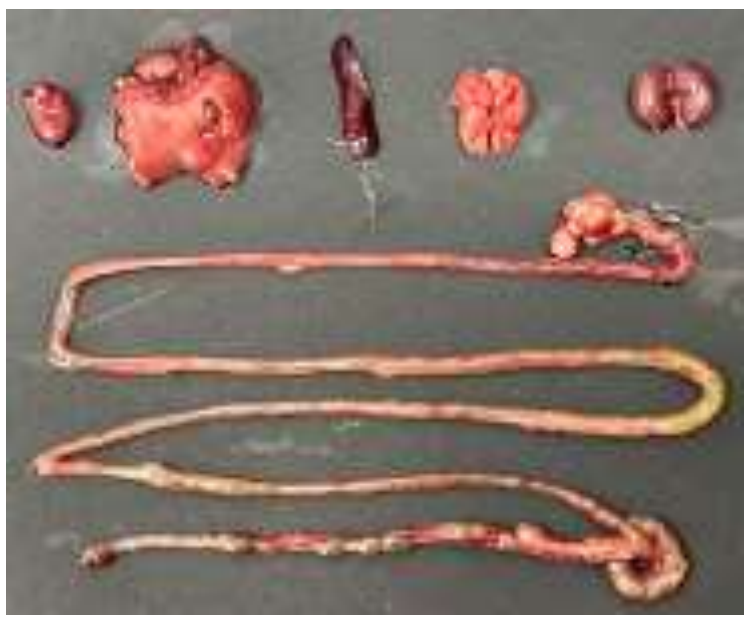

Figure S42. Bright-field photography of ex vivo mouse organs.

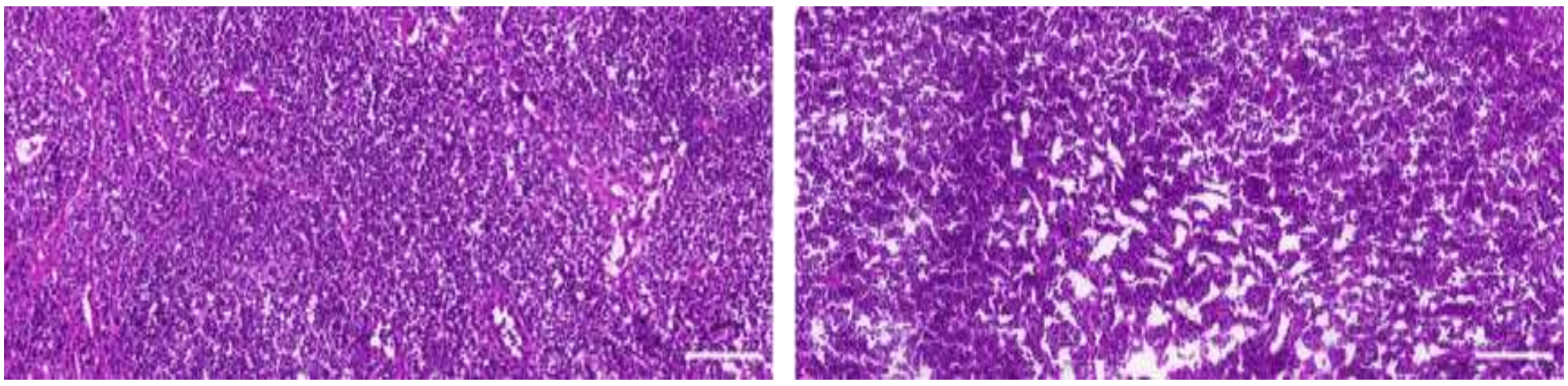

Figure S43. H&E staining of tumor-invaded (a) and normal (b) lymph nodes. Scale bar=100 μm.

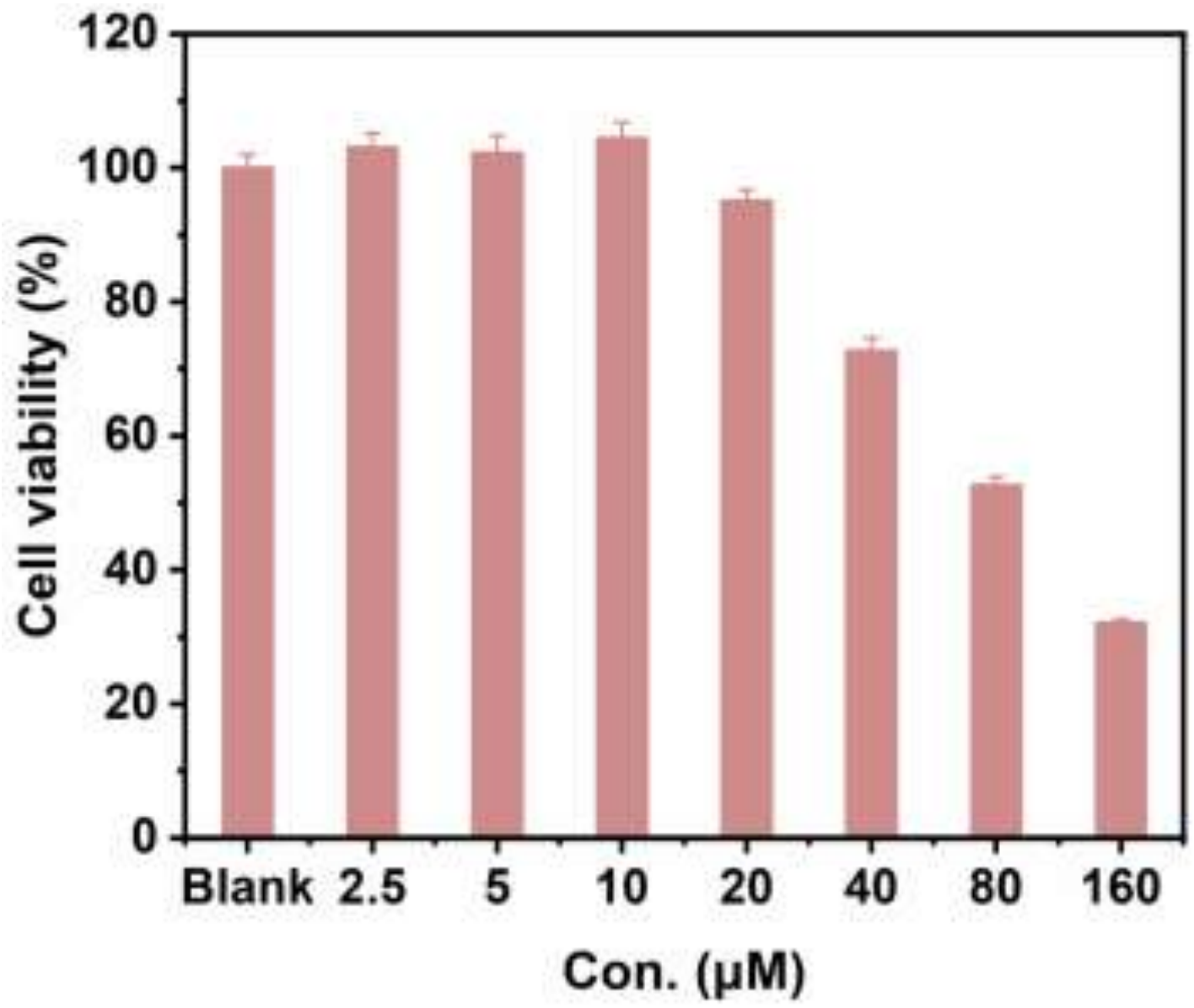

Figure S44. Relative viability of NIH-3T3 cells incubated with various concentrations of SCU-OX-Hex for 12 h.

REFERENCE

[1] Octavi E. Semonin, Justin C. Johnson, Joseph M. Luther, Aaron G. Midgett, Arthur J. Nozik, Matthew C. Beard, *J. Phys. Chem. Lett.* 2010, 1, 2445–2450.
